# The Phase Ib VenObi CNS Study: Chemotherapy-Free Treatment with Venetoclax and Obinutuzumab for Relapsed/Refractory Primary Large B-Cell Lymphoma of the Central Nervous System

**DOI:** 10.3390/cancers18030455

**Published:** 2026-01-30

**Authors:** Julia Wendler, Benjamin Kasenda, Eliza M. Lauer, Kudret Kama, Lisa Kristina Isbell, Dominik Marschner, Florian Scherer, Natalie Malenica, Cora Gloggengiesser, Elke Valk, Elisabeth Schorb, Gerald Illerhaus

**Affiliations:** 1 Clinic of Hematology, Oncology, Stem Cell Transplantation and Palliative Care, Klinikum Stuttgart, 70174 Stuttgart, Germany; 2 Medical Oncology, University Hospital Basel, 4031 Basel, Switzerland; 3 Department of Medicine I, Medical Center—University of Freiburg, Faculty of Medicine, University of Freiburg, 79106 Freiburg, Germanyelisabeth.schorb@uniklinik-freiburg.de (E.S.); 4 Stuttgart Cancer Center—Tumorzentrum Eva Mayr-Stihl, 70174 Stuttgart, Germany

**Keywords:** pharmacokinetics, rrPCNSL, venetoclax, obinutzumab

## Abstract

Background/Objectives: Relapsed and refractory (rr) primary large B-cell lymphoma of the central nervous system (PCNSL) has a dismal prognosis, and the standard of care is not established. The most common genetic imbalance includes the B-cell lymphoma 2 (BCL-2) locus. Methods: We planned a bi-centric phase Ib dose-escalation study with the chemotherapy-free combination of the BCL-2 inhibitor venetoclax and CD20 antibody obinutuzumab for rrPCNSL patients in Germany. The intended treatment consisted of 6 cycles of fixed-dose obinutuzumab at 1000 mg intravenously every 3 weeks, and an oral daily dose of 600, 800, or 1000 mg venetoclax, depending on the planned dosing group, followed by a 12-month venetoclax maintenance period. The primary endpoint was the pharmacokinetics of venetoclax and obinutuzumab in cerebrospinal fluid (CSF). Results: This study was prematurely terminated after registration of 5/15 (33%) patients in dosing group 1 (600 mg oral daily dose of venetoclax) between May 2020 and November 2021. The mean ratio of the concentration of venetoclax in CSF over peripheral blood was 0.55% (±0.28 standard deviation (SD)) and 0.25% (±0.23 SD) for obinutuzumab. Two of five patients achieved complete remission, and each one patient achieved partial remission and stable disease as best response. The median duration of response was 6.5 months (range 0.7–47). Conclusions: Venetoclax and obinutuzumab can penetrate into the central nervous system, but the CSF concentration did not correlate with the outcome. The combination is feasible, tolerable, and may lead to durable responses in selected rrPCNSL patients.

## Simple Summary

Despite substantial advances in the treatment of primary large B-cell lymphoma of the central nervous system (PCNSL), up to one-third of patients fail first-line treatment due to toxicity or early disease progression, and up to 50% of patients eventually relapse, despite achieving complete remission after first-line treatment. In the relapsed/refractory setting (rrPCNSL), the standard of care is not established, and prognosis remains poor. The most common genetic imbalance in PCNSL includes the B-cell lymphoma 2 (BCL-2) locus. The aim of this investigator-initiated phase Ib trial was to assess the pharmacokinetics of the BCL-2 inhibitor venetoclax and the CD20-antibody obinutuzumab in rrPCNSL. We demonstrated that both drugs could penetrate into the central nervous system compartment. Moreover, this chemotherapy-free combination treatment was feasible, tolerable, and may provide durable responses in selected patients.

## 1. Introduction

Primary diffuse large B-cell lymphoma of the central nervous system (PCNSL) is a rare subtype of large B-cell lymphoma of immune-privileged sites that primarily arises and is confined to the central nervous system (CNS) compartment [1,2]. High-dose methotrexate (HD-MTX)-based first-line (1L) treatment, followed by thiotepa-based high-dose chemotherapy with autologous stem cell support (HCT-ASCT), is a widely accepted standard of care (SOC) in eligible untreated patients with PCNSL [3,4,5,6,7]. However, 15–32% of patients fail 1L chemotherapy due to toxicity or progressive disease (PD), and 25–50% of patients with documented complete response eventually relapse, particularly within 2 years from the end of 1L [4,8]. In the relapse or refractory (rr) setting, the SOC is not established, and outcomes remain poor [9]. Only a few prospective trials have investigated targeted therapies with, e.g., the Bruton’s Tyrosine–Kinase inhibitor (BTK) ibrutinib, the immunomodulating agents lenalidomide or pomalidomide or the mammalian Target of Rapamycin (mTOR) inhibitor temsirolimus, either as monotherapies or in combination with the CD20-antibody rituximab in rrPCNSL so far, resulting in a median progression-free survival (PFS) of 2.1–9.2 months [10,11,12,13,14,15,16]. In further treatment lines, a substantial proportion of patients seem ineligible for an intensive salvage treatment regimen due to unresolved treatment-related toxicity of prior anti-lymphoma treatment, age, performance status (PS), or comorbidities [8]. The most common genetic imbalance in PCNSL is gains of 18q21, which include the B-cell lymphoma 2 (BCL-2) locus [17]. Furthermore, the IELSG32 trial of the International Extranodal Lymphoma Study Group has established anti-CD20-directed therapy with rituximab in PCNSL [18]. Consequently, the combination of venetoclax, a selective orally bioavailable small molecule inhibitor of BCL-2, and the anti-CD20 antibody obinutuzumab, which provides additional pharmacodynamic features in comparison to rituximab, seems to be a promising chemotherapy-free combination in rrPCNSL. Although these agents are already approved in indolent lymphoma, demonstrating acceptable toxicity profiles, it remains unclear whether these agents can penetrate the blood–brain barrier to unfold a robust anti-lymphoma effect in the CNS affected by aggressive large B-cell lymphoma. Thus, we conducted a single-arm, bi-centric, dose escalation phase Ib trial in Germany to investigate the pharmacokinetics (PKs) of venetoclax and obinutuzumab in cerebrospinal fluid (CSF). Secondary endpoints included efficacy, feasibility, and safety.

## 2. Materials and Methods

### 2.1. Patients and Study Design

We conducted this phase Ib trial at two German tertiary referral centers experienced in the treatment of PCNSL patients. Key inclusion criteria were: (1) biopsy proven CD20-positive PCNSL at diagnosis or relapse/progression (re-biopsy at study inclusion was not mandatory, but strongly recommended if remission exceeded 24 months), (2) at least one prior HD-MTX (minimum dose of ≥1 g/m^2^) containing chemotherapy received, (3) age at inclusion 18–80 years and Eastern Cooperative Oncology Group (ECOG) PS ≤ 3 or up to 85 years in case of ECOG PS 0-1. Key exclusion criteria were: (1) a lymphoma relapse outside the CNS, (2) prior exposure to the investigational medicinal products (IMPs) obinutuzumab or venetoclax, (3) other additional anti-lymphoma treatment, (4) active hepatitis B or C, as well as Human–Immunodeficiency Virus (HIV) seropositivity, or other chronic immunosuppression, and (5) the administration of moderate or strong CYP3A inhibitors or inducers within one week of the initiation of venetoclax. Steroids were allowed at registration, but were tapered to stop as soon as treatment had started if possible. Particularly, within the first 6 weeks of on-trial treatment, co-medication with CYP3A4 inhibitors and CYP3A4 inducers, P-gp inhibitors, and BCRP inhibitors was prohibited to avoid interference with PK and DLT assessments.

This study was approved by the Ethics committee of the Landesärztekammer Baden-Württemberg (AM-2019-028-ff) and, subsequently, by the Ethics committee of the Albert-Ludwigs-Universität Freiburg. All patients provided written informed consent prior to participation in this trial. All procedures were performed in accordance with institutional and national standards, and in accordance with the Helsinki Declaration of 1975, as revised in 2008.

**Figure 1:**
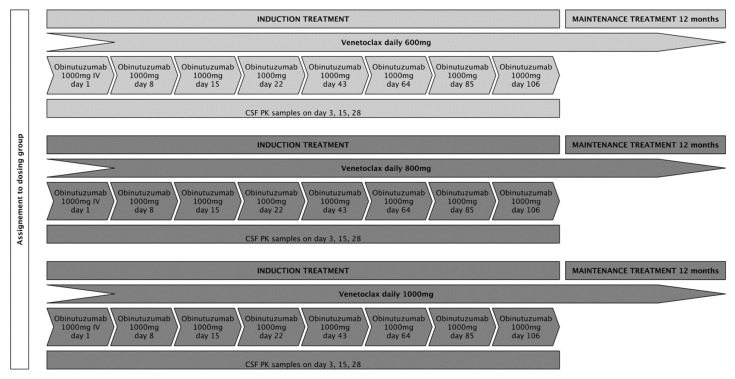
The planned treatment schedule for the respective dosing groups.

We aimed to include 15 patients, 5 patients in each of the 3 intended dose-levels (venetoclax 600 mg, 800 mg, and 1000 mg). Patients at dose level 1 were to receive an oral daily dose of 600 mg venetoclax in combination with six cycles of fixed-dose obinutuzumab at 1000 mg intravenously every 3 weeks as induction treatment (except for cycle 1, in which obinutuzumab was administered on days 1, 8, and 15), followed by a 12-month maintenance treatment with an oral daily dose of 600 mg venetoclax for patients who achieved at least stable disease (SD) following induction treatment. Patients in dose levels 2 and 3 were to receive treatment as described above with daily oral venetoclax doses of 800 mg and 1000 mg, respectively. To assure consistency for pharmacokinetic (PK) analyses, application of IMPs was set at fixed time points during the first 4 weeks of treatment. Treatment adherence for venetoclax was documented in patient diaries and drug count every 3 weeks. A treatment schedule is displayed in Figure 1. Response assessments (RAs) with gadolinium-enhanced magnetic resonance imaging were scheduled on day 22, day 43, day 85, and at the end of induction treatment (day 127). During maintenance treatment, RAs were performed every 8 weeks for the first 6 months and every 12 weeks thereafter until the end of this study. The response evaluation was assessed by local neuro-radiological evaluation, following the International PCNSL Collaborative Group (IPCG) response criteria [19].

### 2.2. Sample Acquisition, Handling, Storage, and Analysis of Pharmacokinetics

Regarding the primary endpoint PKs of venetoclax and obinutuzumab in CSF, peripheral blood (PB) samples were drawn at the following time points: day 1 (pre-dose and post-dose), days 3, 15, 28 (each pre-dose), and at the first maintenance visit (4 weeks after the end of induction treatment, pre-dose), as well as the CSF samples on days 3, 15, and 28 (all pre-dose). Pre-dose sampling was performed within a maximum of 3 h prior to IMP application, and post-dose sampling was performed 30 min after termination of obinutuzumab infusion. Venetoclax PK samples were collected, handled, and stored as follows: PB samples were collected in Kalium-EDTA tubes, and CSF samples were collected in Sarstedt tubes. The PB samples were then centrifuged at 1500× *g* for 10 min at 4 degrees Celsius. The supernatants of the PB samples were pipetted into cryo vials, the CSF samples were transferred into a Tween dilution, pipetted into cryo vials, and then both were stored at below −70 degrees within two hours of collection, until shipped on dry ice to the designated laboratory from AbbVie Inc. (North Chicago, IL, USA) for analyses. Obinutuzumab PK samples were collected, handled, and stored as follows: PB samples were collected in serum tubes, then centrifuged at 1500× *g*, and CSF samples were collected in Sarstedt tubes, then centrifuged at 2000× *g*, each for 10 min at 4 degrees Celsius within one hour of collection. The supernatants were pipetted into cryo vials and stored at below −70 degrees within two hours of collection, until shipped on dry ice to the designated laboratory from Roche Pharma AG (Basel, Switzerland) for analyses. For further information on sample handling and storage, refer to the Supplementary Material. All patients with at least one paired sample of CSF and PB were considered for analyses. The ratio of the CSF concentration over the PB concentration was calculated for all time points with matched samples to display the CSF penetration of the respective IMPs. Moreover, the plan was to summarize the frequency of patients in whom the concentration of venetoclax in the CSF at each time point was ≥4% of the steady state concentration of PB, which was based on in vitro apoptosis experiments [20,21]. For obinutuzumab, the cut-off was set at ≥1%, based on data on the CD20-antibody rituximab [22]. Determination of the analyte venetoclax (A1195425) in Human Plasma K2 EDTA and CSF was performed using Liquid/Liquid Extraction followed by Liquid chromatography–mass spectrometry (LC-MS)/MS detection in a designated laboratory from AbbVie Inc. Determination of the analyte obinutuzumab (RO5072759/rhuMab anti-CD20) in Human Serum and CSF was analyzed using a quantitative Enzyme Linked Immunosorbent Assay (ELISA) in a laboratory designated by Roche Pharma AG.

### 2.3. Endpoints and Statistical Analyses

We used dose-limiting toxicities (DLTs, assessed following the Common Terminology Criteria for Adverse Events version 5.0 (CTCAE v5.0)) in the escalation of the dosing groups for our sample size calculation. The following events were considered DLT: (1) trial therapy-related death, (2) grade 4 neutropenia not resolved after 14 days despite growth factor support, (3) grade 3 to 4 febrile neutropenia, (4) grade 4 thrombocytopenia not resolved after 14 days, and (5) grade 2 or higher bleeding associated with thrombocytopenia. Moreover, any other grade 3 or higher hematological or non-hematological AE related to one or both IMPs that did not resolve to at least grade 2 or to baseline value within three weeks since onset by complete drug discontinuation and supportive care, if applicable, was considered a DLT, except for alopecia and nausea/diarrhea adequately treated. Patients had to have received at least 80% of the scheduled IMP dose in the respective dosing groups and must have completed all scheduled safety evaluations within the first 6 weeks of induction treatment to be included in the DLT appraisal. If a patient was not assessable for DLT, the patient would have been replaced to guarantee that there were always five patients assessable for DLTs in each of the intended dosing groups. We used the Bayesian optimal interval (BOIN) design [23] to determine the feasibility for dose escalation and de-escalation regarding the intended 3 dosing groups of venetoclax. The target DLTs were set at 30%, which translated into the following decision boundaries: if ≤1 DLT occurred at a certain dose level, the venetoclax dose would be escalated to the next higher dose level; if ≥2 DLTs occurred, the dose would have been de-escalated to the next lower dose level (minus 200 mg oral daily venetoclax dose); if ≥4 DLTs occurred at a certain dose level, this study would have been prematurely terminated. The safety population included all patients who had received at least one dose of IMP, thus it is equivalent with the full analysis set (FAS).

Further endpoints included best lymphoma response achieved during induction treatment, according to the IPCG response criteria; progression free survival 1 (PFS1), defined as time from start of induction treatment until PD, relapse, or death, whichever occurred first; PFS2, defined as time from start of maintenance treatment until PD, relapse, or death (patients not reaching maintenance treatment were excluded from PFS2 analysis); failure free survival (FFS), defined as time from treatment start until PD, death, or study termination due to toxicity; time to initiation of subsequent treatment, defined as time from treatment start until initiation of subsequent anti-lymphoma off-trial treatment for whatever reason; and sustained response time for patients achieving complete (CR) or partial remission (PR), defined as time from CR/PR until PD or death, as well as overall survival (OS), defined as time from start of induction treatment until death from any cause. Efficacy endpoints were analyzed for all registered patients for whom treatment was started, denoted as FAS. For time-to-event data (PFS1/2, FFS, and OS), patients were censored at the last date of follow-up if they did not experience the respective event beforehand. Respective survival probabilities were calculated at the 6-, 9-, and 12-month landmarks.

Initially, we also planned to conduct next-generation sequencing based on archival formalin-fixed tissue using the FoundationOne^®^ Heme platform. However, these additional analyses were not conducted because the trial was terminated early due to slow recruitment in November 2021.

Due to the small sample size, the main outcome data (PK, response, DLT, and survival endpoints) are presented for each patient individually in appropriate tables or plots. Given that this study is entirely exploratory in nature, statistical analyses for hypothesis testing were not performed. Continuous data was described by the arithmetic mean and standard deviation or the median and absolute range. Categorical data was described by frequency and proportion. Correlations were based on linear regression analyses. Statistical analyses were performed with GraphPad Prism 10.4.1.

## 3. Results

We recruited five patients between May 2020 and November 2021 at two German sites. For details on baseline characteristics for the individual patient refer to Table 1.

### 3.1. Pharmacokinetics

**Figure 2:**
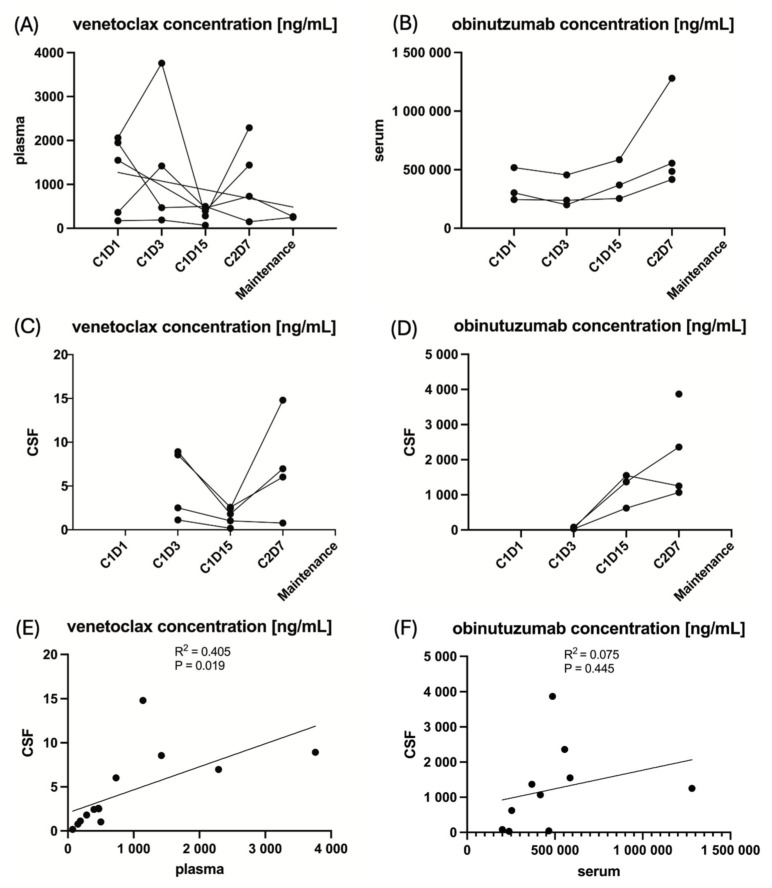
The pharmacokinetics of the IMPs venetoclax and obinutuzumab in peripheral blood and cerebrospinal fluid at the respective time points of sampling. Note: Due to the small sample size of five patients, these analyses are entirely exploratory. (**A**) The venetoclax concentration in plasma for the individual patient. (**B**) The obinutuzumab concentration in serum for the individual patient. (**C**) The venetoclax concentration in cerebrospinal fluid for the individual patient. (**D**) The obinutuzumab concentration in cerebrospinal fluid for the individual patient. (**E**) The linear regression of venetoclax concentration in cerebrospinal fluid to peripheral blood. (**F**) The linear regression of obinutuzumab concentration in cerebrospinal fluid to peripheral blood.

The concentrations of venetoclax and obinutuzumab in CSF and PB at the respective time points are provided in Figure 2, and for the individual patient, including missing data points, are provided in Supplemental Table S1 in the Supplementary Materials.

The mean ratio of the concentration of venetoclax in CSF over the concentration of venetoclax in PB was 0.55% (±0.28 standard deviation) and 0.25% (±0.23 standard deviation) for obinutuzumab. The targeted ratio of ≥4% for venetoclax and ≥1% for obinutuzumab was not reached at any time point in any patient. However, unfortunately, the venetoclax and obinutuzumab CSF samples were analyzed outside the supported long-term storage stability data. A total of 4/5 (80%) patients received ≥80% of the intended venetoclax dose during the first 2 treatment cycles in which PK samples were collected, and 3/5 (60%) patients received ≥80% of the intended obinutuzumab dose. Detailed information on drug exposure for the individual patient is provided in Table 2. Concentrations between CSF and PB correlated significantly for venetoclax (R^2^ = 0.405, *p* = 0.019) in contrast to obinutuzumab (R^2^ = 0.075, *p* = 0.445), as shown in Figure 2. Moreover, neither the mean venetoclax concentration nor the mean obinutuzumab concentration in CSF significantly correlated with PFS (R^2^ = 0.002, *p* = 0.895 and R^2^ = 0.012, *p* = 0.914, respectively), Supplemental Figure S1 in the Supplementary Materials.

### 3.2. Efficacy

Best response achieved was CR in 2/5 (40%) patients [after 21 days and 9.9 months from the start of on-study treatment], PR in 1/5 (20%) [after 21 days from the start of on-study treatment], SD in 1/5 (20%) patients, and PD in 1/5 (20%) of patients. For the three patients achieving PR or CR, sustained response time was 0.7 and 6.5 months until PD in two patients and 47.6 months until last follow-up in the other patient.

Two-thirds of the patients with documented response received concomitant corticosteroid treatment at registration within this trial. Corticosteroids were tapered to stop 3 and 9 days after the start of on-study treatment. Cumulative dexamethasone doses were 72 and 78 mg. The two patients who did not respond to on-study treatment continued corticosteroids beyond the end of on-study treatment.

**Figure 3:**
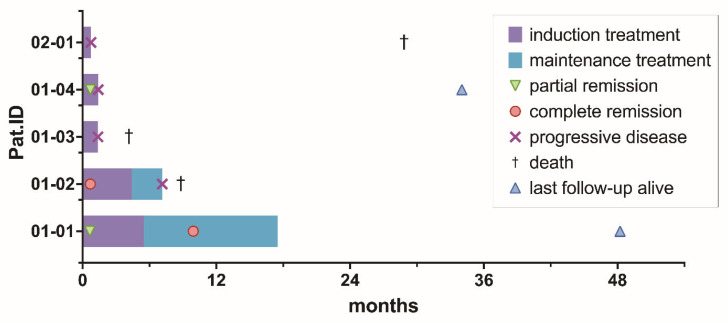
Treatment phase, time to best response, progression-free, and overall survival per patient.

After a median follow-up time of 28.7 months (range 4.2–48.2), 3/5 patients (60%) died at 4.2, 9.0, and 34.2 months after registration. A total of 2/3 (67%) patients died due to PCNSL, and 1/3 (33%) patients died due to an unknown reason. PFS1 rates were 40%, 20%, and 20% at 6, 9, and 12 months. OS rates were 80%, 80%, and 60% at 6, 9, and 12 months. PFS2 was not analyzed, given only 2/5 (40%) of patients were delivered into maintenance treatment, and FFS was not analyzed in addition to PFS, as no patient prematurely ended treatment due to toxicity. Median PFS and OS were 1.4 months (range 0.8–7.1) and 8.8 months (range 4.2–28.9), respectively. A total of 3/4 patients experiencing PD during on-study treatment received the following off-study salvage treatment: chemotherapy with DeVIC (dexamethasone, infusional etoposide, ifosfamide, and carboplatin) [n = 3] plus immunotherapy with rituximab [n = 2] and radiotherapy [n = 2] after a median time of 43 days (range 42–55) from the start of on-study treatment. An overview of the duration of treatment and survival data for the individual patient is displayed in Figure 3.

### 3.3. Safety

A total of 3/5 patients (60%) of dose level 1 were assessable for DLTs, but no DLTs were reported. A total of 2/5 patients did not receive at least 80% of the scheduled IMP dose at 6 weeks due to early treatment failure and thus were not assessable for DLTs. An overview of further AEs that occurred during this trial, as per the Medical Dictionary for Regulatory Activities System Organ Class (MedDRA SOC) version 23.0, is provided in Table 3. All patients experienced at least one AE. The most frequently reported AEs were thrombocytopenia (all patients), leukopenia, neutropenia, and lymphopenia (each at 4/5 (80%) patients). In 2/5 (40%) of the patients, treatment was delayed up to a maximum of 4 weeks, and subsequently, venetoclax was reduced as per clinical trial protocol (CTP) regulations due to respective adverse events. Reasons for treatment hold were decreased neutrophils (grade 4) in two patients and decreased platelets (grade 4) in one patient. The minimum continued oral daily venetoclax dose was 200 mg in one patient and 400 mg in the other patient. Serious adverse events (SAEs) occurred in 2/5 (40%) patients: infectious complications (pneumonia grade 3 [n = 1], sepsis grade 4 [n = 1]), nervous system disorders (subarachnoid bleeding grade 1 [n = 1]), and general disorders (asthenia and pyrexia grade 3 [n = 1]). Only the SAE pneumonia was considered as possibly related to both administered IMPs. No fatal adverse event occurred during the conduct of this trial.

## 4. Discussion

Within this investigator-initiated bi-centric phase Ib study, we evaluated the chemotherapy-free combination of the BCL-2 inhibitor venetoclax and the CD20-antibody obinutuzumab in patients with rrPCNSL. To our knowledge, this is the first prospective trial to evaluate this chemotherapy-free combination treatment in patients with rrPCNSL. Moreover, clinical data on the pharmacokinetics of venetoclax in CSF is limited to a few small series of acute leukemia patients, and is non-existent for obinutuzumab. Our results show that both IMPs can penetrate into the CNS compartment. The ratios of the concentration in CSF over the concentration in PB did not reach the intended margins of ≥4% for venetoclax and ≥1% for obinutuzumab. However, these margins were only based on previously published in vitro apoptosis experiments for venetoclax usage in acute myeloid leukemia and adapted from pharmacokinetic data on the CD20-antibody rituximab, hence must rather be interpreted as reference benchmarks than validated efficacy cut-offs. Consequently, these thresholds are only transferable to anti-lymphoma efficacy in PCNSL to a very limited extent [21,22]. Of note, the outcome was not associated with the CSF concentration of the IMPs within this trial. Badawi et al. demonstrated the penetration of venetoclax into the CNS compartment in 46 patients with pediatric leukemia receiving an adult equivalent oral daily dose of 400–800 mg. The mean ratio of CSF to PB was 0.25% in this cohort [24]. Jian et al. presented a median CSF to PB ratio of 0.74% among 13 patients with adult acute myeloid leukemia [25]. Notably, both studies present CSF to PB ratios that are in line with our results. In contrast to previously published data for venetoclax in patients with acute myeloid leukemia [25], we could demonstrate significant linear correlation for venetoclax in CSF and PB concentrations, which is likely attributed to the disrupted blood–brain barrier in patients with CNS lymphoma and suggests that higher oral daily doses may substantially increase the CSF bioavailability of venetoclax. However, whether a higher oral daily venetoclax dose effectively improves outcomes in this patient population needs to be evaluated in future clinical trials, including a larger patient cohort. Data on the penetration of CD20-antibodies in CNSL is scarce, and to our knowledge CSF to PB ratio is only described for rituximab to be 0.1% [22]. Our PK results demonstrate comparable results for obinutuzumab.

In terms of efficacy, we could demonstrate an ORR of 60% (3/5 patients), including two patients achieving CR as the best response. Within the limits of inter-trial comparison, these results are in line with other prospective phase I–II trials evaluating chemotherapy-free salvage treatment with BTK-inhibitors in rrCNSL, which reported ORRs of 52–77% [11,16,26,27]. Moreover, small phase I trials demonstrated an ORR of 48% for the immunomodulator pomalidomide and 62% for the combination of lenalidomide and rituximab [14,15]. Also, early clinical trials including patients with CNS lymphoma demonstrated CNS penetration for both substance classes: the CSF to PB ratios were 1–7% and 0.6–5.8% for the BTK-inhibitors ibrutinib and zanubrutinib [28,29], and 0–49% and 17–19% for the immunomodulators lenalidomide and pomalidomide [14,15]. Nevertheless, these new agents fail to achieve sustained responses, with median PFS ranging from 4.8 to 7.8 months. Although median PFS was still inferior within our study, we could demonstrate a sustained complete response for 47 months until the last follow-up for one patient, suggesting the possibility of durable responses with this combination treatment. In our opinion, the bias of concomitant corticosteroid treatment on the demonstrated efficacy results is very limited, given that only 2/3 patients who achieved radiographical responses received initial concomitant corticosteroid treatment, which was tapered to stop at a maximum of 9 days from the start of on-trial treatment.

The toxicity profile of the combination of venetoclax and obinutuzumab is comparable to the results of its usage in indolent lymphoma [30]. Yet, conclusions regarding the optimal venetoclax dose cannot be drawn from the safety results within this study, given that only 3/5 registered patients were assessable for DLTs. No treatment-related death was reported. However, dose modifications were necessary due to hematological toxicity in 2/5 (40%) patients.

The major limitation of this study is the small cohort due to the premature termination of the trial based on slow recruitment and consecutive withdrawal of funding, as well as the open-label design. Consequently, conclusions on feasibility, efficacy, and safety can only be drawn to a very limited extent, and comparisons with efficacy data from other studies can only be preliminary. In aiming for better recruitment in this challenging study population in future trials, future trials should be conducted at a larger number of study sites. Also, we were not able to correlate efficacy results to different venetoclax dose levels and to gene alterations detected in formalin-fixed tissue, as intended within the study design. Moreover, the CSF for venetoclax and obinutuzumab samples were analyzed outside the established long-term storage stability data, which may have resulted in the underestimation of true PB and CSF concentrations and substantially limits comparability to pre-existing PK data.

## 5. Conclusions

In summary, the phase Ib VenObi CNS trial demonstrated that both the CD20-antibody obinutuzumab and the BCL-2 inhibitor venetoclax can penetrate into the CNS compartment. Furthermore, this chemotherapy-free combination treatment seems feasible in patients with rrPCNSL. In terms of efficacy, the combination may provide durable responses in selected patients. Whether increased doses of venetoclax may be beneficial in terms of efficacy, or whether additional drugs could improve ORR and provide durable responses, needs to be evaluated in future clinical trials.

## Figures and Tables

**Table 1:** Baseline characteristics at VenObi registration.

Pat.ID	Venetoclax Dose Level	Sex	Age at Inclusion (Years)	ECOG PS at Inclusion	Concomitant Steroid Use	Therapy Lines Prior to VenObi	Treatment Prior to VenObi
01-01	1	Male	77	1	no	1	1L MARTA, HCT-ASCT (Bu/TT)
01-02	1	Female	52	1	Yes	2	1L Freiburg Protocol, HCT-ASCT (BCNU/TT); 2L MATRix, 2nd HCT-ASCT (R/Bu/TT)
01-03	1	Male	68	2	Yes	1	1L MARTA. HCT-ASCT (R/Bu/TT)
01-04	1	Male	57	0	Yes	1	1L MATRix, HCT-ASCT (BCNU/TT)
02-01	1	Female	51	1	Yes	2	1L MATRix; 2L R-DeVIC, HCT-ASCT (BCNU/TT)

1L = first-line treatment; 2L = second-line treatment; MARTA = high-dose (HD)-MTX, HD–Cytarabine (AraC), rituximab; HCT-ASCT = high-dose chemotherapy and autologous stem cell transplantation; Bu = Busulfan; TT = Thiotepa; Freiburg Protocol = sequential rituximab, HD-MTX and HD-AraC/TT; MATRix = HD-MTX, HD-AraC, TT, rituximab; BCNU = Carmustin; R-DeVIC = rituximab, dexamethasone, infusional etoposide, ifosfamide, and carboplatin.

**Table 2:** Drug exposure.

Pat.ID	1st Cycle		1st and 2nd Cycle		Notes
	Venetoclax	Obinutuzumab	Venetoclax	Obinutuzumab	
01-01	90.5%	100%	94.4%	100%	Venetoclax daily dose was reduced to 400 mg due to toxicity beginning with cycle 3
01-02	100%	100%	100%	100%	Venetoclax daily dose was reduced to 400 mg beginning with cycle 4 and to 200 mg in cycle 5 due to toxicity
01-03	93.7%	66.7%	96.8%	75%	D15 of obinutuzumab was not applied due to infection
01-04	100%	100%	100%	100%	
02-01	90.5%	100%	45.2%	75%	pEOT after the 1st cycle

The numbers are the ratio of the received dose of the intended dose during the 1st and 1st and 2nd cycle of venetoclax/obinutuzumab; pEOT= premature end of treatment.

**Table 3:** Adverse Events.

		AE Incidence		SAE Incidence
MedDRA SOC	Preferred Term	All Grades	Grade ≥ 3	
Number of patients with at least one (S)AE		5 (100%)	5 (100%)	2 (40%)
Infections and infestations				
	Sepsis	1 (20%)	1 (20%)	1 (20%)
	Common cold	1 (20%)	0 (0%	0 (0%
	Subfebrile temperatures	1 (20%)	0 (%)	0 (%)
	Pneumonia	2 (40%)	1 (20%)	1 (20%)
Nervous system disorders				
	Anosmia	1 (20%)	0 (0%)	0 (0%)
	Loss of taste	1 (20%)	0 (0%)	0 (0%)
	Allodynia	1 (20%)	0 (0%)	0 (0%)
	Sleep disturbance	1 (20%)	0 (0%)	0 (0%)
	Fatigue	1 (20%)	0 (0%)	0 (0%)
	Subarachnoid bleeding (due to tumble)	1 (20%)	1 (20%)	1 (20%)
	Polyneuropathy	1 (20%)	0 (0%)	0 (0%)
	Headache	1 (20%)	0 (0%)	0 (0%)
	Bifrontal hygroma	1 (20%)	0 (0%)	0 (0%)
	Concentration disorder	1 (20%)	0 (0%)	0 (0%)
	Joint pain	1 (20%)	0 (0%)	0 (0%)
Gastrointestinal disorders				
	Mucositis	1 (20%)	0 (0%)	0 (0%)
	Vomiting	2 (40%)	0 (0%)	0 (0%)
	Diarrhea	1 (20%)	0 (0%)	0 (0%)
	Small gastric axial hernia	1 (20%)	0 (0%)	0 (0%)
Injury, poisoning, and procedural complications				
	Liquor Loss Syndrome	1 (20%)	0 (0%)	0 (0%)
Renal and urinary disorders				
	Increased creatinine level	1 (20%)	0 (0%)	0 (0%)
Blood and lymphatic system disorders				
	Decreased platelets	5 (100%)	3 (60%)	0 (0%)
	Decreased white blood count	4 (80%)	4 (80%)	0 (0%)
	Decreased absolute neutrophile count	4 (80%)	4 (80%)	0 (0%)
	Decreased lymphocytes	4 (80%)	4 (80%)	0 (0%)
	Anemia	2 (40%)	2 (40%)	0 (0%)
Investigations				
	Aspartate aminotransferase elevated	2 (40%)	0 (0%)	0 (0%)
	Alanine aminotransferase elevated	2 (40%)	0 (0%)	0 (0%)
	Gamma Glutamyltransferase elevated	1 (20%)	0 (0%)	0 (0%)
	Lactate dehydrogenase elevated	1 (20%)	0 (0%)	0 (0%)
	Bilirubin elevated	1 (20%)	0 (0%)	0 (0%)
	Alkaline phosphatase elevated	2 (40%)	0 (0%)	0 (0%)
	Immunoglobulin G level decreased	1 (20%)	1 (20%)	0 (0%)
General disorders and administration side conditions				
	Asthenia Pyrexia	1 (20%) 1 (20%)	1 (20%) 1 (20%)	1 (20%) 1 (20%)
Cardiac disorders				
	Tachyarrythmia absoluta	1 (20%)	1 (20%)	0 (0%)
Metabolism and nutrition disorders				
	Hypokalemia	3 (60%)	1 (20%)	0 (0%)
	Hypernatremia	1 (20%)	0 (0%)	0 (0%)
	Hyponatremia	2 (40%)	0 (0%)	0 (0%)
	Hypocalcemia	1 (20%)	0 (0%)	0 (0%)
	Hypalbuminemia	1 (20%)	0 (0%)	0 (0%)
Skin and subcutaneous tissue disorders				
	Erythema	2 (40%)	0 (0%)	0 (0%)
	Ulceration	1 (20%)	0 (0%)	0 (0%)
	Worsening of atopic dermatitis	1 (20%)	0 (0%)	0 (0%)
	Limb edema	2 (40%)	0 (0%)	0 (0%)
Embolic and thrombotic events				
	Lung embolism	1 (20%)	0 (0%)	0 (0%)

## Data Availability

The clinical trial protocol is attached as Supplementary Materials to this article and includes the statistical analysis plan. Primary individual participant data may be shared with researchers whose proposed use of the data has been approved by an independent review committee identified for this purpose upon request. All secondary data derived from primary individual participant data are available within the article, Supplementary Information, or Supplementary Data Files.

## Supplementary Materials

The following supporting information can be downloaded at: https://www.mdpi.com/article/10.3390/cancers18030455/s1. Supplemental Table S1: The pharmacokinetic results for venetoclax (plasma and CSF) and obinutuzumab (serum and CSF) for the individual patients at the respective time points of sampling, Supplemental Figure S1: Correlation of venetoclax and obinutuzumab concentration in cerebrospinal fluid to progression-free survival, PK-Sampling Manual Venetoclax, PK-Sampling Manual Obinutuzumab, Clinical Trial Protocol.

## Abbreviations

The following abbreviations are used in this manuscript:

1L	First Line
2L	Second Line
AE	Adverse Event
BCL-2	B-cell lymphoma 2
BCNU	Carmustine
BCRP	Breast Cancer Resistance Protein
BOIN	Bayesian Optimal Interval
BTK	Bruton’s Tyrosine–Kinase
Bu	Busulfan
CD	Cluster of Differentiation
CNS	Central Nervous System
CSF	Cerebrospinal Fluid
CTCAE	Common Terminology Criteria for Adverse Events
CR	Complete Remission
CTP	Clinical Trial Protocol
CYP3A	Cytochrome P3A
DLT	Dose-limiting Toxicity
ECOG	Eastern Cooperative Oncology Group
EDTA	Ethylenediaminetetraacetic acid
ELISA	Enzyme Linked Immunosorbent Assay
FAS	Full Analysis Set
FFS	Failure-free Survival
HCT-ASCT	High-dose Chemotherapy and Autologous Stem Cell Transplantation
HD-MTX	High-dose Methotrexate
HIV	Human Immunodeficiency Virus
IELSG	International Extranodal Lymphoma Study Group
IMP	Investigational Medicinal Product
IPCG	International PCNSL Collaborative Group
MARTA	High-dose (HD)-MTX, HD-Cytarabine (AraC), Rituximab
MedDRA SOC	Medical Dictionary for Regulatory Activities System Organ Class
mg	Milligram
ml	Milliliter
mTOR	Mammalian Target of Rapamycin
ng	Nanogram
LC-MS	Liquid chromatography–mass spectrometry
ORR	Overall Response Rate
OS	Overall Survival
Pat.ID	Patient Identification Number
pEOT	Premature end of treatment
PB	Peripheral Blood
PCNSL	Primary Central Nervous System Lymphoma
PD	Progressive disease
PFS	Progression-free survival
P-gp	P-glycoprotein
PK	Pharmacokinetics
PR	Partial Remission
PS	Performance Status
R	Rituximab
R-DeVIC	Rituximab, Dexamethasone, infusional Etoposide, Ifosfamide, and Carboplatin
RA	Response Assessment
RR	Relapsed/Refractory
SAE	Serious Adverse Event
SD	Standard Deviation or Stable Disease
SOC	Standard of care
TT	Thiotepa
