# Supplementary material for: The Phase Ib VenObi CNS Study: Chemotherapy-Free Treatment with Venetoclax and Obinutuzumab for Relapsed/Refractory Primary Large B-Cell Lymphoma of the Central Nervous System
Source: Cancers (Basel). 2026 Jan 30;18(3):455. doi: 10.3390/cancers18030455 (PMC12896398; doi:10.3390/cancers18030455)
Supplement: Supplementary file 1 [file cancers-18-00455-s001.zip › cancers-4089811-supplementary.pdf]

Supplemental Tables/Figures

Supplemental Table S1 Pharmacokinetic results for venetoclax (plasma and CSF) and obinutuzumab (serum and CSF) for the individual patient at the respective time points of sampling

Subject	Visit	Time point	Venetoclax[1]			Obinutuzumab[2]		
			Plasma* Concentration (ng/mL)	CSF* Concentration (ng/mL)	Ratio CSF*/ plasma (%)	Serum ^{X} Concentration (McG/mL)	CSF ^{X} Concentration (ng/mL)	Ratio CSF/ serum (%)
**01-01**	C1D1	Pre dose	0.00	n.a.	n.a.	<0.00405	n.a.	n.a.
		Post dose	355	n.a.	n.a.	303	n.a.	n.a.
	C1D3	Pre dose	465	2.58	0.55	200	79.6	0.40
	C1D15	Pre dose	1420	8.55	0.60	369	1370	0.37
	C2D7	Pre dose	732	6.03	0.82	555	2360	0.43
	Main	Pre dose	269	n.a.	n.a.	n.a.	n.a.	n.a.
**01-02**	C1D1	Pre dose	0.00	n.a.	n.a.	<0.00405	n.a.	n.a.
		Post dose	1950	n.a.	n.a.	518	n.a.	n.a.
	C1D3	Pre dose	502	1.03	0.21	455	45.6	0.01
	C1D15	Pre dose	470	2.51	0.53	586	1550	0.26

	C2D7	Pre dose	153	0.77	0.50	1280	1250	0.10
	Main	Pre dose	250	n.a.	n.a.	n.a.	n.a.	n.a.
**01-03**	C1D1	Pre dose	0.00	n.a.	n.a.	<0.00405	n.a.	n.a.
		Post dose	1550	n.a.	n.a.	n.d.	n.a.	n.a.
	C1D3	Pre dose	397	2.44	0.61	n.d.	n.d.	n.d.
	C1D15	Pre dose	n.d.	n.d.	n.d.	n.d.	n.d.	n.d.
	C2D7	Pre dose	1140	14.8	1.30	486	3870	0.80
	Main	Pre dose	n.d.	n.a.	n.a.	n.a.	n.a.	n.a.
**01-04**	C1D1	Pre dose	0.00	n.a.	n.a.	<0.00405	n.a.	n.a.
		Post dose	2060	n.a.	n.a.	245	n.a.	n.a.
	C1D3	Pre dose	285	1.80	0.63	239	32.9	0.14
	C1D15	Pre dose	3760	8.93	0.24	254	622	0.24
	C2D7	Pre dose	2290	6.98	0.30	417	1070	0.26
	Main	Pre dose	0.00	n.a.	n.a.	n.a.	n.a.	n.a.
**02-01**	C1D1	Pre dose	0.00	n.a.	n.a.	n.d.	n.d.	n.d.
		Post dose	174	n.a.	n.a.	n.d.	n.d.	n.d.

	C1D3	Pre dose	72.6	0.17	0.23	n.d.	n.d.	n.d.
	C1D15	Pre dose	190	1.13	0.59	n.d.	n.d.	n.d.
	C2D7	Pre dose	n.d.	n.d.	n.d.	n.d.	n.d.	n.d.
	Main	Pre dose	n.d.	n.a.	n.a.	n.d.	n.d.	n.d.

Peripheral blood (PB) samples were drawn on the following time points: day 1 (pre-dose and post-dose), days 3, 15, 28 (each pre-dose) and at the first maintenance visit (4 weeks after end of induction treatment, pre-dose) as well as cerebrospinal fluid (CSF) samples on days 3, 15 and 28 (all pre-dose). Scheduled samples that were not drawn are marked as not done (n.d.) and time points that were not scheduled for the respective sample as not applicable (n.a.)

Main = 1st maintenance phase visit, 4 weeks after end of induction treatment visit

* samples were stored for a maximum of 1057 days, and the existing stability data support long-term storage stability for 371 days for CSF samples and 1480 days for plasma sample

X samples were stored for a maximum of 1057 days, and the existing stability data support long-term storage stability for 767 days for CSF and 4264 days for serum samples.

1 the analyte venetoclax (A1195425) in Human Plasma K2 EDTA and CSF was performed using Liquid/Liquid Extraction followed by Liquid chromatography–mass spectrometry (LC-MS)/MS detection in a designated laboratory from AbbVie.

2 the analyte obinutuzumab (RO5072759/rhuMab anti-CD20) in Human Serum and CSF was analyzed using quantitative ELISA (Enzyme Linked Immunosorbent Assay). In this assay, calibration standards, QC samples, and study samples are pre-incubated with a cocktail containing biotin- and digoxigenin-labeled anti-idiotype antibodies directed to the antigen binding site of RO5072759. The pre-incubation mixture is then transferred to the appropriate wells of a streptavidin-coated microplate. Immune complexes bound to the microplate are detected by addition of horseradish peroxidase-conjugated Fab fragments directed to digoxigenin (Fab<DIG>-POD). ABTS substrate is used to produce a colorimetric signal proportional to the amount of RO5072759 captured by the biotinylated antigen. The color development is measured by reading at 405 for detection absorbance and 490 nm for reference absorbance.

S

Supplemental Figure S1 Correlation of venetoclax and obinutuzumab concentration in cerebrospinal fluid to progression-free survival

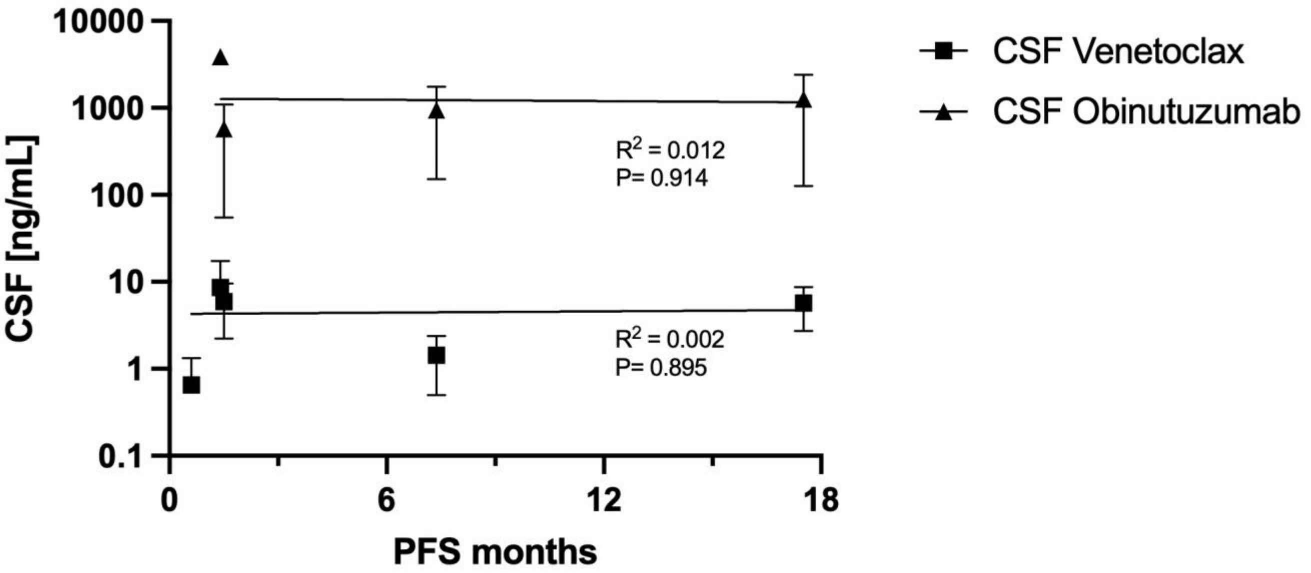

VENOBI PK-Sampling Venetoclax—VERSION 1.0 – 2020/03/10

Visit	Test	Collection Tubes	Instructions	Tubes to be sent	Shipping temperature	Shipping contact
**C1D1**<br>(pre+postdose)<br>(*30 minutes after infusion of obinutuzumab)<br><br>**C1D3**<br>(predose)<br><br>**C1D15**<br>(predose)<br><br>**C2D7**<br>(predose)<br><br>**MAINT** only once 4 weeks after EOITA	PK analyses<br>**Peripheral blood PB**	2,7 ml EDTA K_3 tube (Kalium-EDTA)	Whenever possible, take the blood PK samples at the same time when routine blood tests are taken to avoid unnecessary additional venous punctures<br><br>Prepare an ice bath<br><br>Take the blood sample by venous puncture<br><br>Immediately after collection, invert the blood sample gently (5-10 times) afterwards place it in the ice bath while preparing the centrifuge<br><br>Within 1 h of blood collection centrifuge the blood sample at 1500 x g for 10 minutes at 4 degrees Celsius<br><br>Pipet 1.2 ml of the supernatant into the 2 ml Greiner Cryo.s™ vial<br><br>Place the Greiner Cryo vial in a freezer (-70 degrees Celcius or colder) within 2 hours after collection until transferred to the AbbVie laboratory<br><br>The EDTA tube with remaining pellet can be discarded	2 ml Greiner Cryo.s™ vial<br>(1 vial per sample)	Frozen on dry ice	**Attn: AbbVie Sample Receiving**<br><br>Dept. R46W, Bldg. AP13A, Room 2310<br>c/o: Delivery Services<br>1150 S. Northpoint Blvd.<br>Waukegan, IL 60085<br>USA<br><br>Phone: (847) 937-0889<br>Fax: (847) 938-9898<br>Email: sample.receiving@abbvie.com

Pre-dose means either before obinutuzumab infusion and/or venetoclax intake on the respective day!

PB PK tubes from routine stock in center

VENOBI PK-Sampling Venetoclax—VERSION 1.0 – 2020/03/10

Visit	Test	Collection Tubes	Instructions	Tubes to be sent	Shipping temperature	Shipping contact
**C1D3**<br><br>**C1D15**<br><br>**C2D7**<br><br>All<br>Pre-dose	PK analyses<br>**Cerebrospinal fluid (CSF)**<br><br>**Venetoclax CSF sample must be processed first, because it does not require centrifugation!**	15 ml Sarstedt tube	Prepare an ice bath<br><br>Prepare the patient and conduct the lumbar puncture as per local guidelines and collect a total of 4ml CSF (including PK for Obinutuzumab) – it is very important that the sample is free of blood – and place it in an ice bath.<br><br>Dilute Tween 20 by a ratio of 1:10 with ultrapure grade water and mix thoroughly in a separate tube (in example: 1ml of Tween 20 and 9ml of ultrapure grade water)<br><br>Accurately pipet 0.015 ml of the solution into the Greiner Cryo.s™ vial<br>Securely cap the tube and invert several times to coat the tube<br><br>Transfer 1.5 ml of the CSF sample into the 2 ml Greiner Cryo.s™ vial (prepared with Tweed).<br><br>Place the Greiner Cryo vial in a freezer (-70 degrees Celcius or colder) within 2 hours after collection until transferred to the AbbVie laboratory<br><br>**Do not discard the remaining content, because needed for obinutuzumab sample!**	2 ml Greiner Cryo.s™ vial<br>(1 vial per sample)	Frozen on dry ice	Attn: AbbVie Sample Receiving<br><br>Dept. R46W, Bldg. AP13A, Room 2310<br>c/o: Delivery Services<br>1150 S. Northpoint Blvd.<br>Waukegan, IL 60085<br>USA<br><br>Phone: (847) 937-0889<br>Fax: (847) 938-9898<br>Email: sample.receiving@abbvie.com

Pre-dose means either before obinutuzumab infusion and/or venetoclax intake on the respective day!

CSF PK tubes from routine stock in center

VENOBI PK-Sampling Obinutuzumab, VERSION 1.0 – 2020/03/10

Visit	Test	Collection Tubes	Instructions	Tubes to be sent	Shipping temperature	Shipping contact
**C1D1** pre+postdose* (*30 minutes after infusion)<br><br>**C1D3** (predose)<br><br>**C1D15** (predose)<br><br>**C2D7** (predose)<br><br>**MAINT** only once 4 weeks after EOITA	PK analyses **Peripheral blood (PB)**	7.5 ml serum tube (S-Monovette)	Whenever possible, take the blood PK samples at the same time when routine blood tests are taken to avoid unnecessary additional venous punctures<br><br>Take the blood sample by venous puncture<br><br>Immediately after collection, invert the blood sample gently (5-10 times)<br><br>Put the tube into an upright position and allow the blood to clot for about 30 minutes at room temperature<br><br>Within 1 hour of blood collection centrifuge the blood sample at 1500 x g for 10 minutes at 4 degrees Celsius<br><br>Pipet 1.2 ml of the supernatant into the 2 ml Greiner Cryo.s™ vial<br><br>The serum tube with remaining clotted blood can be discarded<br><br>Transfer the Greiner Cryo vial in a freezer (-20 degrees Celcius or colder) within 2 hours after collection until transferred to the PPD laboratory	2 ml Greiner Cryo.s™ vial (1 vial per sample)	Frozen on dry ice	Contact: Attn: Specimen Management / LiMajor Pittman<br>Megan Wiberg, megan.wiberg@ppdi.com<br>Phone +1 804.977.8017<br>richmond_data@ppdi.com<br>RichmondSMOpeners@ppdi.com<br><br>Address: PPD<br>2246 Dabney Road<br>Richmond, VA 23230<br>USA

Pre-dose means <u>either before obinutuzumab infusion and/or venetoclax intake</u> on the respective day!

PB PK tubes from routine stock in center

VENOBI PK-Sampling Obinutuzumab, VERSION 1.0 – 2020/03/10

Visit	Test	Collection Tubes	Instructions	Tubes to be sent	Shipping temperature	Shipping contact
**C1D3**<br><br>**C1D15**<br><br>**C2D7**<br><br>Always pre-dose	PK analyses **Cerebrospinal fluid (CSF)**	15 ml Sarstedt tube	Prepare an ice bath<br><br>Prepare the patient and conduct the lumbar puncture as per local guidelines and collect <u>4ml CSF (including PK for Venetoclax)</u> – it is very important that the sample is free of blood - and place it in an ice bath.<br><br><u>After</u> you have processed the sample for Venetoclax PK, centrifuge the CSF sample at 2000 x g for 10 minutes at 4 degrees Celsius<br><br>Pipet 1.5 ml of the supernatant into the 2 ml Greiner Cryo.s™ vial<br><br>Transfer the Greiner Cryo vial in a freezer (-70 degrees Celcius or colder) within 2 hours after collection until transferred to the PPD laboratory<br><br>Remaining content in the Sarstedt tube can now be discarded.	2 ml Greiner Cryo.s™ vial (1 vial per sample)	Frozen on dry ice	Contact: Attn: Specimen Management / LiMajor Pittman<br>Megan Wiberg, megan.wiberg@ppdi.com<br>Phone +1 804.977.8017<br>richmond_data@ppdi.com<br>RichmondSMOpeners@ppdi.com<br><br>Address: PPD<br>2246 Dabney Road<br>Richmond, VA 23230<br>USA

Pre-dose means <u>either before obinutuzumab infusion and/or venetoclax intake</u> on the respective day!

CSF PK tubes from routine stock in center

Klinikum Stuttgart

Clinical Trial Protocol

Chemotherapy free treatment with venetoclax and obinutuzumab for relapsed / refractory primary CNS lymphoma patients (VENOBI-CNS study) – A phase IB study to assess the pharmacokinetics in the cerebrospinal fluid

V3.0 / 2020-07-02 replaces Version 2.0 / 2019-10-29 replaces Version 1.0 / 2019-05-16

VENOBI-CNS

Primary central nervous system lymphoma (PCNSL)

**EudraCT No.**	2017-003690-33
**DRKS -No.**	DRKS00017615
**Internal Protocol ID No.**	ML40029
**NCT-No.**	NCT04073147
**Protocol Version**	V 3.0 / 2020-07-02
**Revision chronology, if applicable**	replaces V 2.0/2019-10-29 replaces V 1.0/2019-05-16
**Development Phase**	Phase IB
**Sponsor**	Klinikum der Landeshauptstadt Stuttgart gKAö<br>represented by the management board<br>Kriegsbergstraße 60<br>70174 Stuttgart, GERMANY
**Coordinating Investigator**<br>"Leiter der Klinischen Prüfung/LKP" (in accordance with German Drug Law)	Prof. Dr. G. Illerhaus<br>Klinikum Stuttgart<br>Kriegsbergstraße 60<br>70174 Stuttgart, GERMANY

This Clinical Trial Protocol contains confidential information. Circulation of this material to individuals who are not involved in the carrying out of the study or any kind of publication requires the approval of the sponsor. These limitations similarly relate to all confidential information and data which will be obtained in the future.

Approval of the Clinical Trial Protocol

Chemotherapy free treatment with venetoclax and obinutuzumab for relapsed / refractory primary CNS lymphoma patients (VENOBI-CNS study) – A phase IB study to assess the pharmacokinetics in the cerebrospinal fluid

**EudraCT No.:**	2017-003690-33
**Protocol Version No:**	V 3.0 / 2020-07-02

Coordinating Investigator<br>Leiter der Klinischen Prüfung / LKP<br>(in accordance with German Drug Law)	3.7.2020	
Prof. Dr. G. Illerhaus	Date	Signature
Clinical Methodologist	3.7.2020	
PD Dr. med. Benjamin Kasenda	Date	Signature

Investigator Statement

**Protocol Short Title:**	VENOBI-CNS
**EudraCT No.:**	2017-003690-33
**Protocol Version No:**	V 3.0 / 2020-07-02
**Trial Site:**	[Please enter particulars of the Trial Site]

I confirm that I have read the Clinical Trial Protocol (CTP) and hereby commit to adhering to all actions and terms as specified in the relevant sections of the clinical, ethical and general paragraphs.

I confirm that I and my colleagues will comply with the local legislation (in Germany, the German Drug Law with the appropriate amendments). I further confirm that the clinical trial will be carried out in compliance with the Declaration of Helsinki and ICH-GCP guidelines.

I acknowledge that all confidential information contained in this document will not be used for any other purpose other than the evaluation or conduct of the clinical investigation without the prior written consent of the Sponsor.

Under my supervision I put copies of this CTP and possible updates as well as access to all information regarding the carrying out of this clinical trial at the disposal of my colleagues; in particular I will promptly forward all information from the Sponsor in relation to pharmaceutical safety (SUSARs, SmPC and IB updates, if applicable) to my colleagues.

I confirm that I and my colleagues were informed by a responsible scientist about the results and expected risks of the pharmacological and toxicological examination associated with the clinical trial.

I will discuss this CTP in detail with the members of the study site team and ensure that they are comprehensively informed about the trial compound/preparation and the execution of the trial.

I confirm that I will be responsible for supervising any individual or party to whom I delegate study tasks conducted at the trial site.

Furthermore I commit myself not to commence patient enrolment prior to approval of the competent authorities (CA) and acceptance by the responsible Independent Ethics Committee (IEC).

Date	Name (in CAPITALS)	Signature of Investigator

Table of Contents

List of Figures **9**
List of Tables **9**
List of Abbreviations **10**
Synopsis **12**
Responsibilities **22**
1 Background and rationale **24**
1.1 Scientific background 24
1.2 Overview of investigational medicinal products (IMPs) 24
1.3 Trial purpose and rationale 25
1.4 Rational for choice of control interventions/comparators 26
1.5 Rational for dose selection 26
1.6 Risk-benefit assessment 26
2 Objectives and endpoints **27**
2.1 Primary objective 27
2.2 Secondary objectives 28
2.3 Tertiary objectives 28
2.4 Safety objectives 28
2.5 Primary objective and endpoint 28
2.6 Secondary objectives and endpoints 28
2.7 Tertiary objectives and endpoints 29
2.8 Definitions 29
2.8.1 Definition of dose limiting toxicity (DLT) 29
2.8.2 Definition of minimum safety evaluation requirements 30
3 Clinical trial plan **30**
3.1 Trial design 31
3.1.1 Dose limiting toxicities (DLTs) and venetoclax dose escalation 31
3.2 Treatment arms 32
3.3 Treatment duration 33
3.4 Number of patients 33
3.5 Participating sites 33
3.6 Recruitment rate 33
3.7 Translational studies 33
3.8 Trial timetable 33
4 Trial population and selection criteria **34**
4.1 Target population 34
4.1.1 Health condition studied 34
4.1.2 Gender distribution 34
4.2 Inclusion criteria 34
4.3 Exclusion criteria 35
5 Enrolment and patient registration **36**
5.1 Patient eligibility 36
5.2 Patient registration 36

5.3 Randomisation methodology 37
6 Treatment plan and procedure 37
6.1 Dosing regimen and IMP administration 37
6.1.1 Venetoclax 37
6.1.2 Obinutuzumab 37
6.2 Dose modification and dose delay / or dose reduction 38
6.2.1 Dose modification of Venetoclax - Haematological toxicity 38
6.2.2 Dose modification of Venetoclax - Non-haematological toxicity 38
6.2.3 Dose modification of Obinutuzumab - Haematological toxicity 39
6.2.4 Dose modification of Obinutuzumab – Non-haematological toxicity 39
6.3 Dosing groups of venetoclax 39
6.4 Concomitant treatment/medication 40
6.4.1 Permitted prior/concomitant treatment/medication 40
6.4.2 Rescue medications, non-drug therapies or supportive care 40
6.4.3 Prohibited and permitted concomitant therapy requiring caution and/or action40
6.5 Unblinding of treatment assignment 41
6.5.1 Premature unblinding 41
6.5.2 Unblinding procedure and documentation 41
6.5.3 Consequences for the patient's treatment 41
6.6 Treatment after end of the trial 41
7 Visit schedule and assessments 41
7.1 Flow and visit schedule 41
7.2 Visit and assessment windows 41
7.3 Screening and registration 42
7.3.1 Screening 42
7.3.2 Data to be collected on screening failures 42
7.3.3 Assessments at screening 42
7.3.4 Registration 43
7.4 Treatment 43
7.4.1 Assessments at Cycle 1 Day 1 (C1D1) 44
7.4.2 Assessments at Cycle 1 Day 3 (C1D3) 44
7.4.3 Assessments at Cycle 1 Day 8 (C1D8) 44
7.4.4 Assessments at Cycle 1 Day 15 (C1D15) 44
7.4.5 Assessments at Cycle 2 Day 1 (C2D1, +/- 3 days) 45
7.4.6 Assessments at Cycle 2 Day 7 (C2D7) 45
7.4.7 Assessments at Cycle 3 Day 1 (C3D1, +/- 3 days) 45
7.4.8 Assessments at Cycle 4 Day 1 (C4D1, +/- 3 days) 45
7.4.9 Assessments at Cycle 5 Day 1 (C5D1, +/- 3 days) 46
7.4.10 Assessments at Cycle 6 Day 1 (C6D1, +/- 3 days) 46
7.4.11 Assessment at end of induction treatment assessment (EOITA) (Day 127, +/- 3 days, end of induction treatment visit) 46

7.4.12 Assessments at Visits during maintenance treatment (once 4 weeks after end of induction treatment assessment (EOITA), thereafter every 8 weeks for 12 months counted from the first maintenance visit, +/- 5 days) 47
7.4.13 Assessments during Follow-Up 47
7.4.14 Assessments at End of study visit (EOSV) 47
7.4.15 Assessments if treatment or planned follow-up is discontinued prematurely.. 48
7.4.16 Routinely collected data after EOSV 48
7.5 Assessments and specifications 48
7.5.1 Patient demographics 48
7.5.2 Medical history 48
7.5.3 Pregnancy test and contraception 48
7.5.4 Physical examination 49
7.5.5 Vital signs 49
7.5.6 Height and weight 49
7.5.7 Performance status 50
7.5.8 Laboratory tests 50
7.5.9 Electrocardiogram (ECG) 50
7.5.10 Imaging, MRI brain 50
7.5.11 DLT decision and CTU notification 50
7.5.12 Imaging, CT body or PET/CT 51
7.6 Additional biological specimen collection for translational program 51
7.6.1 Pharmacokinetics (PK) 51
7.6.2 Collection of archival tissue for mutational analyses 51
8 Discontinuation criteria 52
8.1 Premature termination the entire trial 52
8.1.1 General note 52
8.1.2 Specific note for this study 52
8.2 Premature termination of the trial at one of the trial sites 52
8.3 Discontinuation of trial treatment or trial participation for individual patients 53
8.3.1 Premature discontinuation of trial treatment 53
8.3.2 Premature termination of trial participation 54
9 Investigational medicinal products (IMPs) 54
9.1 Obinutuzumab background information 54
9.1.1 Preclinical data 54
9.1.2 Pharmacokinetics 54
9.1.3 Pharmacodynamics 55
9.1.4 Adverse reactions 55
9.2 Venetoclax background information 55
9.2.1 Preclinical data 55
9.2.2 Pharmacokinetics 55
9.2.3 Pharmacodynamics 55

9.2.4 Adverse reactions 56
9.3 IMP(s) pharmaceutical characteristics 56
9.4 Packaging and labelling 56
9.5 Supply and ordering 56
9.6 Receipt and storage 56
9.7 Preparation of Obinutuzumab solution 57
9.8 Dispensing 57
9.9 Return and destruction 57
9.10 Drug compliance and accountability 57
9.11 Treatment adherence 58
10 Safety monitoring and reporting 58
10.1 Adverse Events (AEs) 58
10.1.1 Definition of AEs 58
10.1.2 AEs of special interest (AESI) 59
10.1.3 Documentation of AEs 60
10.2 Serious Adverse Events (SAEs) 60
10.2.1 Definition of SAEs 60
10.2.2 Documentation of SAEs 61
10.2.3 Investigator reporting requirements 61
10.2.4 Sponsor reporting requirements 62
10.2.5 Reviewing of SAEs 63
10.2.6 Case transmission verification of single case reports 64
10.2.7 Pregnancies 64
10.2.8 Opening of next dosing cohort 64
11 Data handling and data management 64
11.1 Data confidentiality 64
11.2 Documentation of trial data 65
11.2.1 Documentation in medical records 65
11.2.2 Documentation in CRF 65
11.3 Data management 65
11.4 Data coding 66
12 Quality assurance 66
12.1 Monitoring procedure 66
12.2 Source data verification (SDV) 67
12.3 Auditing procedures and inspections 67
13 Statistical planning and analysis 67
13.1 Trial design 67
13.2 Objectives and endpoints 67
13.3 Sample size calculation 67
13.4 Definition of populations included in the analyses 68
13.5 Methods of analysis 68
13.5.1 Patient demographics/other baseline characteristics 68

13.5.2 Trial medication 68
13.5.3 Concomitant medication 68
13.5.4 Primary endpoint 69
13.5.5 Secondary endpoints for efficacy 69
13.5.6 Safety parameters 69
13.6 Safety analyses (DLT appraisal) 70
13.7 Exploratory analyses on gene alterations and response 70
13.8 Timepoints of comprehensive analyses for efficacy and safety 70
14 Scientific steering and data monitoring committees 71
14.1 Scientific steering committee (SSC) 71
14.2 Data monitoring committee (DMC) 71
15 Ethical and legal principles 71
15.1 Regulatory and ethical compliance 71
15.2 Responsibilities of the investigator 71
15.3 Informed consent procedures 72
15.4 Patient insurance 72
15.5 Confidentiality of trial documents and patient records 73
15.6 Financial disclosure 73
16 Trial documents and archiving 73
16.1 Trial documents/investigator site file 73
16.2 Archiving 74
16.3 Access to trial data 74
17 Protocol adherence and amendments 74
17.1 Protocol adherence 74
17.2 Amendments to the protocol 74
18 Administrative Agreements 75
18.1 Financing of the trial and role of funders 75
18.2 Trial agreement- investigator compensation 75
18.3 Reimbursement of trial patients 75
18.4 Trial reports 75
18.5 Clinical trials registry 75
18.6 Publication of trial protocol and results 75
18.7 Authorship in publications of trial protocol and results 76
19 References 77
19.1 Relevant Guidelines and Laws 81

List of Figures

FIGURE 1: Trial design 30
FIGURE 2: Dosing groups 32

List of Tables

TABLE 1: Visit schedule and assessments – Flowchart 19
TABLE 2: Rules for venetoclax dose escalation and de-escalation following the BOIN design. ..32
TABLE 3: Dose modification of Venetoclax for haematological toxicity 38
TABLE 4: Dose modification of Venetoclax for non-haematological toxicity 38
TABLE 5: Dose modification of Obinutuzumab for haematological toxicity 39
TABLE 6: Dose modification of Obinutuzumab for non-haematological toxicity 39
TABLE 7: Intra-patient dosing groups for venetoclax when re-starting after discontinuation for toxicity. 39

List of Abbreviations

Abbreviation	Full Name
AE	Adverse event
ALAT	Alanine Aminotransferase
ANC	Absolute neutrophil count
ASAT	Aspartate Aminotransferase
BCRP	Breast Cancer Resistance Protein
BOIN	Bayesian Optimal Interval
CA	Competent authorities
CD	Cluster of Differentiation
CI	Confidence interval
CIOMS	Council for international organizations of medical science
CLL	Chronic lymphocytic leukaemia
CMO	Chief Medical Officer
CNS	Central Nervous System
CR	Complete Remission
CRA	Clinical research associate
CRF	Case report form
CSF	Cerebrospinal fluid
CT	Computed tomography
CTCAE	Common terminology criteria of adverse events
CTP	Clinical Trial Protocol
CTU	Clinical trial unit
DLBCL	Diffuse large B cell lymphoma
DLT	Dose limiting toxicity
DRKS	Deutsches Register Klinischer Studien
ECOG	Eastern Cooperative Oncology Group
EOITA	End of induction treatment assessment
EOSV	End of study visit
FDA	Food and drug administration
FFS	Failure free survival
FMI	Foundation Medicine
FPI	First patient in
Gamma-GT	Gamma-glutamyl transferase
GCP	Good Clinical Practise
HCT-ASCT	High-dose chemotherapy with autologous stem cell support
HD-AraC	High-dose cytarabine
HD-MTX	High-dose methotrexate
HIV	Human immunodeficiency virus
IB	Investigator’s Brochure
ICF	Informed consent form
ICH-GCP	International conference on harmonization and good clinical practise
IEC	Independent Ethics Committee
IELSG	International extranodal lymphoma study group
IMP	Investigational medicinal product
INR	International normalized ratio
IPCG	International Primary CNS Lymphoma Group
ISF	Investigator Site File
LDH	Lactate dehydrogenase
LPI	Last patient in
LPLV	Last patient last visit

MAINT	Maintenance
MedDRA	Medical Dictionary for Regulatory Activities
MRI	Magnet resonance imaging
MTX	Methotrexate
NHL	Non-Hodgkin Lymphoma
ORR	Overall response rate
OS	Overall survival
PB	Peripheral blood
PCNSL	Primary central nervous system lymphoma
PD	Progressive Disease
PET	Positron Emission Tomography
PFS	Progression free survival
PHI	Protected health information
PK	Pharmacokinetics
PR	Partial Remission
PT	Preferred term, MedDRA
PTT	Partial Thromboplastin Time
SAE	Severe adverse event
SD	Stable Disease
SDEA	Safety data exchange agreement
SmPC	Summary of Product Characteristics (Fachinformation)
SOC	System organ class, MedDRA
SPC	Summary of product characteristics
SUSAR	Suspected Unexpected Serious Adverse Reaction
TLS	Tumour lysis syndrome
TMF	Trial Master File
ULN	Upper limit of Normal
WCBP	Women of childbearing potential

Synopsis

**TITLE OF TRIAL**	Chemotherapy free treatment with venetoclax and obinutuzumab for relapsed / refractory primary CNS lymphoma patients (VENOBI-CNS study) – A phase IB study to assess the pharmacokinetics in the cerebrospinal fluid
**SHORT TITLE**	VENOBI-CNS
**EUDRACT NO**	2017-003690-33
**PROTOCOL NUMBER/INTERNAL PROTOCOL NUMBER**	Protocol Number: V3.0 / 2020-07-02 replaces Version 2.0 / 2019-10-29 replaces Version 1.0 / 2019-05-16<br>Internal Protocol Number: ML40029
**HEALTH CONDITION STUDIED**	Primary central nervous system lymphoma (PCNSL)
**PHASE**	Phase IB
**BACKGROUND**	There is no standard treatment for relapsed or refractory PCNSL and strategies highly depend on previous treatments and clinical performance status.<br>The most common genetic imbalance in PCNSL are gains of 18q21, which includes the BCL2 locus. Furthermore, the IELSG32 trial has established anti-CD20 directed therapy with rituximab in PCNSL. Obinutuzumab has additional pharmacodynamics features compared to rituximab, therefore it is consequent to test it in relapsed or refractory PCNSL as well. Based on data available, there are no additional concerning safety issues when combining venetoclax with obinutuzumab, of note, there is no evidence for higher risk of side effects with increased venetoclax doses. Therefore, we propose a single arm multicentre phase IB dose-escalation trial to investigate a chemotherapy free treatment of venetoclax in combination with fixed-dose obinutuzumab in relapsed or refractory immunocompetent PCNSL patients.
**OBJECTIVE(S)**	<u>Primary objective</u><br>• To investigate the pharmacokinetics (PK) of venetoclax and obinutuzumab in the cerebrospinal fluid (CSF) in patients with relapsed or refractory PCNSL<br><br><u>Secondary objectives</u><br>• To investigate preliminary clinical efficacy such as lymphoma response and event-free survival (We will use the standardized IPCG response criteria (1) to categorize the lymphoma response.)<br>• Dose limiting toxicities (DLTs) during escalation of dosing groups as defined by CTCAE (version 5.0) and recommended phase II dose<br><br><u>Tertiary objectives</u><br>• To investigate frequency of gene alterations in DNA extracted from lymphoma tissue using the FoundationOneHeme platform.<br><br><u>Safety objectives</u><br>• To investigate safety and tolerability (dose limiting toxicity [DLT]) of venetoclax and obinutuzumab at different dosing levels

**TREATMENT**	All patients are planned to receive six cycles of induction treatment (combination of obinutuzumab and venetoclax, 18 weeks [six 3 weekly cycles]) and if at least stable disease without clinical deterioration has been achieved, patients will go on to maintenance treatment for 12 months (52 weeks) with venetoclax.<br><br>During the combination induction phase, obinutuzumab at 1000mg will be given for 6 cycles (day 1, 8 & 15 [first cycle] and on day 1 in cycles 2 till 6; cycles repeated every 21 days) together with daily venetoclax (600mg [N=5], 800mg [N=5] or 1000mg [N=5]). Assignment to the respective dosing groups will be consecutively, starting at 600mg.<br><br>Dose escalation will follow pre-specified rules according to the BOIN design. In brief: Only if zero or only 1 dose limiting toxicity (DLT) occurs in 5 patients and all patients fulfilled minimum safety evaluation requirements, the next cohort will be opened as planned. Patient recruitment will be stopped and not resumed until the safety assessment for the DLTs have been completed<br><br>To assess lymphoma response, gadolinium enhanced MRI brain, as per local standards, has to be done at screening, on day 22, day 43, day 85, and at the end of induction treatment (day 127). During maintenance treatment, MRI brain is conducted once after 4 weeks; thereafter every 8 weeks for the first 6 months and every 12 weeks thereafter.<br><br>In case of disease progression or unacceptable toxicities, treatment will be stopped.<br><br>Minimal follow-up per patient is 3 months after completing maintenance treatment.
**INCLUSION CRITERIA**	Inclusion criteria:<br>1. Age at inclusion ≥ 18 to 80 years, in case of ECOG 0 to 1 age up to 85 years<br>2. Eastern Cooperative Group performance status (ECOG) ≤ 3<br>3. Evaluable lymphoma manifestation in the CNS, either contrast-enhanced lesion in the brain parenchyma or measurable meningeal lesions.<br>4. Biopsy proven CD20 positive PCNSL at initial diagnosis or previous relapse/progression (re-biopsy at study inclusion is not mandatory for inclusion, but strongly recommended if time in remission is longer than 24 months).<br>5. At least one prior HD-MTX containing chemotherapy application (MTX dosed at ≥ 1 g/m^2 body surface area) before progression or relapse.<br>6. Confirmed relapsed or refractory PCNSL according to the IPCG response criteria with the following definition: Evidence of disease recurrence following PR/CR or uCR or no radiological response (SD or PD) as per the IPCG criteria to prior chemotherapy regimen(s), at least one of them containing high-dose methotrexate.<br>7. Absolute neutrophil count (ANC) of at least 1’500/µl<br>8. Platelet count of at least 50’000/µl<br>9. Adequate liver (alanine aminotransferase [ALAT] and AST ≤ 3.0 x upper limit of normal [ULN] and total bilirubin ≤ 1.5 x ULN) and kidney function (estimated ≥ 30ml/min creatinine clearance according to Cockgroft-Gault formula)<br>10. Written informed consent<br>11. Recovery from toxicity from previous anti-lymphoma treatment to ≤ grade 2

**EXCLUSION CRITERIA**	Exclusion criteria:<br>1. Known allergy to venetoclax or other components of the formulation<br>2. Known allergy to obinutuzumab or other components of the formulation<br>3. Primary ocular lymphomas *without* brain parenchymal involvement<br>4. Lymphoma relapse outside the CNS; extra CNS relapse needs to be ruled out by body CT scans (neck till pelvis) or PET-CT scans.<br>5. Contraindications for lumbar puncture at the discretion of the clinical investigator<br>6. Prior exposure to obinutuzumab or venetoclax<br>7. Other additional anti-lymphoma treatment, e.g. chemotherapy or radiotherapy<br>8. Active hepatitis B or C<br>9. HIV seropositivity<br>10. Chronic use of immunosuppressive drugs, e.g. steroids for systemic autoimmune disease<br>11. Active infections requiring treatment<br>12. Other active malignancies (except non-melanoma skin cancer). Prior malignancies without evidence of disease for at least 5 years are allowed<br>13. Patient is pregnant or breastfeeding, or expecting to conceive or father children within one year of finishing venetoclax and 18 months for obinutuzumab.<br>14. Prior allogeneic haematopoietic stem cell or solid organ transplantation<br>15. Therapeutic intervention in setting of other former interventional clinical trial within 30 days before the first IMP administration in VENOBI study; simultaneous participation in registry and diagnostic studies or follow up of an interventional trial is allowed<br>16. Patient without legal capacity who is unable to understand the nature, significance and consequences of the trial<br>17. Known or persistent abuse of medication, drugs or alcohol<br>18. Person who is in a relationship of dependence/employment with the sponsor or the investigator<br>19. Administration of moderate or strong CYP3A inhibitors or inducers within 1 week of initiation of venetoclax dosing.

**ENDPOINTS**	Primary endpoint<br>• PK of venetoclax and obinutuzumab expressed by the respective serum concentration and CSF concentration (µg/ml) measured within the first month since start of treatment on day 3, 15, and 28. We will calculate the ratio of the respective concentrations to investigate penetration into the CNS compartment. AUC will be calculated to express concentrations over time.<br>Key secondary and tertiary endpoint(s):<br>• Dose limiting toxicities (DLTs) during escalation of dosing groups within the first 6 weeks as defined by CTCAE (version 5.0) and recommended phase II dose level. DLT is defined as:<br>- Trial therapy related death<br>- Grade 4 neutropenia not resolved after 14 days despite growth factor support<br>- Grade 3 to 4 febrile neutropenia<br>- Grade 4 thrombocytopenia not resolved after 14 days<br>- Grade 2 or higher bleeding associated with thrombocytopenia<br>- Any other grade 3 or higher haematological or non-haematological adverse event related to one or both IMPs that does not resolve to at least grade 2 or to baseline value within 3 weeks since onset by complete drug discontinuation and supportive care if applicable *except*:<br>o Alopecia<br>o Nausea and diarrhoea adequately treated<br>• Best lymphoma response achieved during induction (CR, PR, SD or PD).<br>• Progression-free survival 1 (PFS1), which is defined as the time from the date of first dose until date of progression, relapse or death, whichever occurs first. Progression or relapse of the lymphoma will be evaluated by brain MRI as per schedule of assessment. In case of clinical suspicion of progression or relapse, brain MRI can be done as clinically indicated.<br>• OS; this will be calculated from the date of first dose until death due to any cause.<br>• Progression-free survival 2 (PFS2), which is defined as the time from the start of maintenance venetoclax treatment at week 12 until date of progression, relapse or death, whichever occurs first. Patients not reaching week 12 will be excluded from analysis of this endpoint.<br>• Distribution and frequency (proportion) in which FoundationOne Heme® One analyses based on formalin fixed tissue identified genetic alterations in the lymphoma cells expressed as allelic frequency in total and stratified by responding and non-responding patients.<br>Assessment of safety:<br>• Adverse events and serious adverse events grading evaluated by CTCAE (version 5.0).

**TRANSLATIONAL STUDIES**	The objective of FMI testing (FoundationOne Heme® One platform, tertiary objectives) is to gain more insights into the mutational landscape of PCNSL and to investigate matched samples from lymphoma tissue (formalin-fixed), CSF, and peripheral blood (intra-patient validity) over time; an approach that has currently not been conducted in the field of PCNSL research. There will be no repeat brain biopsy unless clinically indicated. All samples will be pseudonymized. Results of analyses will be disclosed to the treating physician in a standardized report (BioPharma CLIA) as soon as each report from each individual sample has been issued. The treating physician will disclose results from FMI testing to the patient at latest at the time of lymphoma progression. At that time point, results from FMI testing may positively support further treatment decision making. However, if the patient asks for the results before lymphoma progression, the treating physician will have to disclose the results. In that case, it should be emphasized that the results should not alter the treatment plan if the patient tolerates current treatment and shows lymphoma response. This is because it is yet unclear to what extend FMI testing really helps to positively alter patient management.
**TRIAL DESIGN**	This is a non-randomized single arm open label dose-escalating (600mg, 800mg and 1000mg venetoclax always combined with 1000mg obinutuzumab) phase IB trial conducted at two German sites.
**STATISTICAL ANALYSIS**	15 patients will be enrolled into this study to investigate the PK of venetoclax and obinutuzumab (five for each dosing group). In case a patient is not assessable during DLT time (during the first two cycles) for whatever reason (e.g. death definitely unrelated to study drug, withdrawal of consent, lost to follow-up), we will replace the patient to guarantee that there are always five patients assessable for DLT in each of the three dosing groups. To assure patient's safety, we will follow the BOIN (Bayesian optimal interval) design to determine feasibility for dose escalation or de-escalation (2). For this, we set the target DLT at 30%. Further details on decision boundaries are described in the protocol.<br><br>For the primary endpoint PK, all patients with at least one paired sample (CSF and peripheral blood [PB]) will be considered for analysis.<br><br>We will describe the concentration of venetoclax and obinutuzumab in each patient in the serum and CSF at the respective time points (as outlined above). The median (range, interquartile range) of the concentration based on all evaluable patients at the respective time points will be computed separately for serum and CSF. To describe the CSF penetration of the respective compound, we will calculate the ratio of the CSF concentration / serum concentration.<br><br>Description of the primary analysis and population:<br><br>For the primary endpoint PK, all patients with at least one paired sample (CSF and PB) will be considered for analysis.<br><br>We will conduct two analyses for efficacy. One is based on all registered patients in the denominator, irrespective whether treatment was applied, irrespective whether they refused or discontinued the treatment or whether other protocol violations are revealed (intention-to-treat principle). Second analysis will be based on all patients who have received at least one treatment application for sensitivity analyses (as treated analysis). Safety analyses will be performed in the safety population which includes all patients who have received at least one dose of trial treatment.

Safety:
The total number of AEs, the minimum, maximum and mean number of AEs per patient, the total number of follow-up days (number of days in the observation period), the number of AEs per follow-up day (total number of AEs divided by the total number of follow-up days), the number of patients who had at least one AE, and the number of patients who stopped treatment due to AE will be given. Same will be done for SAEs. We will use the CTCAE grading system to describe severity of any AE or SAE.

Secondary endpoints:
For all secondary endpoints, we will primarily present all data based on the 15 patients, but will also provide exploratory stratified analyses by dosing group. Patients with missing data regarding lymphoma response will be considered as non-responders. For time to event data (PFS, FFS, and OS), patients will be censored at last date of follow-up if they did not experience the event of interest beforehand. We will calculate respective survival probabilities at the 6, 9, and 12 months landmarks accompanied with 95% confidence intervals (CIs). Median survival times with 95% CIs will be calculated if reached. Again, because this study is entirely exploratory in nature, there will be no tests for hypothesis testing.

Safety analyses (DLT appraisal):

There will be two safety assessments for DLTs after the first cohort before preceding to cohort 2 and another one before preceding to cohort 3. DLT assessment will always take place 6 weeks after first drug application to the 5th patient of the respective cohort. The following rules based on the BOIN approach (2) (see 3.1 [Trial design]) apply to the safety analyses regarding DLTs (see 2.8.1 [Definition of dose limiting toxicities]):

**Rule**	**Target number of patients treated at each dose level (dose level of venetoclax)**		
	**N=5** (600mg)	**N=5** (800mg)	**N=5** (1000mg)
Escalate if number of DLT is smaller or equal to:	1	1	1
De-escalate if number of DLT is larger or equal to:	2	2	2
Terminate study if number of DLT is larger or equal to:	4	4	4

**SAMPLE SIZE**	To be assessed for eligibility:	n = 20
	To be registered to trial:	n = 15
	To be analysed:	n = 15

	In case a patient is not assessable during DLT time (during the first two cycles) for whatever reason (e.g. death definitely unrelated to study drug, withdrawal of consent, lost to follow-up), we will replace the patient to guarantee that there are always five patients assessable for DLT in each of the three dosing groups.	
**TRIAL DURATION**	Recruitment period (months):	24 months
	First patient in to last patient out (months):	41 months
	Treatment duration per patient: 70 weeks (~16 months) (18 weeks induction, 52 weeks maintenance) or until disease progression or toxicities	16 months
	Follow up duration per patient (months) after completion maintenance treatment:	3 months
**PLANNED DATES**	Enrolment of first patient, first patient in (FPI)	2nd quarter 2020
	Enrolment of last patient, last patient in (LPI)	2nd quarter 2022
	End of trial defined as last patient last visit (LPLV)	4th quarter 2023
	Final analysis and study report	3rd quarter 2024
	Planned interim analysis	NA
**PARTICIPATING SITES**	2 sites are planned in Germany (Stuttgart and Freiburg).	
**SPONSOR**	Klinikum Stuttgart, Stuttgart, Germany	
**FUNDER(S)**	Roche Pharma AG and AbbVie	

TABLE 1 Visit schedule and assessments – Flowchart

	**4 weeks**	**Induction treatment (18 weeks)**											**12 months**	**3 months**	
		**C1D1**	**C1D3**	**C1D8**	**C1D15**	**C2D1**	**C2D7**	**C3D1**	**C4D1**	**C5D1**	**C6D1**	**EOITA**	**MAINT**	**Follow-up visit**	**End of study visit (EOSV)**
**INVESTIGATIONS**	**Screening**	D1	D3	D8	D15	D22 +/-3d	D28	D43[£] +/-3d	D64[£] +/-3d	D85[£] +/-3d	D106[£] +/-3d	D127[£] +/-3d	First visit[§§, §§§] after **4** weeks thereafter Visits every **8** weeks (+/- 5 d)	One visit 6 weeks after last MAINT visit	6 weeks after follow-up visit
**Baseline evaluation, staging and response assessment**															
Informed consent from patient/legal representative	1														
Medical history and demographic data	1														
Bone marrow biopsy	1**														
PET-CT whole-body or CT neck till pelvis (according to local policy)	1														
Ophthalmological examination (Slit lamp)	1											1[££]	1[££]		
Lumbar puncture for flow cytometry	1[§]											1[§]			
Gadolinium-Enhanced MRI Brain (Spine)	1***					1		**1**		1		1	1[§§]		1
**PK Analyses for obinutuzumab and venetoclax**															
Lumbar puncture for PK analyses			1		1		1								
Peripheral blood collection for PK analyses		1*[#]	1[#]		1[#]		1[#]						1[#, §§§]		
**Samples translational studies**															
Paraffin embedded tissue collection for FoundationHemeOne Analyses (optional)[x]	1														
**Clinical and lab investigations**															
Height/Weight	1														
Pregnancy test for women of childbearing potential (WCBP)	1	Monthly (+/- 5 days)												1	
Electrocardiography	1														
Virology for HIV, hepatitis B and C[x]	1														
ECOG performance status	1	1				1		**1**	1	1	1	1	1	1	1

	4 weeks	Induction treatment (18 weeks)											12 months	3 months	
		C1D1	C1D3	C1D8	C1D15	C2D1	C2D7	C3D1	C4D1	C5D1	C6D1	EOITA	MAINT	Follow-up visit	End of study visit (EOSV)
**INVESTIGATIONS**	**Screening**	D1	D3	D8	D15	D22 +/-3d	D28	D43[£] +/-3d	D64[£] +/-3d	D85[£] +/-3d	D106[£] +/-3d	D127[£] +/-3d	First visit[§§, §§§] after **4** weeks thereafter Visits every **8** weeks (+/- 5 d)	One visit 6 weeks after last MAINT visit	6 weeks after follow-up visit
Haematology/biochemistry (see legend)	1	1[£££]			1[£££]	1[£££]		1[£££]	1[£££]	1[£££]	1[£££]	1	1	1	1
Clotting tests	1	1			1	1									
Physical examination[xx]	1	1				1		1	1	1	1	1	1	1	1
Vital signs[x]	1	1				1		1	1	1	1	1	1		
Registration after verification of eligibility criteria	1[##]														
**Treatment**															
Assessment for dose limiting toxicities (DLT)						1		1****							
Obinutuzumab 1000mg IV		1		1	1	1		1	1	1	1				
Venetoclax (600 / 800 / 1000mg) daily (oral)		1	1	1	1	1	1	1	1	1	1	1	1		
Adverse events (AEs)		1	1	1	1	1	1	1	1	1	1	1	1	1*****	
Concomitant medications	1	1	1	1	1	1	1	1	1	1	1	1	1		

C = Cycle; D = Day; EOITA = End of induction treatment assessment; MAINT = Maintenance treatment for a maximum of 12 months (first visit 4 weeks after EOITA [+/- 1 week]); EOSV = End of study visit;
Haematology includes: Absolute leucocyte count, absolute neutrophil count, absolute lymphocyte count, haemoglobin, absolute platelet count; Biochemistry includes: ASAT, ALAT, gamma-GT, bilirubin, creatinine, LDH; Clotting tests: PTT/INR
Screening period can take up to 28 days from registration

* 1 pre-infusion, only on C1D1 (C1D2 if applicable- for further Information see 2.1) also post-infusion PK sample
** If PET/CT shows no BM infiltration, BM biopsy can be omitted
*** MRI spine only if suspicion for meningeal involvement
**** **final DLT assessment**
***** AEs only to be assessed and reported up to 42 days after last intake of IMP (follow-up visit)
# Remaining volume will not be stored at the site. It must be discarded.
§ Only in case of clinical suspicion for meningeal involvement
§§ During maintenance treatment, MRI brain is conducted once 4 weeks after EOITA and thereafter every 8 weeks for the first 6 months and every 12 weeks thereafter
§§§ PK sampling only once 4 weeks after EOITA
£ plus/minus 3 days
££ only in case of initial involvement

£££ Blood samples are allowed to be drawn within 24 hours before obinutuzumab infusion, if not together with PK sampling

x not to be documented in CRF

xx Only be documented in the CRF at screening, at other visits in case of clinically relevant abnormal findings, the investigator has to document an AE on the corresponding CRF-page.

Registration has to be performed after completion of all screening investigations and as close as possible to the start of the study medication

Responsibilities

**Sponsor**	Institution:	Klinikum der Landeshauptstadt Stuttgart gKAöR represented by the management board
	Address:	Kriegsbergstraße 60, 70174 Stuttgart, Germany
**Coordinating Investigator** "Leiter der Klinischen Prüfung/LKP" (in accordance with German Drug Law)	Name:	Prof. Dr. G. Illerhaus
	Institution:	Klinikum der Landeshauptstadt Stuttgart gKAöR
	Address:	Kriegsbergstrasse 60, 70174 Stuttgart, GERMANY
	Telephone:	+49 - 711 - 278 - 30400
	Fax:	+49 - 711 - 278 - 30409
	E-mail:	g.illerhaus@klinikum-stuttgart.de
**Medical trial coordinator**	Name:	Dr. med. Julia Wendler
	Institution:	Klinikum der Landeshauptstadt Stuttgart gKAöR
	Address:	Kriegsbergstrasse 60, 70174 Stuttgart, Germany
	Telephone:	+49 - 711 - 278 - 42515
	Fax:	+49 - 711 - 278 - 30409
	E-mail:	j.wendler@klinikum-stuttgart.de
**Clinical methodologist**	Name:	PD Dr. Dr. Benjamin Kasenda
	Institution:	Universitätsspital Basel Medizinische Onkologie
	Address:	Petersgraben 4, Basel, Schweiz
	Telephone:	+49 - 171 - 3017039
	E-mail:	Benjamin.kasenda@usb.ch
**Registration**	Institution:	**Clinical Trials Unit** Medical Center – University of Freiburg,
	Address:	Elsaesser Str. 2, 79110 Freiburg, Germany
	Fax:	+49 - 761 - 270 - 74390
	Telephone:	+49 - 761 - 270 - 77810
**Data Management**	Name	Barbara Schilling
	Institution:	**Clinical Trials Unit** Medical Center - University of Freiburg
	Address:	Elsaesser Str. 2, 79110 Freiburg, Germany
	Telephone:	+49 - 761 - 270 - 77810
	Fax:	+49 - 761 - 270 - 73730
	E-Mail:	barbara.schilling@uniklink-freiburg.de
**Pharmacovigilance (PV)**	Institution:	**Clinical Trials Unit** Medical Center - University of Freiburg
	Address:	Elsaesser Str. 2, 79110 Freiburg, Germany
	Fax:	+49 - 761 - 270 - 74390
	E-mail:	stuz-pv@uniklinik-freiburg.de
**Project management**	Name:	Elvira Burger
	Institution:	**Clinical Trials Unit** Medical Center - University of Freiburg
	Address:	Berliner Allee 6, 79110 Freiburg, Germany
	Telephone:	+49 - 761 - 270 - 73780
	Fax:	+49 - 761 - 270 - 74250
	E-mail:	elvira.burger@uniklinik-freiburg.de

**Monitoring (CRA(s))**	Name:	Dr. Simone Weber
	Institution:	Medical Center – University of Freiburg<br>Division of Hematology/ Oncology (Med I)
	Address:	Hugstetter Strasse 55, 79106 Freiburg, Germany
	Telephone:	+49 - 761 - 270 - 72871
	Fax:	+49 - 761 – 270 - 33180
	E-mail	Simone.weber@uniklinik-freiburg.de
**Monitoring (CRA(s))**	Name:	Cora Steinheber
	Institution:	Stuttgart Cancer Center Klinikum der Landeshauptstadt Stuttgart gKAöR
	Address:	Kriegsbergstrasse 60, 70174 Stuttgart, Germany
	Telephone:	+49 - 711 - 278 - 57420
	Fax:	+49 - 711 - 278 - 35649
	E-mail:	c.steinheber@klinikum-stuttgart.de
**Other medical/ technical departments/ Institutes**	Institution:	AbbVie
	Name:	Richard F. Arrendale, Ph.D.
	Address:	Dept. R46W, Bldg. AP13A-2,1 North Waukegan Road, North Chicago, IL 60064, USA
	Telephone:	+1 - 847 - 937 - 0889
	Fax:	+1 - 847 - 938 - 9898
	E-mail:	richard.arrendale@abbvie.com
	Institution:	PPD
	Name:	Megan Wiberg
	Address:	2246 Dabney Road, Richmond, VA 23230, USA
	Telephone:	+1 - 804 - 977 - 8230
	Fax:	+1 - 804 - 977 - 8112
	E-mail:	Megan.Wiberg@ppdi.com, richmond_data@ppdi.com
**Scientific Steering Committee**	Name:	PD Dr. Dr. Benjamin Kasenda
	Institution:	Universitätsspital Basel, Medizinische Onkologie
	Profession:	Medical Oncologist/ Methodologist
	Address:	Petersgraben 4, Basel Schweiz
	Telephone:	+49 - 171 - 3017039
	E-mail:	benjamin.kasenda@USB.ch
	Name:	Prof. Dr. G. Illerhaus
	Institution:	Klinikum Stuttgart
	Profession:	Haematologist/Oncologist
	Address:	Kriegsbergstrasse 60, 70174 Stuttgart, Germany
	Telephone:	+49 - 711 - 278 - 30400
	Fax:	+49 - 711 - 278 - 30409
	E-mail:	g.illerhaus@klinikum-stuttgart.de
	Name:	Dr. Elisabeth Schorb
	Institution:	Medical Center – University of Freiburg<br>Division of Hematology/ Oncology
	Profession:	Haematologist/Oncologist
	Address:	Hugstetter Strasse 55, 79106 Freiburg, Germany
	Telephone:	+49 - 761 - 270-35360
	Fax:	+49 - 761 - 270-73570
	E-mail:	elisabeth.schorb@uniklinik-freiburg.de

1 Background and rationale

1.1 Scientific background

Primary central nervous system lymphoma (PCNSL) is a diffuse large B-cell lymphoma (DLBCL), which exclusively invades the CNS compartment. It accounts for 3-4% of all primary brain tumours and 4-6% of extra-nodal lymphomas (3). The incidence of PCNSL in immunocompetent patients has been steadily increasing over the last years now reaching about 0.48 / 100.000 per year (4,5).

HD-MTX combined with HD-cytarabine (HD-AraC) followed by whole brain radiotherapy is currently regarded standard treatment for newly diagnosed PCNSL (6). High-dose chemotherapy with autologous stem cell support (HCT-ASCT) also leads to very high remission rates and long-term survival up to 10 years in eligible patients (7–10). However, about 20% patients treated with the combination HD-MTX plus HD-AraC do not respond to treatment and a third of responding patients suffer relapse (6). Moreover, even after completion of HCT-ASCT and achieving a complete remission, still 35% of patients eventually relapse (11). Only few single agents have been prospectively investigated in the relapse or refractory situation, namely temozolomide (N=23, median age 60 years, overall response rate [ORR] 26%, median progression free survival [PFS] 2.5 months) (12), pemetrexed (N=11, median age 70 years, ORR 55%, median PFS 5.7 months) (13), and topotecan (N=15, median age 56 years, ORR 40%, median PFS 2 months) (14). A recent trial investigating the combination of rituximab and temozolomide was stopped early due to slow accrual and because the activity of this combination was not convincing (N=16, median age 63 years, ORR 36%, median PFS 1.6 months) (15). In summary, there is no standard treatment for relapsed or refractory PCNSL and its choice highly depends on previous treatments and clinical performance status (16).

1.2 Overview of investigational medicinal products (IMPs)

Venetoclax is a selective, orally bioavailable small molecule inhibitor of BCL-2 (B-Cell Lymphoma 2), an anti-apoptotic protein in the BCL-2 family (17). The BCL-2 family of proteins includes pro-apoptotic and anti-apoptotic proteins. Anti-apoptotic proteins, such as BCL-2, block cell death by sequestering and preventing the activation of pro-apoptotic proteins (18,19). Overexpression of anti-apoptotic proteins of the BCL-2 family is implicated in tumour development (20–22).

In systemic DLBCL outside the CNS, two subtypes have been identified based on gene expression profiling, the germinal centre B-cell (GCB)-like and activated B cell (ABC)- like type (23). PCNSL cannot clearly be categorized into GCB or ABC, but it shares expressions of the BCL6 protein and ongoing mutation of the IGH locus with the GCB-subset of DLBCL (24,25). In contrast, the most frequent cytogenetic abnormality in PCNSL (25) is gain of chromosome 18q21, which is associated with the ABC subtype (23). The ABC subtype is also characterized by constitutive activation of the nuclear factor (NF)-JB pathway (26), which is also activated in PCNSL and could explain the high proliferation activity and low level of apoptosis observed in PCNSL (27). Given that the most common genetic imbalance in PCNSL are gains of 18q21, which includes the BCL2 locus (28,29), provides a biological rationale for targeting BCL2 in PCNSL treatment.

Venetoclax, as single agent, has been investigated previously in two large international single-arm trials recruiting patients with relapsed or refractory chronic lymphatic leukaemia (30,31). The most important safety issue was tumour lysis syndrome due to the high efficacy of venetoclax in patients with high lymphoma load. Apart from this, venetoclax was well tolerable in the respective patient populations of which up to a third were 70 years and older. The most common grade 3 or higher toxicities were neutropenia (41% in (31) and 40% in (30)), anaemia (12% in (31) and 18% in (30)) and thrombocytopenia (12% in (31) and 16% in (30)).

Monoclonal antibodies only show minimal transport across the blood brain barrier; however, clinical responses to rituximab monotherapy in PCNSL have been reported (32). Most recently, the IELSG 32 randomized trial has proven that addition of rituximab to standard HD-MTX/HD-AraC chemotherapy in newly diagnosed PCNSL leads to significantly improved response, progression free and overall survival (33). Based on this, CD-20 directed monoclonal antibodies can definitely be considered active for PCNSL and should be considered in any line of treatment.

The type II CD-20 antibody obinutuzumab was specifically developed and glycoengineered to have increased direct cell death activity through increased affinity for Fc-delta RIIIa. Preclinical evidence suggests that obinutuzumab (GA101) is superior to rituximab in killing lymphoma cells in immune cell effector and whole-blood depletion assays and to have increased antitumor activity in human xenograft models (34). A recent randomized phase II trial compared rituximab with obinutuzumab in relapse FL and showed that obinutuzumab leads to higher response rates (45% versus 27%) (35). In 25 patients with heavily pre-treated DLBCL, single agent obinutuzumab also showed a response rate of 32% (including patients not responding to rituximab previously) (36). The combination of venetoclax and obinutuzumab has been investigated in 32 patients with relapsed or newly diagnosed CLL (37). The response rates were up to 100% (by investigator assessment) in those patients with relapsed disease. Major adverse events included neutropenia (34%) and infections (19%). In a recent phase 1B study, venetoclax was combined with obinutuzumab in 16 patients with previously treated or untreated CLL (38). The combination did not show any unexpected safety signals with venetoclax dosing up to 600mg

1.3 Trial purpose and rationale

Based on our own systematic MedLine review using PubMed (keywords: pcnsl, venetoclax, obinutuzumab; filter: humans) and information from the respective companies (Roche Pharma AG and AbbVie), neither venetoclax nor obinutuzumab have been investigated as single agent or in combination in patients with PCNSL.

Given that the most common genetic imbalance in PCNSL are gains of 18q21, which includes the BCL2 locus (28,29), provides a biological rationale for targeting BCL2 with venetoclax in PCNSL treatment. Furthermore, the IELSG32 trial has established anti-CD20 directed therapy with rituximab in PCNSL. Given that obinutuzumab has additional pharmacodynamics features compared to rituximab, it is consequent to test it in relapsed or refractory PCNSL as well. Based on data available, there are no additional concerning safety issues when combining venetoclax with obinutuzumab, of note, there is no evidence for higher risk of side effects with increased venetoclax doses. Therefore, we propose a single arm multicentre phase IB dose-escalation trial to investigate a chemotherapy free treatment of venetoclax in combination with obinutuzumab in relapsed or refractory immunocompetent PCNSL patients.

1.4 Rational for choice of control interventions/comparators

This is a non-comparative trial; all patients will receive venetoclax and obinutuzumab at different dose levels of venetoclax to investigate its respective pharmacokinetics.

1.5 Rational for dose selection

The FDA approved dosage for venetoclax single agent in CLL is 400mg daily (39). Because of observed tumour lysis syndromes, a ramp up dosing scheme has been implemented in recent studies for systemic lymphoma (31). The particular issue of tumour lysis syndrome is not expected in PCNSL because of the substantially lower lymphoma load as compared to CLL or other systemic lymphoma outside the CNS. In a recent phase 1B study, venetoclax was combined with obinutuzumab in 16 patients with previously treated or untreated CLL (38). The combination did not show any unexpected safety signals with venetoclax dosing up to 600mg. Other studies have tested venetoclax with doses up to 1200mg in non-Hodgkin lymphoma, of note, without signals of an association between incremental dosing and toxicity, no maximal tolerated dose could be defined (31,40). In PCNSL, there is always the issue of drug delivery to the CNS and there are no data on the availability of venetoclax in the CNS. Furthermore, patients with PCNSL require an early response to improve the clinical status. Therefore, it should be aimed to achieve the maximal possible drug concentration right from start of therapy.

These arguments have led to the design of the study described herein where we have three venetoclax dosing groups (600mg, 800mg, and 1000mg). We have chosen not to start with the standard dose (400mg), because we assume higher CSF levels and therefore a higher chance for lymphoma response. Patients with PCNSL, particularly in the relapse/refractory situation, are at high risk for another relapse or progression, even if a remission has been achieved (41,42). Therefore, effective maintenance strategies in patients who have achieved a response are urgently needed. The well tolerable oral BCL-2 inhibitor venetoclax is a promising candidate to be investigated for maintenance therapy which we plan in this study.

Obinutuzumab will be given at the approved dosage of 1000mg and schedule (day 1 (and 2 if applicable), 8, 15 during the first cycle and on day one of each following three-week cycle). In contrast to the first CD20 antibody rituximab, obinutuzumab has been developed including a dose finding phase. The fact that the blood brain barrier is likely disrupted with active lymphoma disease in the CNS has established a dose dense application of rituximab in the IELSG32 trial (33). We will follow the same principle in the study described herein.

1.6 Risk-benefit assessment

Patients with relapsed or refractory PCNSL have a very poor prognosis; 50% die within the first 12 months of relapse (43). A significant proportion of relapsed/refractory PCNSL patients is not fit enough for conventional poly agent chemotherapy; this particularly accounts for the elderly population (≥ 60 years of age) who comprise about 50% of all PCNSL patients (44). Therefore, there is a great unmet need for these patients and new treatment approaches with novel agents are urgently needed.

Based on previous studies in a comparable and in part even frailer patient population with CLL, the combination of venetoclax and obinutuzumab is feasible and safe (45). Main side effects include haematological toxicities, which are usually manageable with appropriate growth factor use and antibiotics if indicated. The risk for tumour lysis syndrome in CNS lymphoma is

neglectable, because the overall lymphoma mass (burden) is much smaller compared to e.g. CLL. Given the possible benefit of this drug combination, the investigators believe that the amount of benefit clearly outweighs the risk for the individual patient.

With respect to non-substance-specific risk-benefit assessment, the investigations and assessments that are performed throughout the study, mainly include procedures that are performed in patients with this indication on a routine basis in outpatient clinical patient care; of note, one lumbar puncture is considered standard of care at diagnosis or relapse/progression of PCNSL. The only exceptions are the two additional lumbar punctures to obtain samples for pharmacokinetic (PK) analyses. However, such punctures are usually associated with a relatively low risk for bleeding, pain or infections. These punctures are done in the outpatient setting and are frequently conducted in centres treating patients with haematological malignancies. Therefore, the additional risks of these additional punctures beyond routine patient care are considered well balanced and of minor concern.

Moreover, the fact that patients included in this trial are closely monitored by an experienced team in shorter outpatient intervals as in routine care, provides a substantial medical benefit by itself.

In summary, there is substantial evidence for a positive benefit-to-risk-ratio for the patients included in this trial for both substance-specific and non-substance-specific assessments.

2 Objectives and endpoints

2.1 Primary objective

- To investigate the PK of venetoclax and obinutuzumab in the CSF in patients with relapsed or refractory PCNSL.

The combination of venetoclax and obinutuzumab has never been investigated in PCNSL. Apart from this, there is no evidence that each of the components penetrates into the CNS compartment. We therefore consider the PK of each agent as the main interest of our study followed by preliminary efficacy. Assuming a dose response relationship, the highest venetoclax and obinutuzumab concentrations should be reached in the CNS compartment to unfold maximal anti-lymphoma activity. The concentration measured in the CSF is the most pragmatic approach to proxy the effective anti-lymphoma concentration of both drugs in the lymphoma and the brain parenchyma. We will take one pre-dose PK sample on day 1 (before first obinutuzumab infusion and before first venetoclax intake); given that this will be negative before any drug exposure, we can conclude that there is also no measurable drug concentration in the CSF. By this, we can spare a pre-dose CSF sample. Therefore, in total there will be six PK samples from PB (pre-dose and post-dose day 1 (day 2 if applicable), pre-dose day 3, pre-dose day 15, pre-dose day 28, and at the first maintenance visit 4 weeks after end of induction treatment assessment) and three PK CSF samples (day 3, day 15, and day 28 [all pre-dose]). In the absence of infusion-related reaction or hypersensitivity to the 100 mg test dose of obinutuzumab and given an adequate medical supervision during infusion period, the remaining 900 mg can be applied directly after the test dose on day 1 (see section 6.1.2). Otherwise the remaining 900 mg obinutuzumab can be applied on day 2 and subsequently post dose PK sample will be taken 30 minutes after termination of infusion. The main measure to investigate the CNS penetration of the respective drugs will be the ratio of the CSF concentration over the concentration in PB. All samples for PK analyses on the respective days will be documented by date and time point (by minute). Drug concentrations in the PB and CSF will be measured by

dedicated laboratories in the US (assigned by Roche and AbbVie) using established assays by Roche Pharma AG and AbbVie.

2.2 Secondary objectives

- To investigate preliminary clinical efficacy such as lymphoma response and event-free survival (We will use the standardized IPCG response criteria (1) to categorize the lymphoma response.)
- Dose limiting toxicities (DLTs) during escalation of dosing groups as defined by CTCAE (version 5.0) and recommended phase II dose

2.3 Tertiary objectives

- To investigate frequency of gene alterations in DNA extracted from lymphoma tissue using the FoundationOneHeme platform.

Recent deeper insights into the mutational landscape of PCNSL have provided a stronger rational to use targeted agents in PCNSL. Especially PCNSL dependency on chronic B-cell receptor pathway activation seems to be a promising mechanism to target, e.g. with ibrutinib (46,47). As outlined above, the most frequent cytogenetic abnormality in PCNSL (25) is gain of chromosome 18q21, which also encodes the BCL2 gene. In the study described herein, we also aim to provide more insights into the mutational landscape of PCNSL and to explore possible associations between certain genetic alterations and response to treatment. We aim to use the established FoundationOneHeme platform to conduct these analyses based on formalin fixed tissue (48). No re-biopsy is mandatory at study inclusion.

2.4 Safety objectives

- To investigate safety and tolerability (dose limiting toxicity [DLT]) of venetoclax and obinutuzumab at different dosing levels

We will assess the DLT of the combination. Laboratory assessments will be analysed graded according to the CTCAE (version 5.0). AEs will be summarized by CTCAE term and grade, and presented in total and per dose level. In addition, grade ≥ 3 AEs and AEs related to trial treatment (relation to trial treatment is either “related” or “un-related”) will be summarized separately. All SAEs, including SARs and SUSARs will be fully listed. Please see section 10 for further details.

2.5 Primary objective and endpoint

PK of venetoclax and obinutuzumab expressed by the respective PB concentration and CSF concentration (measured in µg/ml) measured within the *first month since start of treatment on day 3, 15 and 28.* In total three CSF and serum samples for PK analyses will be taken and concentration of the respective drug will be measured by validated assays from Roche Pharma AG and AbbVie.

2.6 Secondary objectives and endpoints

- Best lymphoma response achieved during induction, it is defined as the best documented response (CR, PR, SD, or PD) according to the IPCG response criteria (1) as evaluated by the local neuro-radiologist during induction treatment.

- Dose limiting toxicities (DLTs) during escalation of dosing groups as defined by CTCAE (version 5.0) (for details see 2.8.1) and recommended phase II dose.
- Progression free survival 1 (PFS1), which is defined as the time from the date of first dose until date of progression, relapse or death, whichever occurs first. Progression or relapse of the lymphoma will be evaluated by brain MRI as per schedule of assessment. In case of clinical suspicion of progression or relapse, brain MRI can be done as clinically indicated.
- Failure free survival (FFS), which is defined as the time from the date of first dose until date of progression, death, or study termination due to toxicity.
- Time to initiation of subsequent treatment, which is the time from first dose until initiation of subsequent treatment off trial for whatever reason. Patients who progress and do not receive any other treatment or die before initiation of another treatment off trial will be censored at the date of the respective event. Patients on study without progression and who continue study treatment will be censored at last date of follow-up.
- Sustained response time, this will be calculated for all patients achieving CR or PR, it is the time from the date of first CR or PR until progression or death. Patients never achieving at least PR will be excluded from this analysis.
- Overall survival (OS); this will be calculated from the date of first dose until death due to any cause. Patients alive will be censored at the last date of follow-up.
- Progression-free survival 2 (PFS2), which is defined as the time from the start of maintenance (eligibility for maintenance, please see 3.1) venetoclax treatment at week 12 until date of progression, relapse or death, whichever occurs first. Patients not reaching week 12 will be excluded from analysis of this endpoint (landmark approach).
- Toxicity as evaluated by CTCAE (version 5.0).

2.7 Tertiary objectives and endpoints

- To investigate distribution and frequency (proportion) in which FoundationOne Heme® One analyses based on formalin fixed tissue were technically successful (irrespective of alterations identified).
- To investigate distribution and frequency (proportion) in which FoundationOne Heme® One analyses based on formalin fixed tissue identified genetic alterations in the lymphoma cells expressed as allelic frequency in total and stratified by responding and non-responding patients.

2.8 Definitions

2.8.1 Definition of dose limiting toxicity (DLT)

We will only consider a DLT event as effective in altering the planned escalation (e.g. from cohort 1 to cohort 2) <u>if the DLT defining event occurs within the first 6 weeks (including day 43) of induction treatment of the current cohort.</u> We have chosen the time period of 6 weeks, because if patients experience toxicities, these are expected to occur within the first 4 to 6 weeks of treatment. The following events as per CTCAE classification (version 5.0) will be considered as DLT:

- Trial therapy related death

- Grade 4 neutropenia not resolved after 14 days despite growth factor support
- Grade 3 to 4 febrile neutropenia (according to CTCAE: ANC <1000/mm3 and a single temperature of >38.3°C or a sustained temperature of ≥38°C (100.4 degrees F) for more than one hour.)
- Grade 4 thrombocytopenia not resolved after 14 days
- Grade 2 or higher bleeding associated with thrombocytopenia
- Any other grade 3 or higher haematological or non-haematological adverse event related to one or both IMPs that does not resolve to at least grade 2 or to baseline value within 3 weeks since onset by complete drug discontinuation and supportive care if applicable *except*:
    - Alopecia
    - Nausea and diarrhoea adequately treated

2.8.2 Definition of minimum safety evaluation requirements

The following minimum safety evaluation requirements need to be fulfilled to include a patient into the DLT appraisal: The patient has received ≥ 80% of venetoclax doses (according to the patient diary) and ≥ 80% doses of obinutuzumab in the first 6 weeks. A further requirement is that the patient is observed for 6 weeks following the first dose of the IMPs and has completed all relevant safety evaluations, or the patient experiences DLT during the first 6 weeks.

3 Clinical trial plan

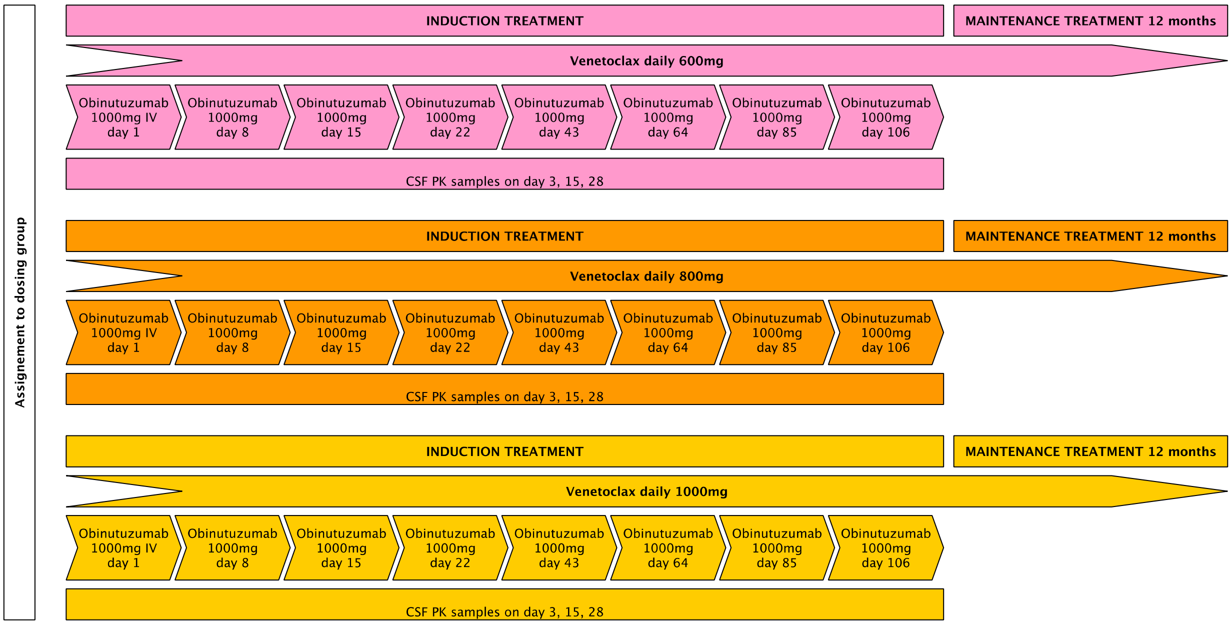

FIGURE 1: Trial design. The primary aim of the study is to investigate the PK in the CSF. It is planned to treat 5 patients in each dosing group starting at 600mg venetoclax. Obinutuzumab dose will be flat at 1000mg in all dosing groups. Please see 3.1.1 for prespecified rules for dose escalation and de-escalation.

3.1 Trial design

This is a single arm study dose escalation phase IB study (three venetoclax dosing groups: 600mg [N=5], 800mg [N=5], 1000mg [N=5]) conducted at two German sites with the aim to define the most promising dosing combination of venetoclax and obinutuzumab regarding CSF penetration in relapsed or refractory PCNSL.

During the combination induction phase, obinutuzumab at 1000mg will be given for 6 cycles (day 1 (and 2 if applicable), 8 & 15 [first cycle] and on day 1 in cycles 2 till 6; cycles repeated every 21 days) together with daily venetoclax (600mg [N=5], 800mg [N=5] or 1000mg [N=5]) (**Figure 1**). Assignment to the respective dosing groups will be consecutively, starting at 600mg.

To assure patient safety, we use the BOIN (Bayesian Optimal Interval) design (2) to appraise feasibility of dose escalation or dose de-escalation of venetoclax. To pre-specify decision boundaries, we have set the target DLT at 30%; for 3 cohorts with 5 patients each, this translates into the following boundaries: Threshold for escalation <= 0.2364907, threshold for de-escalation >= 0.3585195. Translating these boundaries into decision boundaries, the following rules apply: If <=1 DLT occurs at a certain dose level, the dose can be escalated to the next higher dose level; if >=2 DLT occur, the dose will be de-escalated to the next lower dose level. In example, if <=1 DLT occurs at 600mg, the dose will be escalated to 800mg for the next 5 patients. If the >=2 DLTs occur at 600mg, the dose will be reduced to 400mg (also see **Table 2, next page**).

The total time of this induction phase will be 18 weeks (six 3 weekly cycles). During induction treatment, there will be 4 MRI scans: cycle 2 (day 1), cycle 3 (day 1), and cycle 5 (day 1); the final MRI scan to complete the induction phase will be conducted 21 days after the last obinutuzumab infusion (end of induction phase). Afterwards, during maintenance treatment MRI scans are planned 4 weeks after end of induction treatment assessment (EOITA), thereafter every 8 weeks for the first 6 months and every 12 weeks thereafter. Last MRI will be conducted at end of study visit (EOSV) 6 weeks after termination of maintenance treatment.

To go on to venetoclax maintenance phase, patients need to fulfil both following criteria after induction treatment:

- At least stable disease as per IPCG response criteria at EOITA.
- No decline in clinical performance status compared to baseline at EOITA.

3.1.1 Dose limiting toxicities (DLTs) and venetoclax dose escalation

Importantly, patients will not be treated in parallel in the different dosing groups – the first 5 patients will be treated in cohort 1 (600mg), the next five patients in cohort 2 (800mg), and the last 5 patients in cohort three (1000mg). Only if zero or only 1 DLT (see definition in section 2.8.1) occurs in 5 patients and all patients fulfilled minimum safety evaluation requirements, the next cohort will be opened as planned. *Patient recruitment will be stopped and not resumed until the safety assessment for the DLTs at the given dose level has been completed (see section 10.2.8).* Specifically, based on the above mentioned BOIN design (2), we define the following rules for escalating or de-escalation:

**Rule**	**Target number of patients treated at each dose level (dose level of venetoclax)**

	N=5 (600mg)	N=5 (800mg)	N=5 (1000mg)
Escalate if number of DLT is smaller or equal to:	1	1	1
De-escalate if number of DLT is larger or equal to:	2	2	2
Terminate study if number of DLT is larger or equal to:	4	4	4

TABLE 2: Rules for venetoclax dose escalation and de-escalation following the BOIN design.

In case a patient is not assessable during DLT time, i.e. until day 43 inclusively after the first dose of IMP for whatever reason (e.g. death definitely unrelated to study drug, withdrawal of consent, lost to follow-up) and therefore does not fulfil minimum safety evaluation requirements (see section 2.8.2), we will replace the patient to guarantee that there are always five patients assessable for DLT in each of the intended dosing groups.

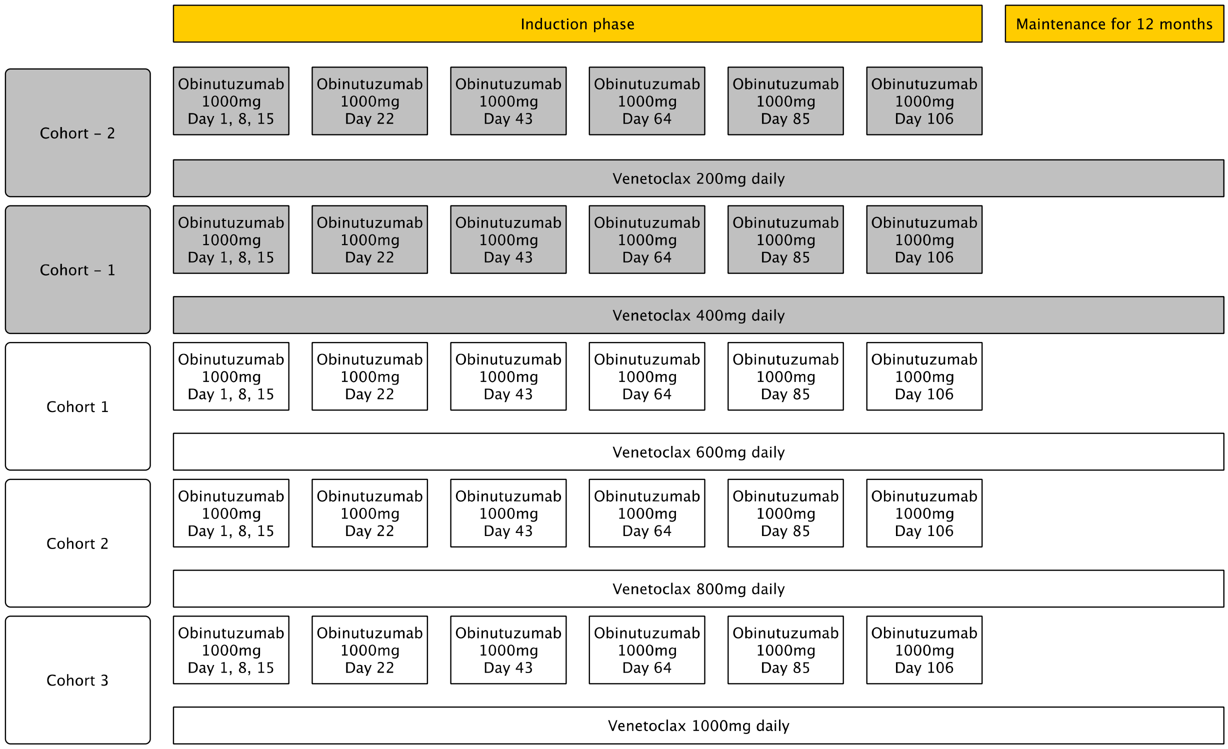

FIGURE 2: Dosing groups

3.2 Treatment arms

This is a single arm, non-comparative study, therefore all patients will receive both IMPs (venetoclax and obinutuzumab). For further details please see section 6.

3.3 Treatment duration

All patients are planned to receive six cycles of induction treatment (combination of obinutuzumab and venetoclax, 18 weeks [six 3 weekly cycles]) and if at least stable disease without clinical deterioration has been achieved, patients will go on to maintenance treatment for 12 months (52 weeks) with venetoclax. Therefore, the total planned treatment duration per patient will be 70 weeks. In case of disease progression or unacceptable toxicities, treatment will be stopped.

3.4 Number of patients

We will include 15 patients (5 in each dosing group). In case patient is not assessable for toxicity during DLT time for whatever reason, the patient will be replaced.

3.5 Participating sites

This study will be conducted at two German sites (Stuttgart and Freiburg).

3.6 Recruitment rate

We expect to recruit 1 patient per month (in total from both sites) based on previous similar studies at the respective centres.

3.7 Translational studies

The objective of FMI testing (FoundationOne Heme® platform) is to gain more insights into the mutational landscape of PCNSL. There will be no repeat brain biopsy unless clinically indicated. All samples will be pseudonymized. Results of analyses will be disclosed to the treating physician in a standardized report (BioPharma CLIA) as soon as each report from each individual sample has been issued. The treating physician will disclose results from FMI testing to the patient at latest at the time of lymphoma progression. At that time point, results from FMI testing may positively support further treatment decision making. However, if the patient asks for the results before lymphoma progression, the treating physician will have to disclose the results. In that case, it should be emphasized that the results should not alter the treatment plan if the patient tolerates current treatment and shows lymphoma response. This is because it is yet unclear to what extend longitudinal FMI testing really helps to positively alter patient management.

3.8 Trial timetable

Enrolment of first patient, first patient in (FPI)	2nd quarter 2020
Enrolment of last patient, last patient in (LPI)	2nd quarter 2022
End of trial defined as last patient last visit (LPLV)	4th quarter 2023

Final analysis and study report	3rd quarter 2024

4 Trial population and selection criteria

4.1 Target population

Patients will only be allowed to enter the trial if they provide written informed consent to their participation (following full explanation of the trial) (see section 5.1).

4.1.1 Health condition studied

The study population includes immunocompetent patients with relapsed or refractory PCNSL.

- *Relapsed PCNSL* is defined as growth or re-occurrence of the lymphoma after achieving at least PR or CR after or during latest therapy. To assign a complete remission (CR), it should have been confirmed on at least two contrast-enhanced MRI scans of the brain with at least 4-week interim period based on radiology reports from routine medical care.
- *Refractory PCNSL* is defined as no radiological response (SD or PD) as per the IPCG criteria to prior chemotherapy regimen(s), at least one of them containing high-dose methotrexate

All evaluations of response will follow the IPCG criteria (49). All patients require histological or cytological proven PCNSL at initial diagnosis and need to have received at least one prior HD-MTX containing chemotherapy (≥ 1 g/m^2 MTX). Biopsy at relapse or progression before entering the trial is not mandatory, but recommended if previous relapse free time is longer than 24 months. Lymphoma manifestation outside the CNS at progression/relapse needs to be ruled out by body CT scans or PET-CT scans. A bone marrow biopsy/aspiration is also required if PET-CT scan cannot rule out bone marrow infiltration.

4.1.2 Gender distribution

No gender ratio has been stipulated in this trial as the results of the preclinical and clinical studies did not indicate any gender effect of the trial treatment in terms of efficacy and safety.

4.2 Inclusion criteria

Patients eligible for inclusion in this trial must meet all of the following criteria:

1. Age at inclusion ≥ 18 to 80 years, in case of ECOG 0 to 1 age up to 85 years
2. Eastern Cooperative Group performance status (ECOG) ≤ 3
3. Evaluable lymphoma manifestation in the CNS, either contrast-enhanced lesion in the brain parenchyma or measurable meningeal lesions.
4. Biopsy proven CD20 positive PCNSL at initial diagnosis or previous relapse/progression (re-biopsy at study inclusion is not mandatory for inclusion, but strongly recommended if time in remission is longer than 24 months).

5. At least one prior HD-MTX containing chemotherapy application (MTX dosed at ≥ 1 g/m^2 body surface area) before progression or relapse.
6. Confirmed relapsed or refractory PCNSL according to the IPCG response criteria with the following definition: Evidence of disease recurrence following PR/CR or uCR or no radiological response (SD or PD) as per the IPCG criteria to prior chemotherapy regimen(s), at least one of them containing high-dose
7. Absolute neutrophil count (ANC) of at least 1’500/µl
8. Platelet count of at least 50’000/µl
9. Adequate liver (alanine aminotransferase [ALAT] and AST ≤ 3.0 x upper limit of normal [ULN] and total bilirubin ≤ 1.5 x ULN) and kidney function (estimated ≥ 30ml/min creatinine clearance according to Cockgroft-Gault formula)
10. Written informed consent
11. Recovery from toxicity from previous anti-lymphoma treatment to ≤ grade 2

4.3 Exclusion criteria

Patients eligible for this trial must not meet any of the following criteria:

1. Known allergy to venetoclax or other components of the formulation
2. Known allergy to obinutuzumab or other components of the formulation
3. Primary ocular lymphomas *without* brain parenchymal involvement
4. Lymphoma relapse outside the CNS; extra CNS relapse needs to be ruled out by body CT scans (neck till pelvis) or PET-CT scans. *
5. Contraindications for lumbar puncture at the discretion of the clinical investigator
6. Prior exposure to obinutuzumab or venetoclax
7. Other additional anti-lymphoma treatment, e.g. chemotherapy or radiotherapy
8. Active hepatitis B or C
9. HIV seropositivity
10. Chronic use of immunosuppressive drugs, e.g. steroids for systemic autoimmune disease
11. Active infections requiring treatment
12. Other active malignancies (except non-melanoma skin cancer). Prior malignancies without evidence of disease for at least 5 years are allowed
13. Patient is pregnant or breastfeeding, or expecting to conceive or father children within one year of finishing venetoclax and 18 months for obinutuzumab.
14. Prior allogeneic haematopoietic stem cell or solid organ transplantation
15. Therapeutic intervention in setting of other former interventional clinical trial within 30 days before the first IMP administration in VENOBI study; simultaneous participation in registry and diagnostic studies or follow up of an interventional trial is allowed
16. Patient without legal capacity who is unable to understand the nature, significance and consequences of the trial
17. Known or persistent abuse of medication, drugs or alcohol
18. Person who is in a relationship of dependence/employment with the sponsor or the investigator
19. Administration of moderate or strong CYP3A inhibitors or inducers within 1 week of initiation of venetoclax dosing.

* In case CT is done, a bone marrow biopsy needs to be conducted. In case PET/CT shows no signs for bone marrow infiltration, a bone marrow biopsy/aspiration can be omitted. If the

relapse/progression occurs within 8 weeks of initial diagnosis and systemic lymphoma manifestation has been ruled out with one of the above-mentioned modalities, no repetitive staging investigations are necessary.

5 Enrolment and patient registration

5.1 Patient eligibility

If a patient appears to be eligible for the trial, the investigator will inform the patient about the trial and ask the patient for his/her written consent. It is imperative that written consent is obtained prior to any trial-specific procedures. The investigator will then record the details of these trial **patients on the following trial-specific lists:**

- **Subject Screening log:** for the documentation of trial patients who were checked for eligibility before and/or during the clinical trial. The following will be entered: consecutive screening number (e.g., 1, 2, 3 etc.), date of written consent (if obtained), as well as details on whether the patient was enrolled in the trial and, if not, the reason for not enrolling the patient. For all enrolled patients, the individual patient identification code[1] will be recorded on the subject screening log.
- **Subject identification log:** A confidential log of the names of all trial patients with the identification code[1] assigned to each patient at the time of enrolment in the clinical trial. With this list, the identity of each patient can be revealed. The list must be kept confidential and remain at the trial site. It must not be copied or otherwise be passed on! However, Sponsor representatives, clinical research associates (CRAs), auditors and representatives of competent authorities (CA) must be allowed to inspect the list on request.

5.2 Patient registration

The patient identification code assigned for the study (see section 5.1) will be entered on the registration form. The fully completed form will then be faxed to the central registration office (CTU) for registration:

Clinical Trials Unit
Medical Center - University of Freiburg
Fax: +49 761 270-74 390

Registration times:
Monday to Friday from 9:00 to 16:30

[1] Patient identification code: A unique trial-specific identification number which identifies the patient and consists of two parts: The first 2 digits correspond to the site number, the next 2 digits stand for a consecutive number of the patient enrolled at a particular site, for example: <01-01 (Site No. 1, Patient No. 1)>, 01-02 (Site No. 1, Patient No. 2) so that each patient is numbered uniquely across the entire database.

The central trial office will review all relevant pieces of information on the registration fax. It will then confirm the patient's enrolment in the trial by fax. Trial treatment can be initiated according to the protocol and assigned dosing group (dosing group cannot be chosen by the local investigator!).

5.3 Randomisation methodology

Does not apply to this study.

6 Treatment plan and procedure

The investigator or designee will instruct the patient to take venetoclax as per protocol; obinutuzumab will be administered at the study site under surveillance of study staff. All dosages prescribed and dispensed to the patient and any dose change or interruption must be recorded in the patient chart, CRFs and/or on drug accountability forms, as appropriate.

6.1 Dosing regimen and IMP administration

6.1.1 Venetoclax

Venetoclax is given daily as oral tablets (be taken within 30 minutes of completion a meal and with water) in three dosing groups: 600mg, 800mg, and 1000mg together with obinutuzumab. In case patients do not tolerate venetoclax despite dose reductions, patients will be taken off study treatments.

Patients are advised to take venetoclax always at 10:00 am daily (about 30 minutes after last breakfast). This is to assure relative consistency for PK analyses, especially during the first 4 weeks after starting treatment.

In previous studies with CLL patients, a ramp-up schedule to avoid fatal tumour lysis syndrome (TLS) was implemented. In PCNSL, lymphoma burden is much lower and not comparable to CLL, therefore the risk for TLS is not given. Therefore, we decided to skip ramp-up dosing and to directly start with the intended dose level (600mg, 800mg or 1000mg).

For details on improvement of treatment adherence please refer to section 9.11.

6.1.2 Obinutuzumab

Obinutuzumab will be given intravenously with 1000mg diluted to 10 mg/mL, administered at an initial rate of 25-50 mg/hour for a test dose of 100 mg, and increased by 50 mg/hour every 30 minutes in the absence of infusion-related reactions to a maximum of 400 mg/hour under adequate medical supervision. In the presence of infusion-related reaction or hypersensitivity during test dose or in the absence of adequate medical supervision, the remaining 900 mg obinutuzumab will be given on day 2 regarding cycle 1. Further, obinutuzumab will be administered on day 8 and 15 in the first cycle, afterwards always on day 1 every 3 weeks for a total of 6 cycles unless progression or unacceptable toxicity occurs in between. Patients with severe allergic reactions to obinutuzumab that do not allow continuing obinutuzumab are allowed to continue with venetoclax monotherapy.

Premedication and infusion schedule for obinutuzumab will be performed in accordance with local guidelines and the investigators' brochure.

After the infusion of obinutuzumab the patient will stay under observation for at least 1 hour at the study site. Study visits will be performed in line with administration schedule. Medical personnel and emergency equipment are available at all sites participating in this study.

We aim for obinutuzumab infusion time at 10:15 am during the first 4 weeks when PK measurement take place.

6.2 Dose modification and dose delay / or dose reduction

Investigators should follow the guidelines given below for dose modification of treatment with IMP; any deviation has to be previously discussed with the sponsor unless it concerns patient's safety. All dose changes or interruptions must be recorded on the appropriate CRF page.

6.2.1 Dose modification of Venetoclax - Haematological toxicity

TABLE 3: Dose modification of Venetoclax for haematological toxicity

**CTCAE Grade**	**Action**
Grade 1 or 2 neutropenia, thrombocytopenia or anaemia	No dose modification
Grade 3 to 4 neutropenia (neutrophils < 1.0 x 10^9/l), thrombocytopenia (platelets < 50 x 10^9/l), anaemia (< 8g/dL) or febrile neutropenia	Hold venetoclax until resolved to < Grade 3, at latest within 4 weeks since onset. Re-start venetoclax at a lower dose level (see below). In case of febrile neutropenia, GCS-F use is recommended as per local guidelines.

6.2.2 Dose modification of Venetoclax - Non-haematological toxicity

TABLE 4: Dose modification of Venetoclax for non-haematological toxicity

**CTCAE Grade**	**Action**
Any Grade 1 or 2 non-haematological toxicity	No dose modification
Any grade 3 to 4 non-haematological toxicity	Hold venetoclax until resolved to < Grade 3, at latest within 4 weeks since onset. Re-start venetoclax at a lower dose level (see below).

6.2.3 Dose modification of Obinutuzumab - Haematological toxicity

TABLE 5: Dose modification of Obinutuzumab for haematological toxicity

CTCAE Grade	Action
Grade 1 or 2 neutropenia, thrombocytopenia or anaemia	No dose modification
Grade 3 to 4 neutropenia or thrombocytopenia (= neutrophils < 1.0 x 10^9/l, platelets < 50 x 10^9/l) or febrile neutropenia	If considered *unrelated* to obinutuzumab, continue with obinutuzumab.<br>If considered at least possibly *related*, hold obinutuzumab until resolved to < Grade 3, at latest within 4 weeks since onset. Re-start at the same dose 1000mg (no dose modification). In case of febrile neutropenia, GCS-F use is recommended as per local guidelines.

6.2.4 Dose modification of Obinutuzumab – Non-haematological toxicity

TABLE 6: Dose modification of Obinutuzumab for non-haematological toxicity

CTCAE Grade	Action
Any Grade 1 or 2 non-haematological toxicity	No dose modification
Any grade 3 to 4 non-haematological toxicity	If considered *unrelated* to obinutuzumab, continue with obinutuzumab.<br>If considered at least possibly *related*, hold obinutuzumab until resolved to < Grade 3, at latest within 4 weeks since onset. Re-start at the same dose 1000mg (no dose modification).

6.3 Dosing groups of venetoclax

TABLE 7: Intra-patient dosing groups for venetoclax when re-starting after discontinuation for toxicity.

Dose level	Dose in mg/day
0	200
1	400
2	600

3	800
4	1000

In case of re-starting venetoclax, choose one dose level below the previously one under which the toxicity occurred. In case of repetitive toxicities requiring holding the drug, consider the same approach and de-escalate by one dosing level. In case toxicities do not resolve to < grade 3 within 4 weeks, the drug needs to be permanently stopped. Always contact the principle investigator in case of uncertainties.

6.4 Concomitant treatment/medication

6.4.1 Permitted prior/concomitant treatment/medication

Treatment with transfusions (red blood cells and platelets) and supportive care are permitted after the initiation of study treatment.

The patient must notify the investigational site of any new medication he/she starts taking after the start of the trial medication. All medications (other than IMP) and significant non-drug therapies (including physical therapy and blood transfusions) administered after the patient starts treatment with IMP must be listed in the CRF.

Furthermore, steroids at time of registration are allowed, but should be tapered as soon as treatment has started. If possible, patients should be off steroids at time of the first scan. Any experimental/investigational drug applied within a trial should be stopped at least 30 days before start of treatment.

6.4.2 Rescue medications, non-drug therapies or supportive care

Patients should receive treatment/medication appropriate to their clinical condition in an emergency. Transfusions for platelets and/or red blood cells should be given, as medically needed.

6.4.3 Prohibited and permitted concomitant therapy requiring caution and/or action

During the first 6 weeks when PK measurements and DLT assessments take place the following drug classes are prohibited:

- **CYP3A inhibitors and inducers**. Moderate (e.g. erythromycin, ciprofloxacin, diltiazem, dronedarone, fluconazole, verapamil) and strong (e.g. ketoconazole, conivaptan, clarithromycin, indinavir, itraconazole, lopinavir, ritonavir, telaprevir, posaconazole and voriconazole).
- **P-gp inhibitors** (e.g., amiodarone, captopril, carvedilol, cyclosporine, felodipine, quercetin, quinidine, ranolazine, ticagrelor) are also prohibited during the first 6 weeks.
- **BCRP inhibitors (e.g. digoxin, dabigatran, everolimus, sirolimus)**

After completion of the PK measurements and DLT period, moderate and strong CYP3A inhibitors as well as P-gp or BCRP inhibitors are allowed if clinically necessary, but should be

avoided and alternative drugs used. However, if such inhibitors are used, 50% (for moderate CYP3A, P-gp or BCRP inhibitors) and 75% (for strong CYP3A inhibitors) dose reductions of venetoclax are recommended, respectively. It is also recommended to avoid grapefruit products, Seville oranges, and starfruit during treatment with venetoclax, as they can contain inhibitors of CYP3A.

6.5 Unblinding of treatment assignment

Not applicable.

6.5.1 Premature unblinding

Not applicable.

6.5.2 Unblinding procedure and documentation

Not applicable.

6.5.3 Consequences for the patient's treatment

Not applicable.

6.6 Treatment after end of the trial

After end of the trial, the therapy of the PCNSL will be performed according to established international guideline, or, preferable, within another clinical trial.

7 Visit schedule and assessments

7.1 Flow and visit schedule

All assessments and treatments should be done according to the schedule of assessments. Where indicated, minor changes to the day of assessment (+/- 3 days) are allowed. Importantly, all data on e.g. physical examination, vital signs, laboratory measures will be collected and documented in the CRF (if not otherwise specified) on the level of the respective individual value (e.g. haemoglobin level in g/dl) every 3 weeks during induction treatment, 4 weeks after end of induction treatment assessment (EOITA) and thereafter every 8 weeks during maintenance treatment.

7.2 Visit and assessment windows

Screening evaluations have to be performed within 28 days prior to registration.

During the course of the trial, visits and test procedures should occur on schedule whenever possible. Visit C1D1, C1D3, C1D8, C1D15, and C2D7 must be performed on the scheduled day,

otherwise delays will be considered as protocol violation. All other visits during induction treatment that occur ± 3 days from the scheduled date will not constitute any protocol deviation. Visits during maintenance treatment may occur ± 5 days from the scheduled date (see study flow chart).

7.3 Screening and registration

The investigator is obliged to give the patient thorough information about the trial and the trial related assessments, and the patient should be given ample time to consider his or her participation. The investigator must not start any trial-specific procedure before Informed Consent Form (ICF) is signed and dated by both the patient (and impartial witness, if applicable) and the investigator. The investigator must keep the original signed ICF (a signed copy is given to the patient), (see section 15.3).

7.3.1 Screening

For patients who appear to meet the criteria for participation in the study, the Investigator will provide information to allow them to make an informed decision regarding their participation. Investigators will be expected to maintain a local Screening Log of all potential study candidates. This Log will include limited information about the potential candidate (e.g. year of birth and gender), the date and outcome of the screening process (e.g. enrolled into study, reason for ineligibility, or declined participation).

If informed consent is given, the Investigator will conduct a full screening evaluation to ensure that the patient satisfies all inclusion and exclusion criteria. A patient who gives written informed consent and who satisfies all the inclusion and exclusion criteria may be registered onto the study. Note that assessments conducted as standard of care do not require informed consent and may be provided as screening data if conducted within the stipulated number of weeks prior to registration.

7.3.2 Data to be collected on screening failures

Trial sites are required to document all screened patients on the screening log. If a screened subject is not registered, the reason(s) should be recorded in the source documents and on the screening log.

Screening failures are defined as patients who signed an ICF but failed to be registered in the study for any reason. These patients are to be documented on the subject screening and subject identification log (see section 5.1). In addition, screening failures will be documented in the screening CRF to capture the reason, in standardized fashion, for screening failure.

7.3.3 Assessments at screening

All patients will be screened prior to registration. Archival tissue from the first diagnosis (or relapse/progression if available) will be collected for translational studies. The following screening assessments will be **performed within 28 days prior to registration** unless stated otherwise in the section describing eligibility criteria and documented in the CRF unless stated otherwise (see section 7.5 for a precise definition of assessments):

- General
  - Verification of eligibility criteria
  - Written informed consent
- Demographic data
  - Medical history (including current symptoms)
  - Co-morbidities
  - Height & weight
  - Concomitant medications
- Blood tests
  - Haematology: Absolute leucocyte count, absolute neutrophil count, absolute lymphocyte count, haemoglobin, absolute platelet count
  - Biochemistry: ASAT, ALAT, bilirubin, gamma-GT, creatinine, estimated creatinine clearance, LDH
  - Clotting tests: PTT/INR
  - Virology for HIV, hepatitis B and C (not to be documented in CRF).
  - Pregnancy test
- Examinations
  - ECOG performance status
  - Clinical examination
  - Electrocardiography
  - Slit lamp examination
  - Bone marrow biopsy (can be omitted if PET/CT excludes involvement)
  - Lumbar puncture (Only in case of clinical suspicion for meningeal involvement)
- Radiology
  - Gadolinium-enhanced MRI scan of brain. MRI spine only in case of meningeal involvement
  - PET-CT whole body or contrast-enhanced CT neck till pelvis (to exclude systemic lymphoma). Performed as per local practice (to be documented in CRF)
- Other
  - Collection of paraffin embedded tissue collection for FoundationHemeOne Analyses (optional)
- Vital signs (not to be documented in CRF)

7.3.4 Registration

Patients considered eligible by the investigator once all screening procedures are complete will be registered to the study (see section 5.2). Registration should be performed as close as possible to the start of the study medication.

7.4 Treatment

Following inclusion in the trial and initiation of trial treatment (C1D1), the patient should visit the trial site at days 1, 3, 8, 15, 22, 28, 43, 64, 85, and 106 during induction treatment. On day 127, there will be the end of induction treatment assessment. After this, given eligibility of maintenance treatment is fulfilled, the patient will go on to maintenance treatment; there will be

one visit 4 weeks after end of induction treatment assessment (EOITA), thereafter visits every 8 weeks until end of maintenance treatment after 12 months. For details see sections below.

7.4.1 Assessments at Cycle 1 Day 1 (C1D1)

- Physical examination
- Vital signs*
- ECOG performance status
- Haematology/biochemistry
- PTT, INR
- PB collections for PK analyses (one pre-dose obinutuzumab, one post-dose (C1D2 if applicable; for further information see section 2.1) obinutuzumab)
- First treatment with obinutuzumab
- Start of treatment with venetoclax (daily)
- Concomitant medication
- Adverse events

*not to be documented in CRF

7.4.2 Assessments at Cycle 1 Day 3 (C1D3)

- Lumbar puncture for PK analyses
- PB collection for PK analyses
- Treatment with venetoclax (daily)
- Adverse events
- Concomitant medications

7.4.3 Assessments at Cycle 1 Day 8 (C1D8)

- Treatment with obinutuzumab
- Treatment with venetoclax (daily)
- Adverse events
- Concomitant medications

7.4.4 Assessments at Cycle 1 Day 15 (C1D15)

- Haematology/biochemistry
- PTT, INR
- Lumbar puncture for CSF PK
- PB collection for PK analyses
- Treatment with obinutuzumab
- Treatment with venetoclax (daily)
- Adverse events
- Concomitant medications

7.4.5 Assessments at Cycle 2 Day 1 (C2D1, +/- 3 days)

- DLT assessment$
- Physical examination
- Vital signs**
- ECOG Performance status
- Haematology/biochemistry
- PTT, INR
- MRI brain
- Treatment with obinutuzumab
- Treatment with venetoclax (daily)
- Concomitant medication
- Adverse events

**not to be documented in CRF
$ DLT form to be sent within 24 hours to CTU Freiburg

7.4.6 Assessments at Cycle 2 Day 7 (C2D7)

- Lumbar puncture for CSF PK
- PB collection for PK analyses
- Treatment with venetoclax (daily)
- Adverse events
- Concomitant medications

7.4.7 Assessments at Cycle 3 Day 1 (C3D1, +/- 3 days)

- Final DLT assessment$
- Completion of DLT assessment form (for patients without DLT until incl. day 43 see section 2.8.1 for DLT definition)
- Physical examination
- Vital signs*
- ECOG Performance status
- Haematology/biochemistry
- MRI brain
- Treatment with obinutuzumab
- Treatment with venetoclax (daily)
- Concomitant medication
- Adverse events

*not to be documented in CRF
$DLT form to be sent within 24 hours to CTU Freiburg

7.4.8 Assessments at Cycle 4 Day 1 (C4D1, +/- 3 days)

- Physical examination

- Haematology/biochemistry
- Vital signs**
- ECOG Performance status
- Treatment with obinutuzumab
- Treatment with venetoclax (daily)
- Concomitant medication
- Adverse events

**not to be documented in CRF

7.4.9 Assessments at Cycle 5 Day 1 (C5D1, +/- 3 days)

- Physical examination
- Haematology/biochemistry
- Vital signs**
- ECOG Performance status
- MRI brain
- Treatment with obinutuzumab
- Treatment with venetoclax (daily)
- Concomitant medication
- Adverse events

**not to be documented in CRF

7.4.10 Assessments at Cycle 6 Day 1 (C6D1, +/- 3 days)

- Physical examination
- Haematology/biochemistry
- Vital signs**
- ECOG Performance status
- Treatment with obinutuzumab
- Treatment with venetoclax (daily)
- Concomitant medication
- Adverse events

**not to be documented in CRF

7.4.11 Assessment at end of induction treatment assessment (EOITA) (Day 127, +/- 3 days, end of induction treatment visit)

- Physical examination
- Vital signs**
- ECOG Performance status
- Haematology/biochemistry
- MRI brain
- Slit lamp investigation (only if positive at screening)

- Lumbar puncture (only if positive at screening or in case of clinical suspicion for involvement)
- Treatment with venetoclax (daily)
- Concomitant medication
- Adverse events

**not to be documented in CRF

7.4.12 Assessments at Visits during maintenance treatment (once 4 weeks after end of induction treatment assessment (EOITA), thereafter every 8 weeks for 12 months counted from the first maintenance visit, +/- 5 days)

- Physical examination
- Vital signs**
- ECOG Performance status
- Haematology/biochemistry
- PB sample for PK analyses (only on 1st visit during maintenance 4 weeks after end of induction treatment assessment (EOITA))
- MRI brain (once 4 weeks after end of induction treatment assessment (EOITA), thereafter every 8 weeks for the first 6 months, afterwards every 12 weeks)
- Slit lamp investigation (only if positive at screening)
- Treatment with venetoclax (daily)
- Concomitant medication
- Adverse events

**not to be documented in CRF

7.4.13 Assessments during Follow-Up

After completing maintenance treatment, patients will have one follow-up visit 6 weeks (42 days) after the last visit of maintenance phase. No imaging is planned for that visit, however, in case of clinical worsening, it is to the discretion of the investigator to conduct imaging as indicated. In case of progression or notification of death, this will be documented in the respective CRF.

- Physical examination
- ECOG Performance status
- Haematology/biochemistry
- Adverse events (only up to 42 days after last intake of IMP)

7.4.14 Assessments at End of study visit (EOSV)

At the end of study, 6 weeks after the follow-up visit, the following examinations will be conducted:

- MRI brain
- Physical examination
- ECOG Performance status
- Haematology/biochemistry

7.4.15 Assessments if treatment or planned follow-up is discontinued prematurely

Patients who prematurely discontinue study treatment (for whatever reason; e.g. relapse, toxicity) or discontinue the planned follow-up (for whatever reason; e.g. relapse) should be scheduled for a visit (within 7 to 14 days in case of premature discontinuation of study treatment) at which the following will be performed (to be entered into TERMINATION OF STUDY CRF):

- Physical examination
- Vital signs**
- Performance status
- Haematology/biochemistry
- MRI brain (if not conducted within the last 4 weeks)
- Concomitant medication
- Adverse events

**not to be documented in CRF

In case patients discontinue prematurely during treatment or follow-up phase, they will be followed-up for subsequent treatment (type and date) and survival status. This information will be entered into the respective CRFs for CONCOMITANT MEDICATION (page 47) and SURVIVAL STATUS (page 52).

7.4.16 Routinely collected data after EOSV

To obtain lymphoma remission status and survival status after EOSV, we will use routinely collected health data collected in the patient health records at the respective hospital (50). Patients will give written informed consent that routinely collected data on above mentioned outcomes are planned being merged with the study specific database after closure of this study.

7.5 Assessments and specifications

7.5.1 Patient demographics

Patient's demographics comprise year of birth, sex and childbearing potential.

7.5.2 Medical history

At screening, relevant past medical history and assessments of any current medical conditions have to be documented in the CRF.

7.5.3 Pregnancy test and contraception

All female study patients or female partners of male study patients of childbearing potential must undergo a urine pregnancy test at screening to confirm eligibility in the trial and monthly thereafter as indicated in the flowchart (see Table 1). A woman is considered of childbearing potential, following menarche and until becoming post-menopausal unless permanently sterile.

Permanent sterilisation methods include hysterectomy, bilateral salpingectomy and bilateral oophorectomy.

In case of pregnancy female patients must immediately be withdrawn from the trial treatment, and the pregnancy must be reported to the sponsor on the appropriate form. Also in case of pregnancy of female partners of study patients they will be ask for their written consent to enable the sponsor to follow up their pregnancy in order to determine outcome, including spontaneous or voluntary termination, details of birth, and the presence/absence of any birth defects, congenital abnormalities or maternal and new-born complications.

Because venetoclax may cause embryo-fetal harm when administered to pregnant women, all female study patients of childbearing potential and all female partners of male study patients must use highly effective contraception up to 3 months after intake of last study drug. Methods that can achieve a failure rate of less than 1% per year when used consistently and correctly are considered highly effective. These methods include: Combined (estrogen and progestogen containing) hormonal contraception associated with inhibition of ovulation by either oral, intravaginal or transdermal application; progestogen-only hormonal contraception associated with inhibition of ovulation by either oral, injectable or implantable application; intrauterine device; intrauterine hormone-releasing system; bilateral tubal occlusion; vasectomised partner; or complete sexual abstinence. For this particular study, possible interaction with CYP3A4 must be considered when choosing the mode of highly-effective contraception.

The effect of the IMP on sperm is unknown. However, males are advised to not father children up to 3 months after last study drug intake and to use latex condoms at least up to 3 months after intake of last study drug.

7.5.4 Physical examination

Thorough physical/medical examination includes, but is not limited to cardiovascular, gastrointestinal, hepatobiliary, respiratory, skin, musculoskeletal, genitourinary/renal and other organ systems.

Physical examinations are recommended to be performed according to the flowchart; relevant findings concerning these examinations will only be documented in the CRF at screening. At other visits in case of clinically relevant abnormal findings, the investigator has to document an AE (please refer to section 10.1.1 for definitions) on the corresponding CRF-page.

7.5.5 Vital signs

Vital signs include heart rate and blood pressure (not to be documented in the CRF): In case of abnormal findings a corresponding AE has to be documented in the CRF

7.5.6 Height and weight

Height (cm) and weight (kg) will only be measured at screening. Results must be present on the patient's chart and recorded correspondingly into the CRF.

7.5.7 Performance status

The performance status will be done according to ECOG criteria within 28 days prior to registration and thereafter according to flow chart.

7.5.8 Laboratory tests

Blood tests and urinalysis include the parameter listed in the flowchart (see page 20) and will be scheduled according to the flowchart and as clinically indicated. All these routine blood tests will be conducted as per local hospital standards.

7.5.9 Electrocardiogram (ECG)

A 12-lead ECG will be performed at screening. Each ECG tracing will be kept in the source documents at the investigational site.

7.5.10 Imaging, MRI brain

Gadolinium enhanced MRI brain, as per local standards, has to be done at screening, on day 22, day 43, day 85, and at the end of induction treatment (day 127). During maintenance treatment, MRI brain is conducted 4 weeks after end of induction treatment assessment (EOITA) and thereafter every 8 weeks for the first 6 months, then every 12 weeks thereafter and at the EOSV 6 weeks after the end of the maintenance phase. Lymphoma response evaluation will follow the international IPCG criteria (51). There will be no central radiological review for this study.

7.5.11 DLT decision and CTU notification

The DLT decision (yes/no) has to be taken for the time period of the first 43 days (see section 2.8.1 for DLT definition).

Upon the occurrence of the first DLT in the patient, the CTU must be notified within 24 hours by means of the “DLT reporting form”. The complete form must be faxed by the investigator to the CTU to the SAE-Fax number (see section 10.2.3.1). In case the patient did not experience a DLT, this information must also be faxed with the “DLT reporting form” to the CTU on the day 22 and day 43 after start of the first IMP in the first cycle. The DLT from should be signed before infusion of obinutuzumab. Rapid transmission of these data to CTU is needed for correct determination as to whether to enrol a new patient onto the current, next higher, or next lower dose level.

In case of relapse/progression or patient’s death during the first 43 days the “DLT reporting form” has also to be immediately completed and faxed to the CTU.

7.5.12 Imaging, CT body or PET/CT

At screening, there will be one CT body (neck till pelvis) or PET/CT to exclude lymphoma manifestations outside the CNS compartment (part of eligibility criteria). Afterwards, no additional CT or PET/CT scans are required.

7.6 Additional biological specimen collection for translational program

For specifications of additional PB and CSF samples we refer to the clinical trial protocol of the respective translational study. Additional biological specimen collection will only be performed in patients who have given written informed consent by means of the respective patient informed consent.

7.6.1 Pharmacokinetics (PK)

CSF samples (4ml per sample CSF) and PB samples (9ml per sample PB) for PK will be collected for all patients enrolled in the study (for details please see the manual).

CSF samples will be collected on day 3, day 15 and day 28 in the morning; aim is 10:00 am (immediately pre-dose of venetoclax and obinutuzumab). The lumbar puncture will be conducted as per local guidelines.

PB samples will be taken on day 1 (pre-and post-infusion, post-infusion sample on day 2 if applicable, for further information see section 2.1), day 3 (pre-treatment), day 15 (pre-treatment), and day 28 (pre-treatment). There will be one last PK sample from PB 4 weeks after end of induction treatment assessment (EOITA). All PB pre-treatment samples will be taken at a maximum of 3 hours before venetoclax intake and infusion of obinutuzumab.

Complete instruction for sample collection, processing, handling and shipment will be provided in the laboratory manual.

7.6.1.1 Collection of blood and CSF saples for PK analyses

The procedure (lumbar puncture) to collect CSF will be conducted as per local guidelines. *It is of utmost importance to make sure that the CSF sample does not include any blood*. A detailed description is also provided in a *separate manual.* Blood PK samples will be taken by venous puncture.

Dedicated tubes for CSF collection and material to prepare the CSF collection tubes will be provided by the sponsor. To assure highest quality for PK measurement, blood and CSF collection must follow the provided *separate manual*.

7.6.2 Collection of archival tissue for mutational analyses

We will collect archival formalin-fixed tissue slides from all patients from the time of first diagnosis. Patient written informed consent needs to be signed before collecting these samples. If available, we will also collect formalin-fixed tissue slides of the most recent biopsy before entering the study. There is no mandatory re-biopsy for this study. Detailed instructions on sample collection, processing, handling and shipment will be provided in the laboratory manual.

8 Discontinuation criteria

8.1 Premature termination the entire trial

8.1.1 General note

The sponsor/coordinating investigator is under obligation to monitor the progress of the clinical trial with regard to safety-relevant developments and, if necessary, initiate the premature termination of a treatment arm or the entire clinical trial.

The entire clinical trial must be terminated prematurely if:

- the benefit-to-risk ratio for the patients changes markedly,
- the sponsor/coordinating investigator considers that the termination of the trial is necessary,
- indications arise that the trial patients' safety is no longer guaranteed,
- the question(s) addressed in the trial can be clearly answered on the basis of an interim safety assessment,
- the questions(s) addressed in the trial can be clearly answered on the basis of results of another trial on the same subjects,
- an insufficient recruitment rate makes a successful conclusion of the clinical trial unrealisable/no longer feasible.

If the clinical trial is prematurely terminated or suspended for any reason, the investigator should promptly inform the trial patients and ensure appropriate therapy and follow-up for the patients. Where required by the applicable regulatory requirements, the CA(s) and the EC(s) will also be informed. This is done by the sponsor.

8.1.2 Specific note for this study

Continuation of the study with the intended dose escalation depends on pre-defined DLTs. Therefore, after each dosing group (N=5) there will be a safety evaluation to ascertain the next dosing level. This safety evaluation will be conducted by the coordinating investigator and the medical trial coordinator based on the data collected and maintained within the central database at CTU Freiburg. Approval for continuation will be given by the coordinating investigator (see section responsibilities).

8.2 Premature termination of the trial at one of the trial sites

Both the investigator and the sponsor have the right to terminate the trial at one of the sites.

The clinical trial can be terminated prematurely at his site by the investigator if, for instance unforeseeable circumstances have arisen at the trial site which preclude the continuation of the clinical trial, the investigator considers that the resources for continuation are no longer available, the investigator considers that the continuation of the trial is no longer ethically or medically justifiable.

The sponsor/coordinating investigator can initiate the exclusion of a site from further participation if, for instance, patient recruitment is inadequate, serious problems arise with regard to the quality of the collected data which cannot be resolved.

Premature termination at one of the trial sites does not automatically mean a termination of the trial for already enrolled trial patients. A separate decision on further treatment must be made for each patient, depending on the overall situation. Adequate further treatment and follow-up of already enrolled trial patients must be ensured. The documentation of already enrolled trial patients will be reviewed for completeness and plausibility. Queries may be raised for further clarification before the site is closed. These queries must be answered properly by the site. The CA(s) and IEC(s) must be duly notified of the site´s closure, including reasons, within the specified period. The trial site concerned will be closed in stages by the CRA when a decision has been made on the further treatment of the patients concerned.

8.3 Discontinuation of trial treatment or trial participation for individual patients

It has to be distinguished if *trial treatment* of a patient has been stopped prematurely or if the *trial participation* of a patient was stopped prematurely.

In the case trial treatment of a patient has been stopped prematurely, further follow-up visits and the assessment of the trial endpoints are essential to enable an analysis of the full analysis set according to the intention-to-treat principle. Further visits, follow-up and documentation should always be striven for/ensured in this case. This includes the follow-up of AEs, the time of termination, the results available at that time and, if known, the documentation of the termination of treatment on the CRF and in the medical record, giving reasons, a final examination and documentation according to the protocol (if possible).

In the case trial participation of a patient was stopped prematurely, the conduct of further follow-up visits is no longer possible. The documentation should be completed as far as possible under these circumstances, e.g. a final examination and documentation according to the protocol (if possible), a documentation of the premature trial termination on the CRF and in the medical record, giving reasons, appropriate further treatment and follow-up outside the trial should be ensured; inform general practitioner of the termination, if necessary (provided that the patient agrees). In studies that assess the survival status, an attempt should at least be made to assess the patient's survival status by telephone follow-up (unless informed consent for documentation has been withdrawn).

8.3.1 Premature discontinuation of trial treatment

The trial patient can have his/her trial treatment terminated prematurely at any time, without having to give reasons.

The investigator responsible for the trial has the right to terminate the treatment of a patient according to the following conditions:

- Adverse events (including intercurrent illnesses) which preclude further treatment with the IMP or make further participation in the clinical trial inadvisable because the informational value of the trial results is impaired.
- Premature termination of the trial treatment is considered to be medically indicated, e.g. because it is subsequently found that inclusion/exclusion criteria were violated.

- Continuation of the trial treatment is unacceptable when the risks outweigh the benefits.
- Pregnancy
- Significant violations of the trial protocol or lack of compliance on the part of the patient (e.g. taking prohibited medication)
- Logistical reasons (patient changes his/her doctor or hospital or moves to another location)

8.3.2 Premature termination of trial participation

The trial patient can withdraw his/her consent at any time, without having to give reasons, and have his/her entire trial participation terminated prematurely. However, the prerequisite for this is that the patient actively terminates trial participation by withdrawing his/her consent for the follow-up and documentation.

The responsible investigator may only withdraw a patient from participation in the trial for the following reasons:

- Extreme circumstances arise which make any trial-relevant follow-up impossible

It will be left to the investigator’s clinical judgment to determine whether an adverse event is related and of sufficient severity to require the patient’s removal from treatment or from the study. A patient may also voluntarily withdraw from treatment due to what he or she perceives as an intolerable adverse event. If either of these situations arises, the patient should be strongly encouraged to undergo an end-of- study assessment and be under medical supervision until symptoms cease or the condition becomes stable.

9 Investigational medicinal products (IMPs)

9.1 Obinutuzumab background information

9.1.1 Preclinical data

The type II CD-20 antibody obinutuzumab (GA101) was specifically developed and glycoengineered to have increased direct cell death activity through increased affinity for Fc-delta RIIIa. Preclinical evidence suggests that obinutuzumab (GA101) is superior to rituximab in killing lymphoma cells in immune cell effector and whole-blood depletion assays and to have increased antitumor activity in human xenograft models (34).

9.1.2 Pharmacokinetics

A two-compartment PK model with linear and time-dependent clearance components accurately described the concentration–time course of obinutuzumab in patients with B-cell malignancies, with steadystate PK parameter values typical of monoclonal antibodies (52).

9.1.3 Pharmacodynamics

By binding to the cell surface protein CD 20, obinutuzumab induces direct cell death and enhanced antibody-dependent cellular cytotoxicity (with less complement-dependent cytotoxicity) (53,54).

9.1.4 Adverse reactions

The most common adverse events associated with obinutuzumab are infusions related reactions and B-cell depletion (36). For further details on adverse drug reactions please refer to the current version of corresponding IB.

9.2 Venetoclax background information

9.2.1 Preclinical data

Venetoclax is a selective, orally bioavailable small molecule inhibitor of BCL-2 (B-Cell Lymphoma 2), an anti-apoptotic protein in the BCL-2 family (17). The BCL-2 family of proteins includes pro-apoptotic and anti-apoptotic proteins. Anti-apoptotic proteins, such as BCL-2, block cell death by sequestering and preventing the activation of pro-apoptotic proteins (18,19). Overexpression of anti-apoptotic proteins of the BCL-2 family is implicated in tumour development (20–22).

9.2.2 Pharmacokinetics

The maximum plasma concentration achieved after oral administration occurred 5-8 hours after dose. Steady state maximum concentration with low-fat meal conditions at the 400 mg once daily dose was found to be 2.1 ± 1.1 ug/mL. It is recommended that Venetoclax be administered with a meal. The apparent volume of distribution for venetoclax is approximately 256–321 L. It is highly bound to human plasma protein. Within a concentration range of 1-30 µM (0.87-26 µg/mL), the fraction unbound in plasma was less than 0.01. Venetoclax is metabolized by CYP3A4/5 as proven by in-vitro studies. Those using the drug should not consume grapefruit products because they contain CYP3A inhibitors. Additionally, while using venetoclax it is not recommended to use other drugs which contain CYP3A inhibitors (i.e.: erythromycin, ciprofloxacin, diltiazem, dronedarone, fluconazole, verapamil). Venetoclax is excreted from the body via the fecal route (55).

9.2.3 Pharmacodynamics

Translational have shown that cell death in CLL cells in vitro and the depth of clinical responses are independent of deletion of chromosome 17p, TP53 mutation and TP53 function providing evidence that venetoclax kills CLL cells in a TP53-independent fashion by inhibition of BCL2 in patients (56).

9.2.4 Adverse reactions

The most common grade 3–4 adverse events associated with venetoclax are neutropenia, infections, anaemia, and thrombocytopenia, for further details on adverse drug reactions please refer to the current version of corresponding IB.

.

9.3 IMP(s) pharmaceutical characteristics

The IMP(s) used in this trial are characterised as follows, according to the applicable current reference safety information (RSI) of the IB:

Proprietary name:	Gazyvaro
Name of substance:	Obinutuzumab
Manufacturer:	ROCHE Pharma AG
Approved indications:	Chronic Lymphocytic Leukaemia (CLL)<br>Follicular Lymphoma (FL)
Dosage form:	Concentrate for solution for infusion.
Strength:	One vial of 40 mL concentrate contains 1,000 mg obinutuzumab

Proprietary name:	Venclyxto
Name of substance:	Venetoclax
Manufacturer:	AbbVie
Approved indications:	CLL
Dosage form:	Film-coated tablets
Strength:	10mg, 50mg and 100 mg
Total daily dose:	600mg, 800mg or 1000mg depending on patient’s dose level

9.4 Packaging and labelling

All drugs will be packaged and labelled by ROCHE Pharma AG. Medication labels will be in the local language and comply with GMP Annex 13 and legal requirements of each country.

9.5 Supply and ordering

ROCHE Pharma AG provides obinutuzumab and venetoclax free of charge. Each site will be supplied by ROCHE Pharma AG with both IMPs, which will be ordered via Email or Fax.

9.6 Receipt and storage

Trial medication must be received by a designated person at the investigational site/site pharmacy, handled and stored safely and properly, and kept in a secured location to which only the investigator/pharmacists and/or designated assistants have access.

The investigator will be responsible for ensuring the correct storage and sufficient stocks of the IMP at the site. Where allowed/required, the investigator may/should entrust the IMP, in whole or in part, to an appropriate pharmacist (to be designated in advance) or another appropriate individual who is under the supervision of the investigator.

The investigator or a pharmacist, or another appropriate individual who is designated by the investigator, should maintain records of the delivery of the IMP and the stocks at the study site.

9.7 Preparation of Obinutuzumab solution

Obinutuzumab will be delivered and prepared as described in the current SmPC/IB.

9.8 Dispensing

Trial medication will be dispensed by an authorised person at the investigator site.

Capsules for venetoclax including instructions for taking are dispensed by designated personnel on an outpatient basis. Patients will be provided with adequate supply of venetoclax for self-administration at home until at least their next scheduled visit at study site. Obinutuzumab will be given intravenously (possible in outpatient setting), 1000mg at each application.

9.9 Return and destruction

Unused IMP will be returned to Roche Pharma AG. Detailed instructions will be provided to the site by Roche Pharma AG. The investigator and/or pharmacist, or another appropriate individual who is designated by the investigator, should maintain records of the return of unused IMP(s) to Roche Pharma AG.

9.10 Drug compliance and accountability

Compliance will be assessed by the investigator and/or trial personnel at each patient visit and will be registered in the source document at each patient visit to accurately determine the patient's drug exposure throughout the trial.

The investigator or designee must maintain an accurate record of the shipment and dispensing of IMP in a drug accountability log. Drug accountability will be checked by the CRA during site visits as stated in the trial-specific monitoring manual.

Patients will be asked to return all unused IMP and packaging on a regular basis, at the end of the trial.

At the conclusion of the trial, and, as appropriate during the course of the trial, the investigator will return unused IMPs, packaging/labels and return a copy of the completed drug accountability log to Roche Pharma AG.

The investigator or designee should maintain records of the delivery of the IMP, the stocks at the study site, the use by the individual trial patients, and the return of unused IMP(s) to Roche Pharma AG. The investigator should ensure that the IMP is only used according to this protocol.

- The investigator bears the responsibility for the proper storage in an appropriate place to which unauthorised persons have no access.

- The investigator may only dispense the IMPs to patients who have been enrolled in the study. The dispensing of the IMPs to patients outside of this clinical trial is not permitted.
- The investigator or designee should explain the correct use of the IMPs to each trial patient and check at regular intervals that each patient is following the instructions correctly.

9.11 Treatment adherence

At each treatment visit the investigator or designee will remind the patient regarding:

- The importance of following study guidelines for adherence to daily venetoclax intake.
- Instructions about taking venetoclax including timing, storage, and importance of taking venetoclax, and what to do in the event of a missed dose.
- Notification that there will be a venetoclax count every 3 weeks (used/ empty/ unsealed/ damaged and/or unused packages have to be shown to the investigator at each clinic visit) during induction and at every visits during maintenance phase.
- Importance of calling the clinic if experiencing problems related to study product such as symptoms or loss or damage of packages.

10 Safety monitoring and reporting

10.1 Adverse Events (AEs)

10.1.1 Definition of AEs

An adverse event (AE) is any untoward medical occurrence in a patient administered any dose of a pharmaceutical product and which does not necessarily have to have a causal relationship with the use of the product. An AE can therefore be any unfavourable and unintended sign (including an abnormal laboratory finding), symptom, or disease temporally associated with the use of an IMP, whether or not related to the product.

- In order to monitor the conditions of the patients from the time the patients receive the first dose of IMP the investigator is requested to report any untoward clinical event on the AE-page of the CRF. Any untoward medical occurrence, which occurs after the period of patient follow-up defined in the protocol, is not considered an AE.
- Irrespective of any causal relationship, all AEs spontaneously reported by the patient or observed by the investigator will be continuously documented in the medical record and on the designated case report form (AE CRF page).
- All AEs must be described by diagnosis or, if an underlying diagnosis is not known, by symptoms or medically significant laboratory or instrumental abnormalities. The AEs will be documented as shown in section 10.1.3. Please note that medical or surgical procedures (e.g., tooth extraction, transfusion, surgery) performed are not AEs *per se*; the medical condition that leads to the procedure is an AE;
- Symptoms, medically significant laboratory, or instrumental (e.g. electrocardiographic) abnormalities of a pre-existing disease are not to be considered an AE. Occurrences

of new symptoms or laboratory or instrumental abnormalities, as well as worsening of pre-existing ones, are considered AEs.

- All AEs, no matter how intense, are to be followed up by the investigator in accordance with ICH-GCP until resolved or judged no longer clinically relevant, or in case of a chronic condition, until it is fully characterised.
- Overdose without clinical sequelae is not to be considered an AE. For the purposes of this study, an overdose is defined as a single dose of IMP that exceeds the prescribed dose for each age range.

10.1.2 AEs of special interest (AESI)

Adverse events of special interest are required to be reported by the investigator to the Sponsor immediately (i.e., no more than 24 hours after learning of the event). These events will be reported irrespective of seriousness (serious and non-serious AEs) following the procedure described below and will require enhanced data collection. Adverse events of special interest for this study include the following:

10.1.2.1 Potential drug-induced liver injury

Cases of potential drug-induced liver injury that include an elevated ALT or AST in combination with either an elevated bilirubin or clinical jaundice, as defined by Hy's law (57).

10.1.2.2 Suspected transmission of an infectious agent

Suspected transmission of an infectious agent by the study treatment, defined as: Any organism, virus, or infectious particle (e.g., prion protein transmitting transmissible spongiform encephalopathy), pathogenic or non-pathogenic, is considered an infectious agent. A transmission of an infectious agent may be suspected from clinical symptoms or laboratory findings that indicate an infection in a patient exposed to a medicinal product. This term applies only when a contamination of any of the study treatment components is suspected.

10.1.2.3 Tumour lysis syndrome (TLS)

Treatment with obinutuzumab and venetoclax can result in the rapid break down of tumour cells, resulting in AEs, as the body tries to get rid of the break-down products, leading to TLS. Symptoms or signs of TLS must be monitored e.g., fever, chills, tachycardia, nausea, vomiting, diarrhoea, diaphoresis, hypotension, muscle aches, weakness, paraesthesia, mental status changes, confusion, and seizures). However, the lymphoma burden in PCNSL patients is not comparable to patients with systemic NHL, therefore, the risk for TLS in PCNSL is considered very low.

10.1.2.4 Secondary malignancies

Second malignancies (related and unrelated to study treatment) will be reported indefinitely (even if the study has been closed) for patients who received obinutuzumab.

10.1.3 Documentation of AEs

Adverse events have to be documented in the CRF starting from the first administration and until 42 days after the last administration of IMPs. After this period and until end of the study only SAEs related to IMP(s) as per investigator's judgment and AESIs regardless of their relatedness will be documented on the CRF and reported to sponsor.

The following data will be collected on AEs:

- Characterization of the event (diagnosis; if not available, symptoms)
- Onset/end date
- Severity according to the current version of CTCAE (see section 19.1)
- Relationship to the IMP(s) (related/not related)

The expression "related" means, that there is evidence or argument to suggest a reasonable causal relationship between the event and the administration of the study drug, e.g. close temporal connection, exclusion of other causes.

The assessment "not related" is appropriate, if the AE is clearly or most likely explained by other causes even if a potential relationship between study drug and the SAE cannot be completely excluded.

- Serious / non-serious
- Action taken with IMP(s)
- Outcome

10.2 Serious Adverse Events (SAEs)

10.2.1 Definition of SAEs

A Serious Adverse Event (SAE) is any untoward medical occurrence that results in any of the following outcomes:

- Death,
- Life-threatening situation (patient is at immediate risk of death),
- Inpatient hospitalization or prolongation of existing hospitalization (excluding those for study therapy and/or assessments, placement of an indwelling catheter, social/convenience admissions, respite care, elective or pre-planned treatment/surgery)
- Persistent or significant disability/incapacity,
- Congenital anomaly/birth defect,
- Other, medically important condition: conditions which, in the investigator's opinion, may not be immediately life-threatening or result in hospitalization, but may jeopardize the patient's safety or may require intervention to prevent one of the other outcomes listed in the definition above, may also be considered serious. Examples of such conditions include: allergic bronchospasm requiring treatment in an emergency room or at home, unexpected convulsions (i.e. convulsions which cannot be explained by the underlying illness) that do not result in hospitalization, development of IMP dependency or drug abuse, suspected transmission of infectious agents by medicinal product, etc.

Clarification of SAEs:

- NOTE: The term “life-threatening” in the definition of “serious” refers to an event/reaction in which the patient was at risk of death at the time of the event/reaction; it does not refer to an event/ reaction which hypothetically might have caused death if it were more severe,

Patients may be hospitalised throughout the treatment phase of the trial according to the institution’s policy. Hospitalisation will, therefore, be only treated as a seriousness criterion if a medical event causes its prolongation ≥ 24 hours; this event should be considered a SAE.

10.2.2 Documentation of SAEs

All SAEs (with the exception of the special situation described below) that occur starting from the first administration and until 42 days after the last administration of the IMP(s) will be documented in the CRF and on the provided SAE reporting form.

After this period and until end of the study only SAEs related to IMP as per investigator’s judgment will be reported on the CRF and SAE form.

The SAE reporting form will be processed as described in the section below.

10.2.3 Investigator reporting requirements

10.2.3.1 Reporting policy

The following events must be reported by investigator to the sponsor:

- DLTs
- SAEs
- AEs of special Interests independent from their seriousness (see definition in section 10.1.2)

The events above must be reported by fax to the following address within 24 hours after knowledge by the investigator:

Pharmacovigilance
Clinical Trials Unit
Medical Center - University of Freiburg
Elsaesser Str. 2, 79110 Freiburg
SAE Fax No.
+49 761 270 – 74 390

If only limited data are initially available, a follow-up report is required. If new information including outcome becomes available or e.g. relationship to IMP(s) is reconsidered, a SAE follow-up report should be sent within 24 hours using the same procedure as for transmitting the initial SAE report (details will be provided in SAE reporting manual).

The Pharmacovigilance CTU Freiburg will forward each individual report (AESI and serious that are related to the IMPs within 15 calendar days; AESIs and serious that are unrelated to the IMPs within 30 calendar days) to Roche Pharma AG. Details are outlines in the SDEA.

10.2.3.2 Reporting of DLTs

The DLT assessment form has to be faxed to the CTU as soon as possible at the latest 24 hours after completion.

10.2.3.3 Specific protocol exceptions to expedited SAE reporting

As this trial involves patients suffering from haematological malignancies associated with significant mortality/morbidity, and that relapse/progression are trial endpoints (i.e. anticipated clinical outcomes) collected on the specific CRF pages and taking into consideration recommendations of the CIOMS working group VI concerning management of safety information from clinical trials, the following events have not to be notified to the sponsor as SAEs:

- Relapse / Progression:

A study patient's relapse or progression will be documented on specific CRFs pages and should not be communicated to the Sponsor as SAEs. Nevertheless, the investigator must fax the CRF page designated for relapse/progression documentation to the Pharmacovigilance CTU within 3 working days after knowledge.

10.2.3.4 Reporting of patient death

Please note that "death" is usually an SAE outcome and not an SAE *per se*. Only in cases where the clinical circumstances before the death are unknown (i.e. patient died without a determinable cause of death), then the diagnosis "death of unknown cause" itself should be reported as an SAE. In case of fatal outcome of an already-registered SAE, a follow-up notification must be done.

If a patient dies, the CRF page designated for death documentation must be faxed to the Pharmacovigilance CTU within 3 working days after knowledge.

According to section 12, subsection 6 GCP-V, in case of patient's death the investigator must submit on demand all information to the competent IEC, the other IEC(s) involved, the CA and the sponsor, that is required for the fulfilment of their duties (note that personal data must be transmitted using the trial-specific patient identification number, i.e. in pseudonymised form).

10.2.3.5 Reporting of premature treatment/study discontinuation

The investigator must fax the CRF page designated for premature treatment (051) and study discontinuation (043) to the PV CTU within 3 working days after knowledge to enable the sponsor to survey continuously safety of study participants and to fulfil legal reporting requirements.

10.2.4 Sponsor reporting requirements

The sponsor's reporting requirements are divided into expedited reporting and reporting that must be performed on request or annually.

10.2.4.1 Definition of SUSARs

The sponsor's expedited reporting requirements are particularly relevant to suspected unexpected serious adverse reactions (SUSARs). The definition is a combination of the definitions of serious adverse reaction (for seriousness criteria see section 10.2) and unexpected adverse reaction (adverse reaction: the nature or severity of which is not consistent with the applicable reference safety information in the IB for the IMP).

10.2.4.2 SUSAR/ circumstance requiring a review of the benefit/risk evaluation

The sponsor's expedited reporting requirements comprise the following:

- All SUSARs must be reported within 15 days after knowledge (section 13, subsection 2 GCP-V),
- All SUSARs that are life-threatening or result in death must be reported within 7 days after knowledge (section 13, subsection 3 GCP-V),
- All circumstances requiring a review of the benefit/risk evaluation of the IMP must be reported within 15 days after knowledge (e.g. expected serious adverse reaction with unexpected outcome, increased incidence of expected serious adverse reactions, SUSARs after the end of the patient's participation in the clinical trial, events in connection with the trial conduct or the development of the IMP which may affect the safety of the trial patients) (section 13, subsection 4 GCP-V).

10.2.4.3 Development Safety Update Report (DSUR)

In addition to the expedited reporting, the sponsor shall submit an annual report once a year or on request throughout the clinical trial period, according to section 13, subsection 6 GCP-V and ICH guideline E2F. The aim of the DSUR is to concisely describe all new safety information relevant for one or several clinical trial(s), to assess the safety conditions of subjects included in the concerned trial(s) and to evaluate whether the benefit / risk ratio is still favourable.

The Sponsor of the Study will be responsible for the preparation of the Development Safety Update Report (DSUR) for the Study and for the submission of the report to the regulatory authorities and Ethics Committees of the concerned Member States, where applicable. A copy of the DSUR will be sent to Roche as soon as reasonably possible after completion. Roche forwards an executive summary of the Roche DSUR upon request from the Sponsor.

10.2.5 Reviewing of SAEs

The sponsor or delegated will review every SAE. For all deaths, available autopsy reports and relevant medical reports should be faxed to the CTU.

Determination of the expectedness and relatedness of an SAE will be based on the contents of the IB/SmPCs for the IMPs.

If a patient is permanently withdrawn from the study because of a SAE, this information must be included in the initial or follow-up SAE Report as well as the End of Study CRF page, which has to be faxed to the PV CTU.

10.2.6 Case transmission verification of single case reports

The Parties will ensure that all single case reports have been adequately received by Roche Pharma AG. The Sponsor will send to Roche Pharma AG a periodic line-listing as defined in the Safety Data Exchange Agreement (SDEA) (addendum 3).

Confirmation of receipt should be received within the time period mutually agreed upon.

Following Case Transmission Verification, single case reports which have not been received by Roche shall be forwarded by the Sponsor to Roche within the timeline as defined in SDEA.

10.2.7 Pregnancies

Any pregnancy (female trial participant or female partner of male trial participant) that occurs during trial participation must be reported. To ensure patient safety each pregnancy must be reported to Pharmacovigilance CTU on the pregnancy reporting form within 24 hours of learning of its occurrence. The pregnancy should be followed up to determine outcome, including spontaneous or voluntary termination, details of birth, and the presence/absence of any birth defects, congenital abnormalities or maternal and new-born complications. These reports have to be sent to Roche within 30 calendar days of the awareness date.

10.2.8 Opening of next dosing cohort

Recruitment of patients will be on hold as soon as 5 patients available for DLT assessment are enrolled (after cohort 1 and after cohort 2). As soon as the 5th patient to the respective cohort has been enrolled, the CTU in Freiburg starts preparing all relevant data for the following DLT appraisal at latest on day 50 of the treatment schedule of the 5th patient. DLT appraisal should take place no longer than 2 weeks after day 50 of the 5th patient's treatment schedule. After DLT appraisal the coordinating investigator and the medical trial coordinator will approve escalation or de-escalation of the venetoclax dose and report the decision to the responsible CTU in Freiburg immediately via telephone AND Email.

11 Data handling and data management

11.1 Data confidentiality

Information about trial patients will be kept confidential and managed under the applicable laws and regulations. Those regulations require a signed patient authorization informing the patient of the following:

- what protected health information (PHI) will be collected from patients in this trial;
- who will have access to that information and why;
- who will use or disclose that information;
- the rights of a research patient to revoke their authorization for use of their PHI.

In the event that a patient revokes authorisation to collect or use PHI, the investigator, by regulation, retains the ability to use all information collected prior to the revocation of patient authorization. For patients that have revoked authorization to collect or use PHI, attempts should

be made to obtain permission to collect at least vital status (i.e. that the patient is alive) at the end of their scheduled trial phase.

The data collection system for this trial uses built-in security features to prevent unauthorised access to confidential participant information. Access to the system will be controlled by individually assigned user identification codes and passwords, made available only to authorised personnel who have completed prerequisite training.

11.2 Documentation of trial data

11.2.1 Documentation in medical records

The investigator will record the participation in the trial, the frequency of the trial visits, the relevant medical data, the concomitant treatment and the occurrence of adverse events in the medical record of each trial patient.

11.2.2 Documentation in CRF

The investigator, or a deputy who is designated by the investigator, will document the trial data on a trial-specific case report form (CRF) as promptly as possible.

The following CRF data are stated to be the source data in this study:

- severity of AEs and
- relationship of AEs to the IMP(s).

These data will be directly reported on the CRF pages.

Hard copy CRFs will be used in this trial. The hard copy CRFs consist of 2 OR 3-layer Non-Carbon-Required paper (NCR paper). All data collected during the trial will be entered on the trial-specific CRF pages by the responsible investigator, or an individual who is designated by the investigator. For further details regarding documentation please refer to the CRF completion instructions. Corrections and subsequent changes to CRF pages must be made according to the ICH-GCP guidelines provided in the CRF Completion instructions at the beginning of each CRF-folder.

11.3 Data management

The data management will be performed with DAMAST Version 9.2, a proprietary data management system based on the software package SAS, which is developed, validated and maintained by the Clinical Trials Unit (CTU). Details on data management (procedures, responsibilities, data corrections, if any, which may be made by data management staff themselves, etc.) will be described in a data management plan prior to the trial.

The technical specifications of the database and the data entry screens (variable names, attributes and data entry checks) will be described in a corresponding data description plan. Before any data entry is performed, the trial database will be validated. Double data entry will be performed by two different persons (with the exception of free text). The comparison of both entries and the resolution of discrepancies is only performed by trained staff. An audit trail will be

created to provide an electronic record of which data were entered or subsequently changed, by whom and when.

SAS software will be used to review the data for completeness, consistency and plausibility. The checks to be programmed will be specified beforehand in the data description plan. After running the check programs, the resulting queries will be sent to the investigator for review of his/her data. Answered queries will also be entered twice, verified and the updated data will then be transferred to the database. All programs which can be used to influence the data or the data quality will be validated (e.g. data validation programs, programs for CRF/query tracking, programs used for import of external data, etc.).

11.4 Data coding

Concomitant treatments or procedures entered into the database will be coded using the WHO Drug Reference List. Adverse events will be coded using the Medical dictionary for regulatory activities (MedDRA) terminology in its latest version.

12 Quality assurance

The sponsor is responsible for implementing and maintaining quality assurance and quality control systems with written SOPs to ensure that trials are conducted, data are generated, documented, and reported in compliance with the protocol, ICH-GCP, and the applicable regulatory requirement(s).

12.1 Monitoring procedure

Risk-based monitoring will be done according to ICH-GCP E6 and standard operating procedures (SOP) to verify that patients' rights and wellbeing are protected, reported trial data are accurate, complete and verifiable from source documents and that the trial is conducted in compliance with the currently approved protocol/amendment, with ICH-GCP and with the applicable regulatory requirements to ensure safety and integrity of clinical trial data.

The investigator will accept monitoring visits before, during and after the clinical trial. Prior to the trial, a site initiation visit at each site is conducted in order to train and introduce the investigators and their staff to the trial protocol, essential documents, handling of IMP and related trial specific procedures, ICH-GCP and national/local regulatory requirements.

During the trial, the CRA will visit the site regularly dependent on the recruitment rate and quality of data. During these on-site visits, the CRA verifies that the trial is conducted according to the trial protocol, trial specific procedures, ICH-GCP and national/local regulatory requirements. The presence of signed informed consents, eligibility of patients, primary endpoint, handling of IMP and documentation/reporting of safety data (e.g. AE/SAE) will be verified by the CRA. The CRA performs also source data verification and drug accountability to ensure that the clinical trial data which are recorded in the source data and CRFs are complete and accurate. Extent of source data verification and monitor visit frequency will be adapted for individual sites in case of lack of data quality or a high number of protocol violations. All trial specific monitoring procedures, monitoring visit frequency and extent of SDV will be predefined in a trial specific monitoring manual. The investigator must maintain source documents for each patient in the trial, consisting of case and visit notes (hospital or clinic medical records) containing demographic

and medical information, laboratory data, electrocardiograms, and the results of any other tests or assessments (see section 7). All information recorded on CRFs must be traceable to source documents in the patient's file. The investigator must also keep the original signed informed consent form (a signed copy is given to the patient).

The investigator must give the CRA access to all relevant source documents to confirm their consistency with the CRF entries.

12.2 Source data verification (SDV)

Source data as defined by ICH-GCP include original documents, data, and records such as hospital records, clinical and office charts, laboratory notes, memoranda, patients' diaries or evaluation checklists, pharmacy dispensing records, recorded data from automated instruments, copies or transcriptions certified after verification as being accurate copies, microfiches, photographic negatives, microfilm or magnetic media, X-rays, and records kept at the pharmacy, at the laboratories and at medico-technical departments involved in the clinical trial.

12.3 Auditing procedures and inspections

According to the ICH-GCP guidelines, audits may be performed as a quality measure. Audits may be conducted by the sponsor or an independent external party, inspections by CA(s).

The investigator needs to inform the CTU immediately of an inspection requested by a regulatory authority. The investigator is responsible for providing / giving access to source data/documents to auditors/inspectors.

13 Statistical planning and analysis

Before the start of the final analysis (see section 13.6) a detailed statistical analysis plan (SAP) will be prepared. If the SAP contains any changes to the analyses outlined in the trial protocol, they will be marked as such, and reasons for amendments will be given. All statistical programming for analysis will be performed with R or STATA.

13.1 Trial design

This is a non-randomized multicentre single arm open label dose-escalating phase IB trial. For details on trial design see section 3.1 of the protocol.

13.2 Objectives and endpoints

For details on endpoints see section 2 of the protocol.

13.3 Sample size calculation

Fifteen patients will be enrolled into this study to investigate the PK of venetoclax and obinutuzumab. We have chosen 15 patients as a convenient sample size (5 for each of the three intended dosing groups). In case a patient is not assessable during DLT time (during the first two

cycles) for whatever reason (e.g. death definitely unrelated to study drug, withdrawal of consent, lost to follow-up), we will replace the patient to guarantee that there are always five patients assessable for DLT (also see Sample Size) in each of the three dosing groups. Further details please see 3.1.

13.4 Definition of populations included in the analyses

For the primary endpoint PK, all patients with at least one paired sample (CSF and PB) will be considered for analysis.

We will conduct two analyses for efficacy. One is based on all registered patients in the denominator, irrespective whether treatment was applied, irrespective of whether they refused or discontinued the treatment or whether other protocol violations are revealed (intention-to-treat principle). Second analysis will be based on all patients who have received at least one treatment application for sensitivity analyses (as treated analysis). Safety analyses will be performed in the safety population which includes all patients who have received at least one dose of trial treatment.

13.5 Methods of analysis

Because of the small sample size, we will present the main outcome data (PK, response, DLT, survival endpoints) for each patient individually in appropriate tables or plots. Otherwise, data will be analysed and summarized as outlined below.

13.5.1 Patient demographics/other baseline characteristics

Demographic and other baseline data (including disease characteristics) will be summarised descriptively in total and grouped by dosing group.

Continuous data will be summarised by arithmetic mean, standard deviation, minimum, 25% quantile, median, 75% quantile, maximum, and the number of complete and missing observations. If appropriate, continuous variables can also be presented in categories.

Categorical data will be summarised by the total number of patients in each category and the number of missing values. Relative frequencies are displayed as valid % (number of patients divided by the number of patients with non-missing values).

13.5.2 Trial medication

Duration of trial treatment exposure, cumulative dose and dose intensity will be summarised by dosing group. The number of patients with dose changes/interruptions will be presented by dosing group, along with reasons for the dose change.

13.5.3 Concomitant medication

The concomitant medications will be summarised by ATC level 1/3/5. In each table, patients will be counted once, if they took at least one medication from the respective ATC level. The number

of patients and the percentage of the total number of patients in the respective population will be given.

13.5.4 Primary endpoint

We will describe the concentration (µg/ml) of venetoclax and obinutuzumab in each patient in the serum and CSF at the respective time points (as outlined above). The median (mena, range, interquartile range) of the concentration based on all evaluable patients at the respective time points will be computed separately for serum and CSF. To describe the CSF penetration of the respective compound, we will calculate the ratio of the CSF concentration / serum concentration.

Furthermore, we will summarize the frequency of patients (proportion with 95% confidence intervals) in which the concentration of venetoclax in the CSF at each time point will be 4% or more of the steady state blood concentration, in total and separately by dosing group. The same calculations will be done for obinutuzumab, but a cut-off at 1% or higher. The cut-off at 4% (for venetoclax) of the individual blood concentration is based on previous data (31) and corresponds to about 50nM (0.043µg/ml), which is the concentration reported inducing apoptosis in vitro experiments (58,59). Regarding obinutuzumab we expect to detect at least 1% of the average blood concentration in the individual patient. This is based on previous CNS PK data of the CD20 antibody rituximab (60).

All these calculations will be presented in total for all 15 patients, but also separately for the three dosing groups. We will, however, not conduct any statistical tests to compare the concentrations between the dosing groups.

13.5.5 Secondary endpoints for efficacy

For all secondary endpoints, we will primarily present all data based on the 15 patients, but we will also provide exploratory stratified analyses by dosing group. Patients with missing data regarding lymphoma response will be considered as non-responders. For time to event data (PFS, FFS, and OS), patients will be censored at last date of follow-up if they did no experience the respective event of interest beforehand. We will calculate respective survival probabilities at the 6, 9, and 12 months landmarks accompanied with 95% confidence intervals (CIs). Median survival times with 95% CIs will be calculated if reached. Again, because this study is entirely exploratory in nature, there will be no tests for hypothesis testing.

13.5.6 Safety parameters

All safety parameters (adverse events) will be listed by site and patient and displayed in summary tables. The adverse events (AEs) are displayed in summary tables by dosing group as follows:

The total number of AEs, the minimum, maximum and mean number of AEs per patient, the total number of follow-up days (number of days in the observation period), the number of AEs per FU-day (total number of AEs divided by the total by the number of follow-up days), the number of patients who had at least one AE, and the number of patients who stopped treatment due to AE will be given.

The incidence of AEs defined by preferred term (PT) according to MedDRA will be calculated as the number of patients who experienced at least one AE with the respective PT in percentage of

the total number of patients in the safety population. In the incidence tables the PTs will be grouped by system organ class (SOC) according to MedDRA. Additionally, the incidence of AEs defined by SOC will be calculated as the number of patients who experienced at least one AE in the respective SOC as percentage of the total number of patients in the safety population.

Each table will be produced for the following AE-sets:

- all AEs
- AEs being at least severe (>= Grade 3)
- AEs of special interest
- Serious Adverse Events (SAEs)
- SAEs leading to death
- AEs related to IMP
- AEs related to IMP being at least severe (toxicity)
- SAEs related to IMP
- SAEs related to IMP leading to death

Incidences of AEs will be calculated with 95%-confidence intervals.

Data on toxicity will be presented by the common toxicity criteria (CTCAE version 5.0) in frequencies (percentages); in total and stratified by dosing groups.

13.6 Safety analyses (DLT appraisal)

The escalation/de-escalation rules based on the BOIN approach (2) are described in 2.8.1 and 3.1: In total, there will be two safety assessments for DLTs: One after cohort 1 (first 5 patients) before preceding to cohort 2 and another one before preceding to cohort 3.

13.7 Exploratory analyses on gene alterations and response

We will investigate and describe the frequency and concordance of mutations detected in lymphoma tissue, peripheral blood, and CSF – intra patient patterns. In addition, we will explore the distribution of detected mutations among patient with at least PR during induction treatment compared to patients who never achieved PR. We will also investigate potential early detection of lymphoma associated mutations (that have cleared before) in the peripheral blood before progression on MRI. We will also investigate the frequency of cases where at the time of progression (as diagnosed on MRI) lymphoma specific mutations can be detected in peripheral blood.

13.8 Timepoints of comprehensive analyses for efficacy and safety

- The final statistical analysis including the final study report will be issued at latest 6 months after the last patient has completed maintenance treatment.

14 Scientific steering and data monitoring committees

14.1 Scientific steering committee (SSC)

The SSC will be built by the coordinating investigator, medical trial coordinator (both Klinikum Stuttgart) and the principle investigator from Freiburg University Hospital.

The coordinating investigator and the medical trial coordinator have been involved in the development of the protocol and will ensure transparent management of the trial according to the protocol through recommending and approving modifications as circumstances require. They will review all protocol amendments as appropriate.

In addition, the coordinating investigator and the medical trial coordinator will be involved in safety survey of the study. There will be regular (frequency dependent on recruitment rate) telephone conferences among all members of the SSC. All patients with a DLT defining event will be discussed and decisions on discontinuation, re-exposition with dose reduction or complete termination of study drug will be documented. A short-written protocol will be issued after each telephone conference and distributed via email to the study team as well as printed and filed in the respective patient specific study files on the site.

The coordinating investigator, medical trial coordinator are responsible to approve escalation to the next dosing level based on the pre-specified criteria as described in 2.8.2 und 13.6.

14.2 Data monitoring committee (DMC)

No Data Monitoring Committee (DMC) will be established.

15 Ethical and legal principles

15.1 Regulatory and ethical compliance

This clinical trial was designed, shall be implemented and reported in accordance with the ICH-GCP, with applicable local regulations (including European Directive 2001/20/EC), and with the ethical principles laid down in the Declaration of Helsinki.

Before initiating the clinical trial, the sponsor/coordinating investigator should submit the CTP and any required application(s) to the appropriate competent authority for review, acceptance, and/or permission, as required by the applicable regulatory requirements.

The protocol and the proposed informed consent form must be reviewed and approved by a properly constituted Independent Ethics Committee (IEC) before trial start. A signed and dated statement that the protocol and informed consent have been approved by the IEC must be available prior to initiation of the trial.

15.2 Responsibilities of the investigator

Before the start of the trial, the investigator is required to sign a protocol signature page confirming his/her agreement to conduct the trial in accordance with these documents and all of the instructions and procedures found in this protocol and to give access to all relevant data and

records to sponsor CRAs, auditors, sponsor Clinical Quality Assurance representatives, designated agents of sponsor, IECs and CA(s) as required.

15.3 Informed consent procedures

Before enrolment in the clinical trial, the patient will be informed that participation in the clinical trial is voluntary and that he/she may withdraw from the clinical trial at any time without having to give reasons and without penalty or loss of benefits to which the patient is otherwise entitled.

The treating physician will provide the patient with information about the treatment methods to be compared and the possible risks involved. At the same time, the nature, significance, implications, expected benefits and potential risks of the clinical trial and alternative treatment will be explained to the patient. During the informed consent discussion, the patient will also be informed about the insurance cover that exists and the insured's obligations. The patient will be given ample time and opportunity to obtain answers to any open questions. All questions relating to the clinical trial should be answered to the satisfaction of the patient and/or his/her legal representative. In addition, the patient will be given a patient information sheet which contains all the important information in writing.

The patient's written consent must be obtained before any trial-specific tests/treatments.<br>For this purpose, the written consent form will be personally dated and signed by the trial patient and the investigator conducting the informed consent discussion.

By signing the consent form, the patient agrees to voluntarily participate in the clinical trial and declares his/her intention to comply with the requirements of the clinical trial and the investigator's instructions during the clinical trial. By signing the form, the patient also declares that he/she agrees to the recording of personal data, particularly medical data, for the trial, to their storage and codified (“pseudonymised”) transmission to the sponsor, CA(s), and further agrees that authorised representatives of the sponsor, who are bound to confidentiality, national or foreign CA(s) may inspect his/her personal data, particularly medical data, which are held by the investigator.

After signing, the patient will be given one copy of the signed and dated written consent form and any other written information to be provided to the patients.

In the case of substantial amendments, the patient must be informed with an appropriate revised patient information/consent form. Changed trial procedures can only be carried out if they have been approved by the CA and the leading IEC, and if the patient has been appropriately informed and has given his/her written consent.

Fertile men and women of child bearing potential should be informed that taking the IMP may involve unknown risks to the foetus if pregnancy were to occur during the trial and agree that in order to participate in the trial they must adhere to the contraception requirement for the duration of the trial. The patients have to agree to data collection related to pregnancy and its outcome. If there is any question that the patient will not reliably comply, they should not be entered in the trial.

15.4 Patient insurance

Subject insurance (minimum: € 500,000 per subject) according to applicable law has been taken out with

(Policy-No: 85-415010-03019)

HDI, Global SE

Postfach 10 10 27

40001 Düsseldorf

for all subjects participating in the clinical trial.

The investigator, or an individual who is designated by the investigator, will inform the subject of the existence of the insurance, including the obligations arising from it. The trial subjects must be afforded access to insurance documents and provided with a copy of the general conditions of insurance on request.

15.5 Confidentiality of trial documents and patient records

The investigator must ensure anonymity of the patients; patients must not be identified by names in any documents submitted to sponsor. Signed informed consent forms and patient enrolment log must be kept strictly confidential to enable patient identification at the site.

All study-related information will be stored securely at the study site. All participant information will be stored in locked file cabinets in areas with limited access. All laboratory specimens, reports, data collection, process, and administrative forms will be identified by a coded identification number (see section 5.1) only to maintain participant confidentiality.

15.6 Financial disclosure

Financial disclosures should be provided by trial personnel who is directly involved in the treatment or evaluation of patients at the site - prior to trial start.

16 Trial documents and archiving

16.1 Trial documents/investigator site file

The investigator will be given an investigator site file containing all the necessary essential trial documents for the initiation of the trial at his/her site. The essential documents include a list on which the investigator will enter all appropriately qualified persons to whom he/she has delegated important trial-related tasks.

The investigator, or an individual who is designated by the investigator, will be responsible for the maintenance and completeness of the trial documents during the clinical trial. At the request of the CRA, auditor, IEC or CA(s), the investigator shall make available all the requested trial-related records for direct access. Essential documents must not be removed permanently.

16.2 Archiving

After completion of the clinical trial, the essential trial documents - as defined by ICH-GCP E6 section 8 - will be retained at the trial site for a sufficient period so that they will be available for audits and inspections by the CA(s).

The investigator will be responsible for the storage. The following retention periods will apply after the completion/termination of the clinical trial:

- The above-mentioned essential documents must be retained for at least 10 years (section 13, subsection 10 GCP-V).
- The medical records and other source documents must be retained for the longest possible period allowed by the hospital, the institution or the private practice.

The investigator/the institution should take measures to prevent accidental or premature destruction of these documents. The sponsor will notify the investigator in writing when the trial-related essential documents are no longer required.

16.3 Access to trial data

The steering group and all authors of the main publications of the trial result have access to the full trial dataset in order to ensure that the validity of the results can be verified.

17 Protocol adherence and amendments

17.1 Protocol adherence

Investigators ascertain they will apply due diligence to avoid protocol deviations. Under no circumstances should the investigator contact sponsor or its agents, if any, monitoring the trial to request approval of a protocol deviation, as no authorised deviations are permitted. If the investigator feels a protocol deviation would improve the conduct of the trial this must be considered a protocol amendment, and unless such an amendment is agreed upon by sponsor and approved by the IEC it cannot be implemented.

17.2 Amendments to the protocol

Any change or addition to the protocol can only be made in a written protocol amendment that must be approved by sponsor, CA where required, and the IEC.

Only changes of the protocol that are required for patient safety may be implemented prior to IEC approval.

Regardless of the need for approval of formal protocol amendments, the investigator is expected to take immediate action required for the safety of any patient included in this trial, even if this action represents a deviation from the protocol. In such cases, the sponsor has to be notified as soon as possible of this action; the IEC should be informed correspondingly.

Information regarding important protocol modifications will be provided in due time to further relevant parties (e.g. investigators, trial participants, trial registries, journals).

18 Administrative Agreements

18.1 Financing of the trial and role of funders

This is an investigator-initiated study funded by Roche Pharma AG and AbbVie. Of note, the funders were involved into the design of the trial, which was mainly driven by the academic investigators. However, Roche Pharma AG and AbbVie will have no control over conduct, data analysis and interpretation, manuscript writing, and dissemination of trial results.

18.2 Trial agreement- investigator compensation

According to ICH-GCP 4.9.6, a trial agreement on the conduct of the clinical trial and the compensation for conducting the trial will be signed between the sponsor of the clinical trial and the investigators including their heads of administration.

18.3 Reimbursement of trial patients

There is no payment planned for patients.

18.4 Trial reports

After completion of the analysis by Benjamin Kasenda, the coordinating investigator will prepare and sign the final integrated medical and statistical report including a synopsis of the results, a publication containing the results of the study jointly with all members of the steering committee.

Except when required by law, no one will disclose a result of the clinical trial to third parties unless all parties involved have first agreed on the results of the analysis and their interpretation.

The final trial report will be written and signed in co-operation between the coordinating investigator and the CTU of Medical Centre - University of Freiburg.

18.5 Clinical trials registry

The sponsor ensures that the key design elements of this protocol is be posted in publicly accessible clinical trials registries:

European clinical trials network (https://www.clinicaltrialsregister.eu/): 2017-003690-33

ClinicalTrialsGov (https://clinicaltrials.gov/): To be determined

18.6 Publication of trial protocol and results

The trial protocol will be published in form of a rationale and design paper authored by the members of the steering committee.

Results from this study will be published irrespective of whether the study will be completed (that is reaching the target sample size of 15 patients) and irrespective of the nature of findings. The academic principle investigators (Prof. Dr. Illerhaus and PD Dr. med. Dr. phil. Kasenda) take the lead in preparing the study report and the resulting publication. Abbvie/Roche will have the

opportunity to review and comment on any abstract (14 days before submission) or manuscript (21 days before submission), but will have no right to withhold submissions for publication or other forms of distribution such as congress reports, online publications, or other forms of dissemination of results (e.g. on clinicaltrials.gov).

18.7 Authorship in publications of trial protocol and results

Personnel of Roche Pharma AG or AbbVie or any other for-profit institution involved in the study will not be on the author list of any publication, however, if appropriate, names of that staff can be mentioned in the acknowledgement section.

Authorship of any publications resulting from this study will be determined on basis of the Uniform Requirement for Manuscripts Submitted to Biomedical Journals (International Committee of Medical Journal Editors, 2005), which states:

- Authorship credit should be based on (1) substantial contributions to conception and design, acquisition of data, or analysis and interpretation of data; (2) drafting the article or revising it critically for important intellectual content; (3) final approval of the version to be published. Authors should meet conditions 1, 2, and 3.
- When a large, multicentre group has conducted the work, the group should identify the individuals who accept direct responsibility for the manuscript. These individuals should fully meet the criteria for authorship defined above.
- Acquisition of funding, collection of data, or general supervision of the research group, alone, does not justify authorship.
- All persons designated as authors should qualify for authorship, and all those who qualify should be listed.
- Each author should have participated sufficiently in the work to take public responsibility for appropriate portions of the content.

19 References

1. Abrey LE, Batchelor TT, Ferreri AJM, Gospodarowicz M, Pulczynski EJ, Zucca E, et al. Report of an international workshop to standardize baseline evaluation and response criteria for primary CNS lymphoma. J Clin Oncol Off J Am Soc Clin Oncol. 2005 Aug 1;23(22):5034–43.

2. Yuan Y, Hess KR, Hilsenbeck SG, Gilbert MR. Bayesian Optimal Interval Design: A Simple and Well-Performing Design for Phase I Oncology Trials. Clin Cancer Res Off J Am Assoc Cancer Res. 2016 Sep 1;22(17):4291–301.

3. Panageas KS, Elkin EB, DeAngelis LM, Ben-Porat L, Abrey LE. Trends in survival from primary central nervous system lymphoma, 1975-1999: a population-based analysis. Cancer. 2005 Dec;104(11):2466–72.

4. Olson JE, Janney C a, Rao RD, Cerhan JR, Kurtin PJ, Schiff D, et al. The continuing increase in the incidence of primary central nervous system non-Hodgkin lymphoma: a surveillance, epidemiology, and end results analysis. Cancer. 2002 Oct;95(7):1504–10.

5. Makino K, Nakamura H, Kino T, Takeshima H, Kuratsu J-I. Rising incidence of primary central nervous system lymphoma in Kumamoto, Japan. Surg Neurol. 2006 Nov;66(5):503–6.

6. Ferreri AJ, Reni M, Foppoli M, Martelli M, Pangalis G a, Frezzato M, et al. High-dose cytarabine plus high-dose methotrexate versus high-dose methotrexate alone in patients with primary CNS lymphoma: a randomised phase 2 trial. The Lancet. 2009 Nov;374(9700):1512–20.

7. Illerhaus G, Marks R, Ihorst G, Guttenberger R, Ostertag C, Derigs G, et al. High-dose chemotherapy with autologous stem-cell transplantation and hyperfractionated radiotherapy as first-line treatment of primary CNS lymphoma. J Clin Oncol. 2006 Aug;24(24):3865–70.

8. Illerhaus G, Müller F, Feuerhake F, Schäfer A-O, Ostertag C, Finke J. High-dose chemotherapy and autologous stem-cell transplantation without consolidating radiotherapy as first-line treatment for primary lymphoma of the central nervous system. Haematologica. 2008 Jan;93(1):147–8.

9. Schorb E, Kasenda B, Atta J, Kaun S, Morgner A, Hess G, et al. Prognosis of patients with primary central nervous system lymphoma after high-dose chemotherapy followed by autologous stem cell transplantation. Haematologica. 2013 May 8;98(5):765–70.

10. Omuro A, Correa DD, DeAngelis LM, Moskowitz CH, Matasar MJ, Kaley TJ, et al. R-MPV followed by high-dose chemotherapy with TBC and autologous stem-cell transplant for newly diagnosed primary CNS lymphoma. Blood. 2015 Feb 26;125(9):1403–10.

11. Kasenda B, Schorb E, Fritsch K, Finke J, Illerhaus G. Prognosis after high-dose chemotherapy followed by autologous stem-cell transplantation as first-line treatment in primary CNS lymphoma--a long-term follow-up study. Ann Oncol Off J Eur Soc Med Oncol ESMO. 2012 Oct 3;23(10):2670–5.

12. Reni M, Mason W, Zaja F, Perry J, Franceschi E, Bernardi D, et al. Salvage chemotherapy with temozolomide in primary CNS lymphomas: preliminary results of a phase II trial. Eur J Cancer. 2004 Jul;40(11):1682–8.

13. Raizer JJ, Rademaker A, Evens AM, Rice L, Schwartz M, Chandler JP, et al. Pemetrexed in the treatment of relapsed/refractory primary central nervous system lymphoma. Cancer. 2012 Aug 1;118(15):3743–8.

14. Voloschin AD, Betensky R, Wen PY, Hochberg F, Batchelor T. Topotecan as salvage therapy for relapsed or refractory primary central nervous system lymphoma. J Neurooncol. 2008 Jan;86(2):211–5.

15. Nayak L, Abrey LE, Drappatz J, Gilbert MR, Reardon DA, Wen PY, et al. Multicenter phase II

study of rituximab and temozolomide in recurrent primary central nervous system lymphoma. Leuk Lymphoma. 2013 Jan 1;54(1):58–61.

16. Ferreri AJM. How I treat primary CNS lymphoma. Blood. 2011 Jul 21;118(3):510–22.

17. Souers AJ, Leverson JD, Boghaert ER, Ackler SL, Catron ND, Chen J, et al. ABT-199, a potent and selective BCL-2 inhibitor, achieves antitumor activity while sparing platelets. Nat Med. 2013 Feb;19(2):202–8.

18. Del Gaizo Moore V, Brown JR, Certo M, Love TM, Novina CD, Letai A. Chronic lymphocytic leukemia requires BCL2 to sequester prodeath BIM, explaining sensitivity to BCL2 antagonist ABT-737. J Clin Invest. 2007 Jan;117(1):112–21.

19. Plati J, Bucur O, Khosravi-Far R. Apoptotic cell signaling in cancer progression and therapy. Integr Biol. 2011;3(4):279.

20. Cory S, Huang DCS, Adams JM. The Bcl-2 family: roles in cell survival and oncogenesis. Oncogene. 2003 Nov 24;22(53):8590–607.

21. Adams JM, Cory S. The Bcl-2 apoptotic switch in cancer development and therapy. Oncogene. 2007 Feb 26;26(9):1324–37.

22. Reed JC. Bcl-2-family proteins and hematologic malignancies: history and future prospects. Blood. 2008 Apr 1;111(7):3322–30.

23. Alizadeh AA, Eisen MB, Davis RE, Ma C, Lossos IS, Rosenwald A, et al. Distinct types of diffuse large B-cell lymphoma identified by gene expression profiling. Nature. 2000 Feb 3;403(6769):503–11.

24. Montesinos-Rongen M, Küppers R, Schlüter D, Spieker T, Van Roost D, Schaller C, et al. Primary central nervous system lymphomas are derived from germinal-center B cells and show a preferential usage of the V4-34 gene segment. Am J Pathol. 1999 Dec;155(6):2077–86.

25. Montesinos-Rongen M, Zühlke-Jenisch R, Gesk S, Martín-Subero JI, Schaller C, Van Roost D, et al. Interphase cytogenetic analysis of lymphoma-associated chromosomal breakpoints in primary diffuse large B-cell lymphomas of the central nervous system. J Neuropathol Exp Neurol. 2002 Oct;61(10):926–33.

26. Davis RE, Brown KD, Siebenlist U, Staudt LM. Constitutive nuclear factor kappaB activity is required for survival of activated B cell-like diffuse large B cell lymphoma cells. J Exp Med. 2001 Dec 17;194(12):1861–74.

27. Courts C, Montesinos-Rongen M, Martin-Subero JI, Brunn A, Siemer D, Zühlke-Jenisch R, et al. Transcriptional profiling of the nuclear factor-kappaB pathway identifies a subgroup of primary lymphoma of the central nervous system with low BCL10 expression. J Neuropathol Exp Neurol. 2007 Mar;66(3):230–7.

28. Montesinos-Rongen M, Siebert R, Deckert M. Primary lymphoma of the central nervous system: just DLBCL or not? Blood. 2009 Jan 1;113(1):7–10.

29. Chapuy B, Roemer MGM, Stewart C, Tan Y, Abo RP, Zhang L, et al. Targetable genetic features of primary testicular and primary central nervous system lymphomas. Blood. 2016 Feb 18;127(7):869–81.

30. Stilgenbauer S, Eichhorst B, Schetelig J, Coutre S, Seymour JF, Munir T, et al. Venetoclax in relapsed or refractory chronic lymphocytic leukaemia with 17p deletion: a multicentre, open-label, phase 2 study. Lancet Oncol. 2016 May;2045(16):1–11.

31. Roberts AW, Davids MS, Pagel JM, Kahl BS, Puvvada SD, Gerecitano JF, et al. Targeting BCL2

with Venetoclax in Relapsed Chronic Lymphocytic Leukemia. N Engl J Med. 2016 Jan 28;374(4):311–22.

32. Batchelor TT, Grossman SA, Mikkelsen T, Ye X, Desideri S, Lesser GJ. Rituximab monotherapy for patients with recurrent primary CNS lymphoma. Neurology. 2011 Mar;76(10):929–30.

33. Ferreri AJM, Cwynarski K, Pulczynski E, Ponzoni M, Deckert M, Politi LS, et al. Chemoimmunotherapy with methotrexate, cytarabine, thiotepa, and rituximab (MATRix regimen) in patients with primary CNS lymphoma: results of the first randomisation of the International Extranodal Lymphoma Study Group-32 (IELSG32) phase 2 trial. Lancet Haematol. 2016 May;3(5):e217-27.

34. Mössner E, Brünker P, Moser S, Püntener U, Schmidt C, Herter S, et al. Increasing the efficacy of CD20 antibody therapy through the engineering of a new type II anti-CD20 antibody with enhanced direct and immune effector cell-mediated B-cell cytotoxicity. Blood. 2010 Jun 3;115(22):4393–402.

35. Sehn LH, Goy A, Offner FC, Martinelli G, Caballero MD, Gadeberg O, et al. Randomized Phase II Trial Comparing Obinutuzumab (GA101) With Rituximab in Patients With Relapsed CD20+ Indolent B-Cell Non-Hodgkin Lymphoma: Final Analysis of the GAUSS Study. J Clin Oncol Off J Am Soc Clin Oncol. 2015 Oct 20;33(30):3467–74.

36. Morschhauser FA, Cartron G, Thieblemont C, Solal-Céligny P, Haioun C, Bouabdallah R, et al. Obinutuzumab (GA101) monotherapy in relapsed/refractory diffuse large b-cell lymphoma or mantle-cell lymphoma: results from the phase II GAUGUIN study. J Clin Oncol Off J Am Soc Clin Oncol. 2013 Aug 10;31(23):2912–9.

37. Flinn IW, Brunvand MM, Choi MY, Dyer MJ, Gribben JG, Hillmen P, et al. Safety and Efficacy of a Combination of Venetoclax (GDC-0199/ABT-199) and Obinutuzumab in Patients with Relapsed/Refractory or Previously Untreated Chronic Lymphocytic Leukemia - Results from a Phase 1b Study. Blood. 2015;ASH.

38. Flinn I, Brunvand MM, Dyer MJ, Hillmen P, Jones J, Li Y, et al. Preliminary Results of a Phase 1b Study (GP28331) Combining Venetoclax (GDC-0199/ABT-199) and Obinutuzumab in Patients With Relapsed/Refractory or Previously Untreated Chronic Lymphocytic Leukemia. In: ASH. 2014.

39. FDA. VENCLEXTA™ (venetoclax) tablets, for oral use Initial U.S. Approval: 2016 [Internet]. 2016 [cited 2017 Mar 1]. Available from: http://www.accessdata.fda.gov/drugsatfda_docs/label/2016/208573s000lbl.pdf

40. Davids MS, Roberts AW, Seymour JF, Pagel JM, Kahl BS, Wierda WG, et al. Phase I First-in-Human Study of Venetoclax in Patients With Relapsed or Refractory Non-Hodgkin Lymphoma Patient Demographic and Clinical Characteristics. 2017;35(8).

41. Jahnke K, Thiel E, Martus P, Herrlinger U, Weller M, Fischer L, et al. Relapse of primary central nervous system lymphoma: clinical features, outcome and prognostic factors. J Neurooncol. 2006 Nov;80(2):159–65.

42. Raizer JJ, Rademaker A, Evens AM, Rice L, Schwartz M, Chandler JP, et al. Pemetrexed in the treatment of relapsed/refractory primary central nervous system lymphoma. Cancer. 2011 Dec 16;

43. Soussain C, Hoang-Xuan K, Taillandier L, Fourme E, Choquet S, Witz F, et al. Intensive chemotherapy followed by hematopoietic stem-cell rescue for refractory and recurrent primary CNS and intraocular lymphoma: Société Française de Greffe de Moëlle Osseuse-Thérapie Cellulaire. J Cinical Oncol. 2008 May;26(15):2512–8.

44. Roth P, Hoang-Xuan K. Challenges in the treatment of elderly patients with primary central nervous system lymphoma. Curr Opin Neurol. 2014 Dec 13;27(6):697–701.

45. Fischer K, Al-Sawaf O, Fink A-M, Dixon M, Bahlo J, Warburton S, et al. Venetoclax and

obinutuzumab in chronic lymphocytic leukemia. Blood. 2017 Mai;129(19):2702–2705.

46. Grommes C, Pastore A, Palaskas N, Tang SS, Campos C, Schartz D, et al. Ibrutinib Unmasks Critical Role of Bruton Tyrosine Kinase in Primary CNS Lymphoma. Cancer Discov [Internet]. 2017 Jun; Available from: http://www.ncbi.nlm.nih.gov/pubmed/28619981

47. Lionakis MS, Dunleavy K, Roschewski M, Widemann BC, Butman JA, Schmitz R, et al. Inhibition of B Cell Receptor Signaling by Ibrutinib in Primary CNS Lymphoma. Cancer Cell. 2017 Jun;31(6):833–843.e5.

48. He J, Abdel-Wahab O, Nahas MK, Wang K, Rampal RK, Intlekofer AM, et al. Integrated genomic DNA/RNA profiling of hematologic malignancies in the clinical setting. Blood. 2016 Jun 16;127(24):3004–14.

49. Abrey LE, Batchelor TT, Ferreri AJM, Gospodarowicz M, Pulczynski EJ, Zucca E, et al. Report of an international workshop to standardize baseline evaluation and response criteria for primary CNS lymphoma. J Clin Oncol Off J Am Soc Clin Oncol. 2005 Aug 1;23(22):5034–43.

50. Fitzpatrick T, Perrier L, Shakik S, Cairncross Z, Tricco AC, Lix L, et al. Assessment of Long-term Follow-up of Randomized Trial Participants by Linkage to Routinely Collected Data: A Scoping Review and Analysis. JAMA Netw Open. 2018 Dec 21;1(8):e186019.

51. Abrey LE, Batchelor TT, Ferreri AJM, Gospodarowicz M, Pulczynski EJ, Zucca E, et al. Report of an international workshop to standardize baseline evaluation and response criteria for primary CNS lymphoma. J Clin Oncol. 2005 Aug;23(22):5034–43.

52. Gibiansky E, Gibiansky L, Carlile DJ, Jamois C, Buchheit V, Frey N. Population Pharmacokinetics of Obinutuzumab (GA101) in Chronic Lymphocytic Leukemia (CLL) and Non-Hodgkin's Lymphoma and Exposure-Response in CLL. CPT Pharmacomet Syst Pharmacol. 2014 Oct 29;3:e144.

53. Dalle S, Reslan L, Besseyre de Horts T, Herveau S, Herting F, Plesa A, et al. Preclinical studies on the mechanism of action and the anti-lymphoma activity of the novel anti-CD20 antibody GA101. Mol Cancer Ther. 2011 Jan;10(1):178–85.

54. Alduaij W, Ivanov A, Honeychurch J, Cheadle EJ, Potluri S, Lim SH, et al. Novel type II anti-CD20 monoclonal antibody (GA101) evokes homotypic adhesion and actin-dependent, lysosome-mediated cell death in B-cell malignancies. Blood. 2011 Apr 28;117(17):4519–29.

55. Venetoclax FDA Label [Internet]. Available from: https://www.accessdata.fda.gov/drugsatfda_docs/label/2016/208573s000lbl.pdf

56. Anderson MA, Deng J, Seymour JF, Tam C, Kim SY, Fein J, et al. The BCL2 selective inhibitor venetoclax induces rapid onset apoptosis of CLL cells in patients via a TP53 independent mechanism. Blood [Internet]. 2016 Apr; Available from: http://www.ncbi.nlm.nih.gov/pubmed/27069256

57. FDA. Guidance for Industry Drug-Induced Liver Injury: Premarketing Clinical Evaluation [Internet]. Available from: https://www.fda.gov/media/116737/download

58. Pan R, Hogdal LJ, Benito JM, Bucci D, Han L, Borthakur G, et al. Selective BCL-2 inhibition by ABT-199 causes on-target cell death in acute myeloid leukemia. Cancer Discov. 2014 Mar;4(3):362–75.

59. Cinar M, Rosenfelt F, Rokhsar S, Lopategui J, Pillai R, Cervania M, et al. Concurrent inhibition of MYC and BCL2 is a potentially effective treatment strategy for double hit and triple hit B-cell lymphomas. Leuk Res. 2015 Jul;39(7):730–8.

60. Rubenstein JL, Combs D, Rosenberg J, Levy A, McDermott M, Damon L, et al. Rituximab therapy for CNS lymphomas: targeting the leptomeningeal compartment. Blood. 2003 Jan 15;101(2):466–

8.

19.1 Relevant Guidelines and Laws

Declaration of Helsinki	https://www.wma.net/policies-post/wma-declaration-of-helsinki-ethical-principles-for-medical-research-involving-human-subjects/
ICH E6 - GCP Guideline	https://www.ich.org/products/guidelines/efficacy/efficacy-single/article/integrated-addendum-good-clinical-practice.html
ICH E8 – General considerations for clinical trials	http://www.ich.org/products/guidelines/efficacy/efficacy-single/article/general-considerations-for-clinical-trials.html
ICH E2F - DSUR	http://www.ich.org/fileadmin/Public_Web_Site/ICH_Products/Guidelines/Efficacy/E2F/Step4/E2F_Step_4.pdf
EMA Guidelines	http://www.ema.europa.eu/ema/index.jsp?curl=pages/regulation/landing/human_medicines_regulatory.jsp&mid=WC0b01ac058001ff89
AMG/GCP-V	http://www.gesetze-im-internet.de
Common Terminology Criteria for Adverse Events (CTCAE) current version	http://ctep.cancer.gov/protocolDevelopment/electronic_applications/ctc.htm
Recommendations related to contraception and pregnancy testing in clinical trials- Heads of Medicines Agencies (HMA)	http://www.hma.eu/fileadmin/dateien/Human_Medicines/01-About_HMA/Working_Groups/CTFG/2014_09_HMA_CTFG_Contraception.pdf
